# Supplementary material for: Migration direction in a songbird explained by two loci
Source: Nat Commun. 2023 Jan 11;14:165. doi: 10.1038/s41467-023-35788-7 (PMC9834303; doi:10.1038/s41467-023-35788-7)
Supplement: Supplementary file 1 — Supplementary_Information [file 41467_2023_35788_MOESM1_ESM.pdf]

Fig. S1. Study site locations in Sweden

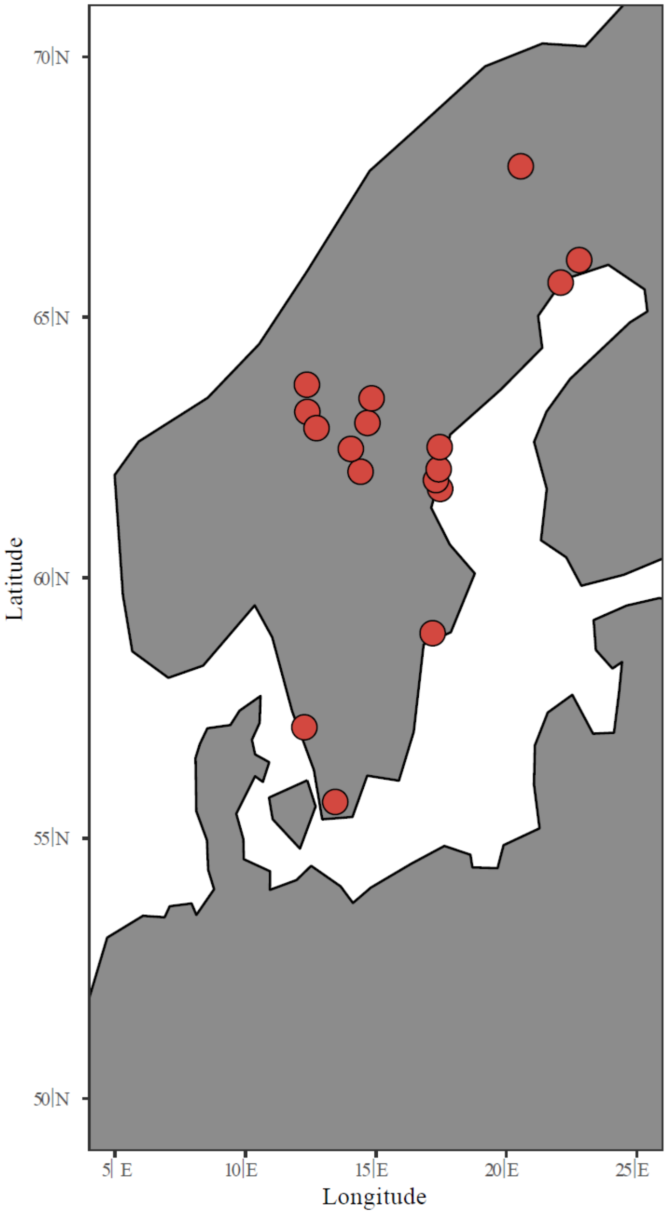

Locations of the 17 sites in Sweden where we deployed geolocators.

Fig. S2. Frequency distribution of the TE copy numbers that is used to infer presence or absence of MARB-a.

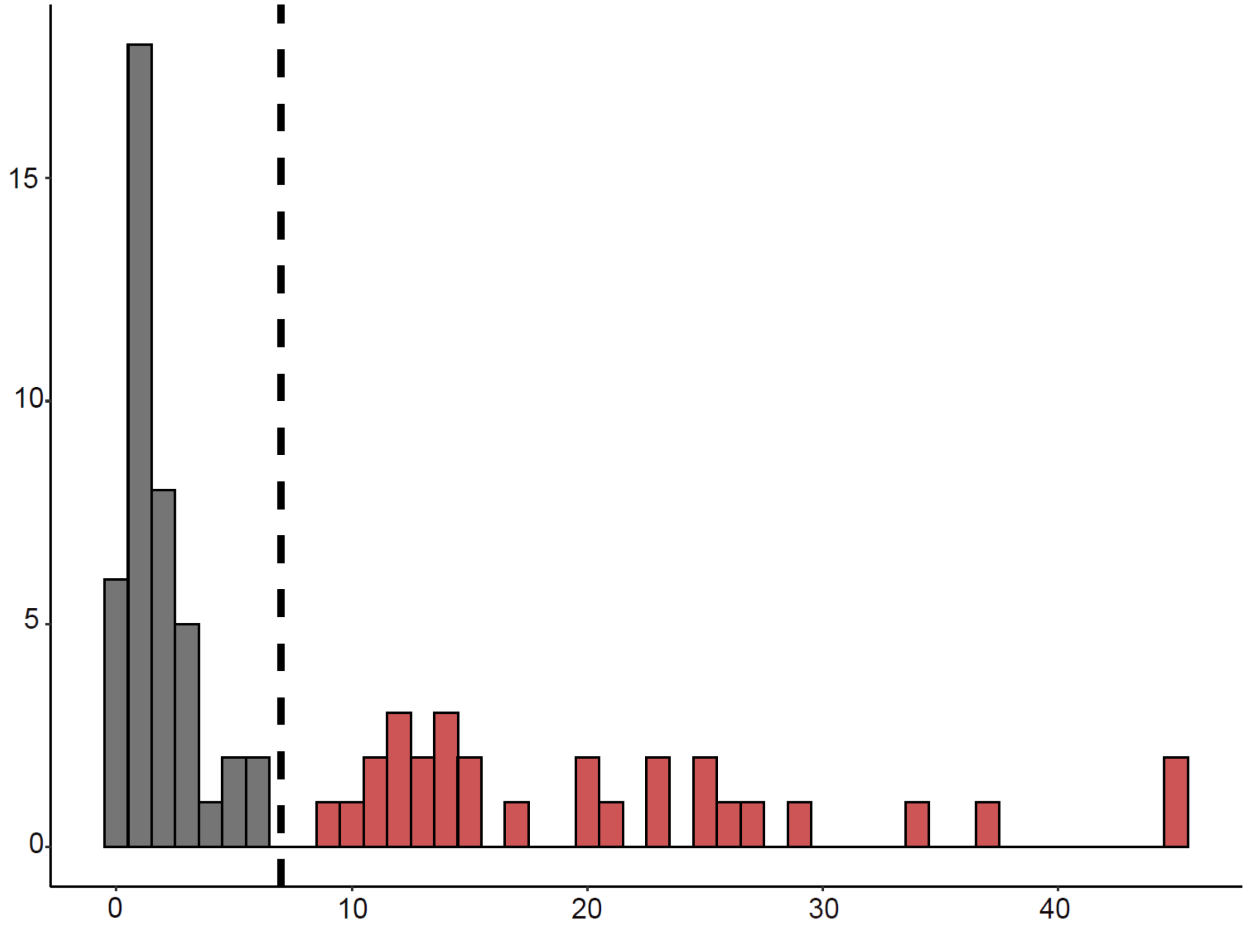

Histogram showing frequency distribution of the absolute number of transposon copies (X-axis) estimated with qPCR. The dashed line at 7 shows (our chosen) threshold value that sets apart presence or absence of MARB-a as in Caballero-Lopez et al. (2022)[1]. This figure includes all the 72 birds we obtained tracking data from. Grey bars represent individuals that are homozygous for the absence of MARB-a, the red bars those that are heterozygous or homozygous for the presence of MARB-a.

Fig. S3. Wintering locations of tracked with major winter range groups color shaded.

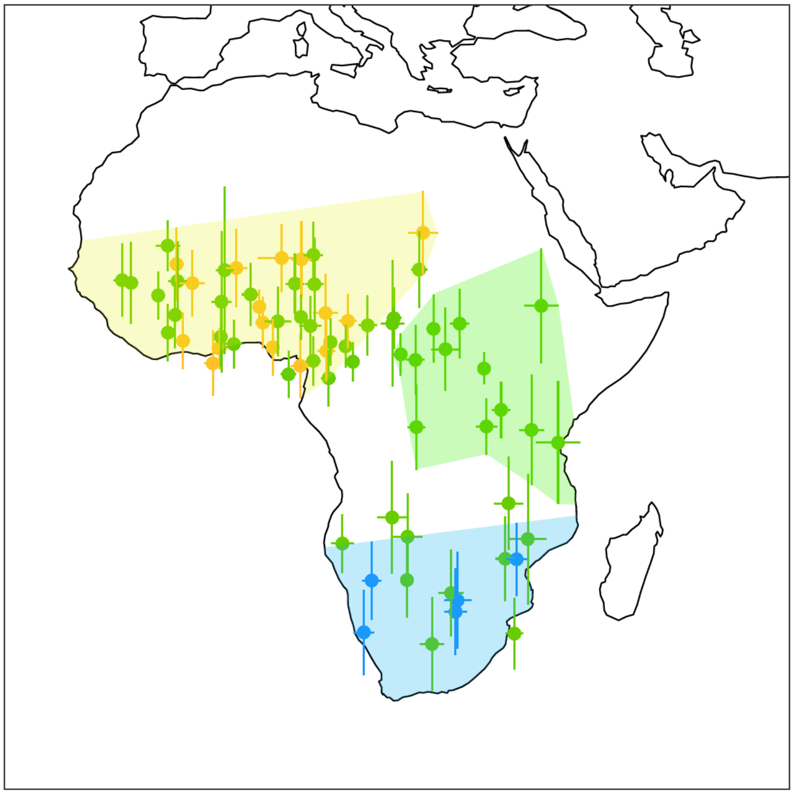

Winter sites of allopatric *trochilus* from southern Sweden in orange (n=16), allopatric *acredula* from northern Sweden in blue (n =5) and birds from the migratory divide in green (n=51). Error bars show standard deviations. Shaded polygons illustrate the winter range of the respective groups following the limits of standard deviations in longitude and latitude of the main winter sites. Polygon lines for *trochilus* and *acredula* are extended to connect to continent borders. Birds from the migratory divide mostly wintered in the winter ranges of either of allopatric subspecies (29 in *trochilus* range, 10 in *acredula* range), with only 12 wintering in between. The birds for which the standard deviation of latitude extends into one of allopatric subspecies ranges are considered as wintering within that subspecies. Error bars show standard deviations in longitude and latitude of the main winter site of each bird.

Fig. S4. Autumn migration routes in relation to genotypes on InvP-Ch1 and InvP-Ch5.

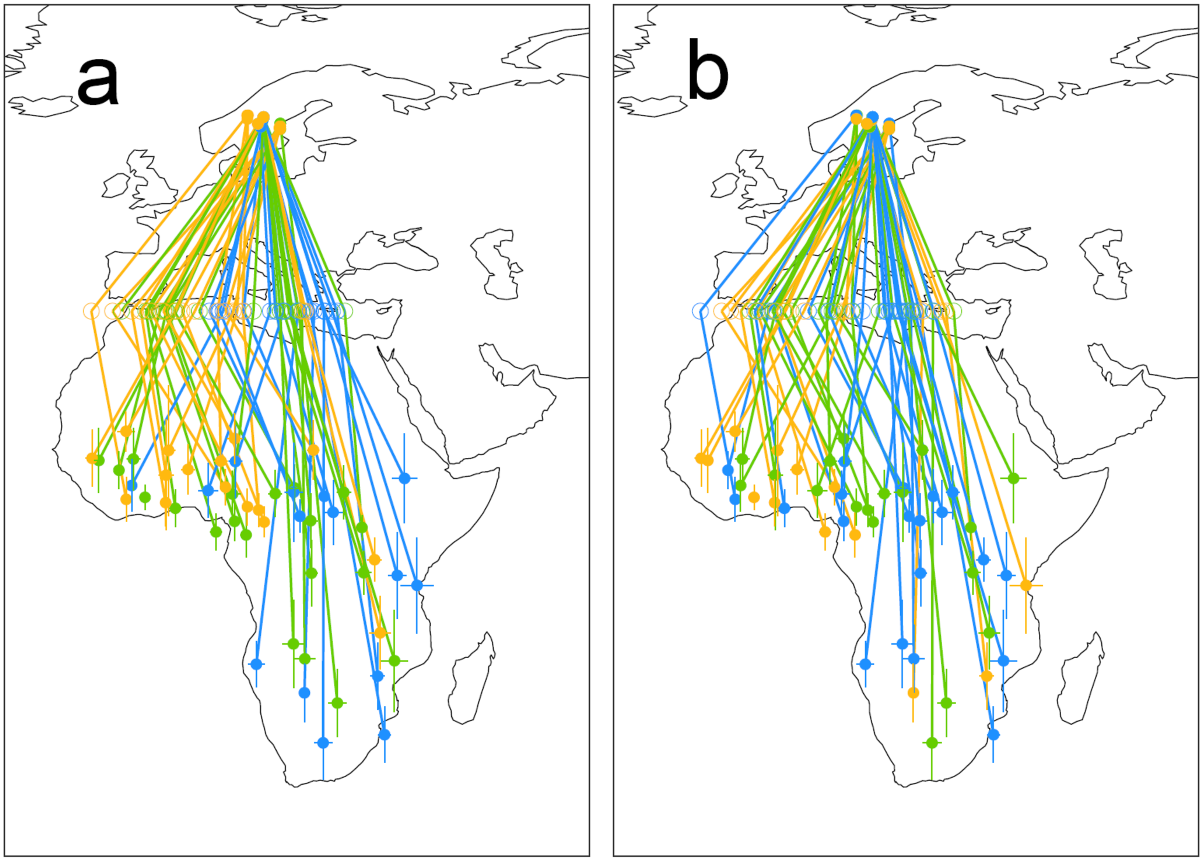

Breeding and wintering areas of willow warblers tracked from the Swedish migratory divide. The color codes correspond to the genotypes of invP-Ch1 (a, n = 51) and invP-Ch5 (b, n = 51): blue and orange are homozygotes for *acredula* and *trochilus* alleles respectively. Green denotes heterozygote genotypes. Error bars show standard deviations in coordinates for the main winter site. Error bars show standard deviations for latitude and longitude coordinates for the main winter site. Hollow circles mark the estimated Mediterranean crossing longitude at latitude 35 °N.

Fig. S5. Circular plot of autumn migration direction of birds from the Swedish migratory divide.

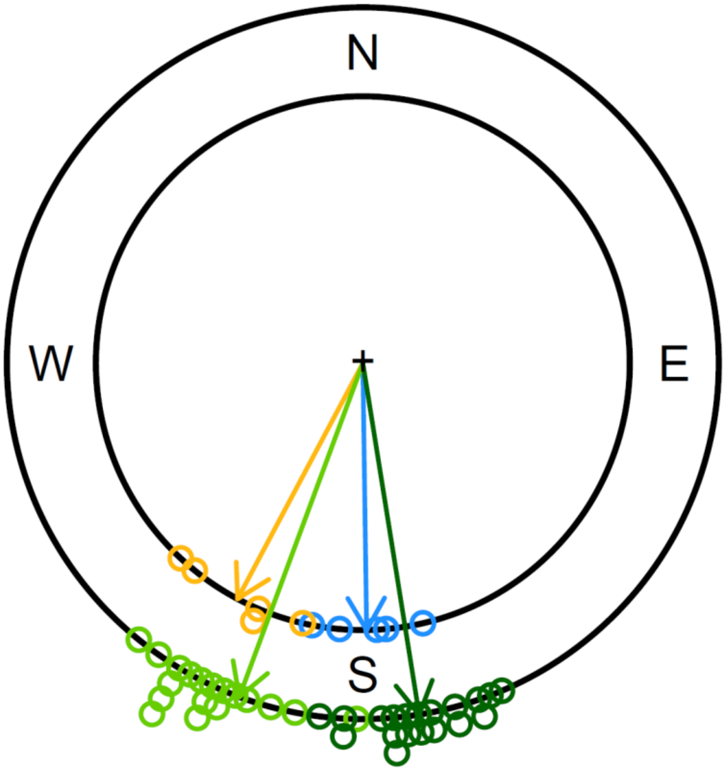

Autumn migration direction from the breeding site to the site of the Mediterranean crossing of willow warblers from the Swedish migratory divide. Orange shows genetic *trochilus* (n = 5) and blue, genetic *acredula* (n = 5). Light green are birds with hybrid genotypes that do not have MARB-a (n = 21) and dark green are hybrids that do have MARB-a (n = 19). Arrows indicate corresponding circular means.

Table S1. Complete output of generalized linear models reported in the study.

Model: Direction ~ MARB-a + InvP-Ch1 + InvP-Ch5; Adjusted R^2: 0.6756; DF: 45; p-value: 0.00000000004972

	**SS**	**DF**	**F**	**P**
MARB-a	7759.6	1	67.1915	0.0000000001784
InvP-Ch1	1204.9	2	5.2167	0.009171
InvP-Ch5	249.8	2	1.0817	0.347673

Model: Direction ~ MARB-a * InvP-Ch1; Adjusted R^2: 0.7416; DF: 45; p-value: 0.0000000000003342

	**SS**	**DF**	**F**	**P**
MARB-a	6513	1	70.8015	0.00000000008665
InvP-Ch1	2396	2	13.0232	0.00003448137608
MARB-a * InvP-Ch1	1307	2	7.1025	0.002086

Model: Winter longitude ~ MARB-a + InvP-Ch1 + InvP-Ch5; Adjusted R^2: 0.5647; DF: 45; p-value: 0.00000003002

	**SS**	**DF**	**F**	**P**
MARB-a	3618.763	1	43.08982	0.0000000452
InvP-Ch1	296.0816	2	1.762772	0.183209
InvP-Ch5	423.501	2	2.521384	0.091643

Model: Distance ~ InvP-Ch1 + InvP-Ch5; Adjusted R^2: -0.131; DF: 16; p-value: 0.7936

	**SS**	**DF**	**F**	**P**
InvP-Ch1	1.29E+11	2	0.032489	0.968097
InvP-Ch5	3.25E+12	2	0.819361	0.458377

Model: Autumn departure ~ MARB-a + InvP-Ch1 + InvP-Ch5; Adjusted R^2: -0.08024; DF: 45; p-value: 0.9339

	**SS**	**DF**	**F**	**P**
MARB-a	12.98398	1	0.463029	0.499698
InvP-Ch1	27.67137	2	0.493403	0.61381
InvP-Ch5	2.301222	2	0.041033	0.959834

SS- sum of squares, DF – degrees of freedom. We reported Type II ANOVA for models without interactions and type III ANOVA results for models with an interaction term.

Table S2. Pairwise Watson's U^2 tests of autumn migration direction.

	Allopatric *acredula*	Allopatric *trochilus*	Genetic *acredula* from sympatry	Genetic *trochilus* from sympatry	Hybrids without MARB-a	Hybrids with MARB-a
Allopatric *acredula*	X					
Allopatric *trochilus*	**0.32 ****	X				
Genetic *acredula* from sympatry	0.04^{ns}	**0.32****	X			
Genetic *trochilus* from sympatry	**0.22***	0.07^{ns}	**0.22***	X		
Hybrids without MARB-a	**0.20***	0.09^{ns}	**0.19***	0.07^{ns}	X	
Hybrids with MARB-a	0.04^{ns}	**0.72*****	0.07^{ns}	**0.33****	**0.56*****	X

Two-sided Watson's U^2 statistic for pairwise comparison of autumn migration directions between allopatric/sympatric *acredula*/*trochilus* and hybrids with and without MARB-a. Results have not been corrected for multiple testing. All tests carried out with Alpha = 0.05. Significance levels: ns $P > 0.5$, * $P \leq 0.05$, ** $P \leq 0.01$, *** $P \leq 0.001$. Values in bold alongside with stars indicate pairwise comparisons with significant differences.

Table S3. Comparison of circular and conventional mean values.

	Circular mean	Rayleigh's p	Rayleigh's test statistic	Cconventional mean	n
allopatric *acredula*	177.9	0.0014	0.9916	177.9	5
allopatric *trochilus*	207.9	0	0.9594	207.9	16
genetic *acredula* from sympatry	179.2	0.0015	0.9894	179.2	5
genetic *trochilus* from sympatry	207.9	0.0018	0.9809	207.9	5
hybrids without MARB-a	201.0	0	0.9729	201.0	22
hybrids with MARB-a	170.8	0	0.9901	170.8	19

Rayleigh's test of uniformity and comparison of circular and conventional means of autumn departure angles of willow warbler subgroups according to subspecies and presence or absence of MARB-a.

Table S4. Primers and probes used for the qPCR genotyping.

	**SNP 65 on InvP-Ch1**	**SNP 285 on InvP-Ch5**
**Forward primer**	TGAAACAGAATTTTGCAGTACTTCTACCT	CATCTGGGCTCCACTTGCT
**Reverse primer**	GTGCTTTCTAGTATATGGAAAAAAGAGGGA	TGTTGCCTAAATCCTGGGTTTCC
**VIC probe (*acredula*)**	ATTGAAATCAA**G**AATTCAG	TGCCC**G**GATTTTT
**FAM probe (*trochilus*)**	ATTGAAATCAA**T**AATTCAG	TGCCC**A**GATTTTT

The details of the qPCR assays used to genotype InvP-Ch1 and InvP-ch5.

Table S5. Inheritance pattern of TE copy number.

Family 1	TE copy number	Inferred Genotype	Family 2	TE copy number	Inferred Genotype
Father	27	+/+	Father	15	—/+
Mother	1	—/—	Mother	2	—/—
Young1	10	—/+	Young1	0	—/—
Young2	14	—/+	Young2	1	—/—
Young3	11	—/+	Young3	15	—/+
Young4	14	—/+	Young4	1	—/—
Young5	10	—/+	Young5	1	—/—
			Young6	17	—/+
			Young7	13	—/+

The inheritance of TE copy number estimated by qPCR in two families of willow warblers sampled at the center of the migratory divide (63.5°N 14.8°E) in 2021. The paternity and maternity of the chicks were confirmed by genotyping of three highly polymorphic microsatellite loci [Ase60 (16 alleles, 60 C annealing temperature), Ase18 (23 alleles, 60 C annealing temperature) and MCYU4 (17 alleles, 53 C annealing temperature] with standard PCR method[2]. In family 1, the mother is homozygote for the absence of MARB-a (—/—) and the father inferred as being MARB-a homozygote (+/+) because all the five offspring have intermediate copy numbers as expected if these are heterozygote (—/+). In family 2, the mother is homozygote for the absence of MARB-a (—/—) and the father inferred as being MARB-a heterozygote (—/+) because the copy number in the offspring either resemble the father (—/+) or the mother (—/—).

References

1. Caballero-Lopez, V. Genetics of migration: detection and qPCR-based quantification of transposable elements linked to migratory phenotypes in the willow warbler (Phylloscopus trochilus). (2019).
2. Bensch, S., Grahn, M., Muller, N., Gay, L. & Åkesson, S. Genetic, morphological, and feather isotope variation of migratory willow warblers show gradual divergence in a ring. *Molecular Ecology* **18**, 3087–3096 (2009).
