# Supplementary material for: Migration direction in a songbird explained by two loci
Source: Nat Commun. 2023 Jan 11;14:165. doi: 10.1038/s41467-023-35788-7 (PMC9834303; doi:10.1038/s41467-023-35788-7)
Supplement: Supplementary file 3 — Reporting Summary [file 41467_2023_35788_MOESM3_ESM.pdf]

nature portfolio

Corresponding author(s): Kristaps Sokolovskis

Last updated by author(s): Nov 29, 2022

# Reporting Summary

Nature Portfolio wishes to improve the reproducibility of the work that we publish. This form provides structure for consistency and transparency in reporting. For further information on Nature Portfolio policies, see our Editorial Policies and the Editorial Policy Checklist.

## Statistics

For all statistical analyses, confirm that the following items are present in the figure legend, table legend, main text, or Methods section.

| n/a | Confirmed | |
|---|---|---|
| ☐ | ☒ | The exact sample size (*n*) for each experimental group/condition, given as a discrete number and unit of measurement |
| ☐ | ☒ | A statement on whether measurements were taken from distinct samples or whether the same sample was measured repeatedly |
| ☐ | ☒ | The statistical test(s) used AND whether they are one- or two-sided<br>*Only common tests should be described solely by name; describe more complex techniques in the Methods section.* |
| ☐ | ☒ | A description of all covariates tested |
| ☐ | ☒ | A description of any assumptions or corrections, such as tests of normality and adjustment for multiple comparisons |
| ☐ | ☒ | A full description of the statistical parameters including central tendency (e.g. means) or other basic estimates (e.g. regression coefficient) AND variation (e.g. standard deviation) or associated estimates of uncertainty (e.g. confidence intervals) |
| ☐ | ☒ | For null hypothesis testing, the test statistic (e.g. *F*, *t*, *r*) with confidence intervals, effect sizes, degrees of freedom and *P* value noted<br>*Give P values as exact values whenever suitable.* |
| ☒ | ☐ | For Bayesian analysis, information on the choice of priors and Markov chain Monte Carlo settings |
| ☒ | ☐ | For hierarchical and complex designs, identification of the appropriate level for tests and full reporting of outcomes |
| ☒ | ☐ | Estimates of effect sizes (e.g. Cohen's *d*, Pearson's *r*), indicating how they were calculated |

*Our web collection on statistics for biologists contains articles on many of the points above.*

## Software and code

Policy information about availability of computer code

| | |
|---|---|
| Data collection | Tracking and data collection was done by the authors of the study. No special sofware ore unique code was involved. |
| Data analysis | We used R version 4.1.1 (R Core Team 2021)<br>And the following packages: "GeoLight" v 2.0, “geosphere” v 1.5-10, "circular" v 0.4-93, "car" v 3.0-12, "ggplot2" v 3.3.6, "gstat" 2.0-8.<br>Circular plots in Fig. 3 were created with program: ORIANA (version4.02). Colours and arrangement of panels in figures were adjusted with Affinity designer (version 1.10.5). |

For manuscripts utilizing custom algorithms or software that are central to the research but not yet described in published literature, software must be made available to editors and reviewers. We strongly encourage code deposition in a community repository (e.g. GitHub). See the Nature Portfolio guidelines for submitting code & software for further information.

## Data

Policy information about availability of data

All manuscripts must include a data availability statement. This statement should provide the following information, where applicable:

- Accession codes, unique identifiers, or web links for publicly available datasets
- A description of any restrictions on data availability
- For clinical datasets or third party data, please ensure that the statement adheres to our policy

Source data are provided with this paper. The geolocator data generated in this study have been deposited in the Dryad database under open access. The data

generated in this study are provided in the Supplementary Information, Supplementary Data, and Source Data files. Link to raw geolocator data : https://datadryad.org/stash/share/tCEX5YTwodjkplmwPnPgX8FuVlKaYZxNVRoKgKguMWQ

## Human research participants

Policy information about studies involving human research participants and Sex and Gender in Research.

| | |
|---|---|
| Reporting on sex and gender | We did not study humans. |
| Population characteristics | We did not study humans. |
| Recruitment | We did not study humans. |
| Ethics oversight | We did not study humans. |

Note that full information on the approval of the study protocol must also be provided in the manuscript.

# Field-specific reporting

Please select the one below that is the best fit for your research. If you are not sure, read the appropriate sections before making your selection.

☐ Life sciences ☐ Behavioural & social sciences ☒ Ecological, evolutionary & environmental sciences

For a reference copy of the document with all sections, see nature.com/documents/nr-reporting-summary-flat.pdf

# Ecological, evolutionary & environmental sciences study design

All studies must disclose on these points even when the disclosure is negative.

| | |
|---|---|
| Study description | We tracked wild birds from breeding grounds in Sweden to winter grounds in tropical Africa. We genotyped all tracked birds on three genetic markers we had singled out as potential loci affecting migratory behaviour. |
| Research sample | We deployed a total of 466 Migrate Technology Ltd geolocators (Intigeo-W30Z11-DIP 12x5x4mm, 0.32 g) and recaptured a total of 73, of which one logger malfunctioned thus had a final data set of 72 migration tracks and DNA samples of all these birds. We chose to deploy 466 units because that is how many we could afford. From those we deployed 60 in northern Sweden and 50 in southern Sweden to get meaningful number of reference tracks of alopatric acredula and trochilus. We deployed more in northern Sweden because return rates of northern populations are smaller and more difficult to predict (doi: 10.1111/jav.00973). Locations were we tagged trochilus in southern Sweden: 55.69°N 13.44°E, 58.93°N 17.16°E, 57.09°N 12.29°E. Locations in northern Sweden: 66.09°N 22.78°E, 65.66°N 22.07°E, 67.88°N, 20.44°E). Remaining 356 geolocators were deployed in the migratory divide in Sweden between latitudes 62°N and 64°N.<br>We retrieved total of 73 loggers. One malfunctioned so we had 72 tracks in total. %Five of these tracks were from norhtern Sweden (acredula) and 16 of trochilus from southern Sweden. Remaining 51 tracks were form migratory divide in central Sweden. Upon recapture we collected blood samples of each of the tracked birds for DNA analyzis.<br><br>We tracked only adult males, i.e. birds that were in their 2nd calendar year or older. It is not possible to age willow warblers more precisely than that. |
| Sampling strategy | We were primarily interested in migratory phenotypes and how the associate with various genotypes in birds from a migratory divide. Since none of the hybrid genotypes in willow warblers can be identified by morphometrics we deployed as many geolocators as we could afford to increase the chance of tagging as many interesting genotypes as possible. |
| Data collection | Birds were captured from late May to late June (from 2018 to 2021) in their breeding territories using mistnets and playback of a song. Geolocators were deployed with leg loop harness and blood was collected with a syringe from brachial vein. |
| Timing and spatial scale | As a first step we purchased 349 loggers of which we deployed 60 in northern Sweden (between lat 65 and 68) 50 in southern Sweden (between Lat 55 and 58) and 239 in the migratory divide in central Sweden (between lat 61 and 64). In 2019 we retreived 16 in soputhern Sweden, 5 in northern Sweden and 36 in central Sweden. We carried out analyzis of genotype association and migratory direction on these samples. However the sample size from the migratory divide was too small to draw robust conlcusions. Therefore in 2020 we purchased 117 more geolocators (as many as we could afford at that point in time) and deployed them in one single site in migratory divide in central Sweden where we knew from previosu work that there are many hybrids breeding. In 2021 we retreived ata from 16 more birds from migratoru divide. This was great addition and this boost in samples size allowed us to draw solid conclusions. Work was carried out from mid may to late June of each respective year. |
| Data exclusions | No data was excluded from the analyzis. |
| Reproducibility | All the resutls we have reported can be reproduced with the data we provide laongside wioth this manuscript. We have reported exact locations of the study sites and used standard methods for processing geolocator data. All molecular work was carried out according to two protocols previously published and also described in this study. Raw geolocator data is uploaded and publicly accessible in Dryad https://datadryad.org/stash/share/tCEX5YTwodjkplmwPnPgX8FuVlKaYZxNVRoKgKguMWQ. Genotyping results |

| | |
|---|---|
| | are reported in Sourca data file. Blood samples andd extracted DNA sampels are store at -80 C freezer at Lund university and can be reanalyzed if necessery. Every step of this work can be reproduced. |
| Randomization | We deployed a subset of loggers in northern and southern Sweden where pure allopatric subspecies that migrate SE and SW respectively breed to obtain reference data on pure subspecies. The remaining loggers were randomly distributed on willow warblers in the migratory divide in central Sweden between latitudes 61 and 64 °N |
| Blinding | Not relevant to the present study because it is an observational study. |

Did the study involve field work? ☒ Yes ☐ No

## Field work, collection and transport

| | |
|---|---|
| Field conditions | All field work was carried out in willow warbler breeding habitats in Sweden during May-June of 2018, 19, 20 and 21. We never worked if i it was raining because capturing birds during rain is harmful to them. Weather conditions in Sweden in Spring/early summer are very variable. We chose to handle birds only when it was sufficianetly warm (above +10°C and not rainy). All field sites were accessed byt driving and walking. |
| Location | We carried out field work in breeding habitats of willow warblers: early succession forest edges and mountain birch forests. Locations were we tagged trochilus in southern Sweden: 55.69°N 13.44°E, 58.93°N 17.16°E, 57.09°N 12.29°E. Locations in northern Sweden: 66.09°N 22.78°E, 65.66°N 22.07°E, 67.88°N, 20.44°E. Rest of the 11 study sites were distributred in central Sweden between latitudes 61°N and 64°N. Three of the study sites in the migratory divide were placed near the treeline at 800 - 1000 m.a.s.l.. Study sites near coastlines had average elevation at about 70 m.a.s.l. |
| Access & import/export | We carried out all the work within Sweden so no special permits for transportation of samples were necessary. |
| Disturbance | Willow warblers are small passerines weighing about 10 grams on average. We used lightest available tracking devices. Current rule of thumb is to never attach anything to a bird that weighs more than 5% of its body weight. Our loggers constituted on average 3.3% (range 2.7 – 3.8%) of bird's body mass. In addition, we collected blood samples only from the birds that we recaptured in order to minimise the stress while deploying the logger. For willow warblers the return rate (a proxy for survival) does not differ measurably between birds tagged with loggers and birds simply colour-banded. |

# Reporting for specific materials, systems and methods

We require information from authors about some types of materials, experimental systems and methods used in many studies. Here, indicate whether each material, system or method listed is relevant to your study. If you are not sure if a list item applies to your research, read the appropriate section before selecting a response.

### Materials & experimental systems

| n/a | Involved in the study |
|---|---|
| ☒ | ☐ Antibodies |
| ☒ | ☐ Eukaryotic cell lines |
| ☒ | ☐ Palaeontology and archaeology |
| ☐ | ☒ Animals and other organisms |
| ☒ | ☐ Clinical data |
| ☒ | ☐ Dual use research of concern |

### Methods

| n/a | Involved in the study |
|---|---|
| ☒ | ☐ ChIP-seq |
| ☒ | ☐ Flow cytometry |
| ☒ | ☐ MRI-based neuroimaging |

## Animals and other research organisms

Policy information about studies involving animals; ARRIVE guidelines recommended for reporting animal research, and Sex and Gender in Research

| | |
|---|---|
| Laboratory animals | Study did not involve laboratory animals. |
| Wild animals | We captured adult male willow warblers (Phylloscopus trochilus) in their breeding territories, attached the loggers and released each bird back in their breeding territory. We returned the year after and recaptured the tagged birds that had returned, removed the logger, collected a blood sample from the brachial vein and released the bird back in their breeding territory. On each occasion total handling time of one bird was on average 15 minutes.<br><br>We only studied males because they are much easier to catch than females and have much higher retrurn rate to the previous breeding site. In addition males are on average slightly larger therefore geolocator mass will be proportionately less of the body mass in males than in females. It is impossible to score age of adult willow warblers. At the moment of trapping all we know is that its at |

| | |
|---|---|
| | least 2nd calendar summer for them. |
| Reporting on sex | In willow warblers as in most migratory passerines males show much higher breeding philopatry and are much easier to capture. Therefore we deployed geolocators only on males. |
| Field-collected samples | Blood samples collected in field were stored in SET buffer (0.015 M NaCl, 0.05 M Tris, 0.001 M of EDTA, pH 8.0) indoors at a room temperature, shielded from direct sunlight until deposited for permanent storage at -80°C freezer at Lund University. We did not carry out any experiemnts with collected blood samples, only extracvted DNA and using qPCR genotyped the birds. |
| Ethics oversight | Animals' care was in accordance with institutional guidelines. Ethical permit: Malmö-Lund djurförsöksetiska nämnd 5.8.18-00848/2018. |

Note that full information on the approval of the study protocol must also be provided in the manuscript.
