# Supplementary material for: Migration direction in a songbird explained by two loci
Source: Nat Commun. 2023 Jan 11;14:165. doi: 10.1038/s41467-023-35788-7 (PMC9834303; doi:10.1038/s41467-023-35788-7)
Supplement: Supplementary file 4 — Description of Additional Supplementary Files [file 41467_2023_35788_MOESM4_ESM.pdf]

Description of Additional Supplementary Files

File Name: Supplementary Data 1

Description:

Supplementary Data 1 (Separate pdf file)

Daily positions of each of the 72 tracked birds, with equinox periods excluded. Colour gradient denotes different months. The inherent geolocation noise has been removed with applying five day rolling mean for longitudes and latitude. Error bars show 5-day rolling standard deviations of longitude and latitude. Each map is labelled with the logger number that is cross referenced with Source Data file. The raw geolocator data is deposited in dryad DOI https://doi.org/10.5061/dryad.stqjq2c6t
