# Supplementary material for: Migration direction in a songbird explained by two loci
Source: Nat Commun. 2023 Jan 11;14:165. doi: 10.1038/s41467-023-35788-7 (PMC9834303; doi:10.1038/s41467-023-35788-7)
Supplement: Supplementary file 5 — Supplementary Data 1 [file 41467_2023_35788_MOESM5_ESM.pdf]

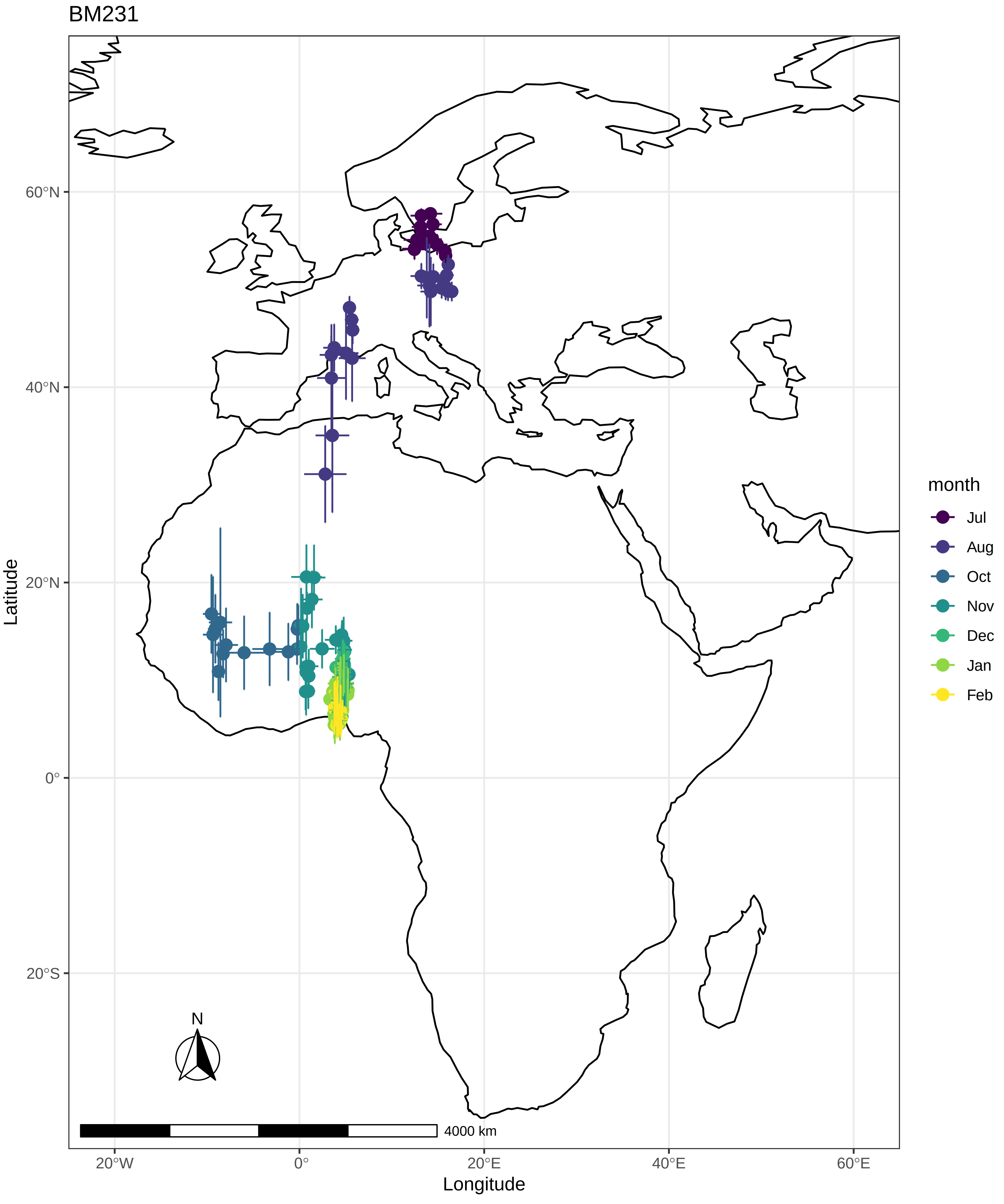
BM231
60°N
40°N
20°N
0°
20°S
Latitude
20°W
0°
20°E
40°E
60°E
Longitude
N
4000 km
month
Jul
Aug
Oct
Nov
Dec
Jan
Feb

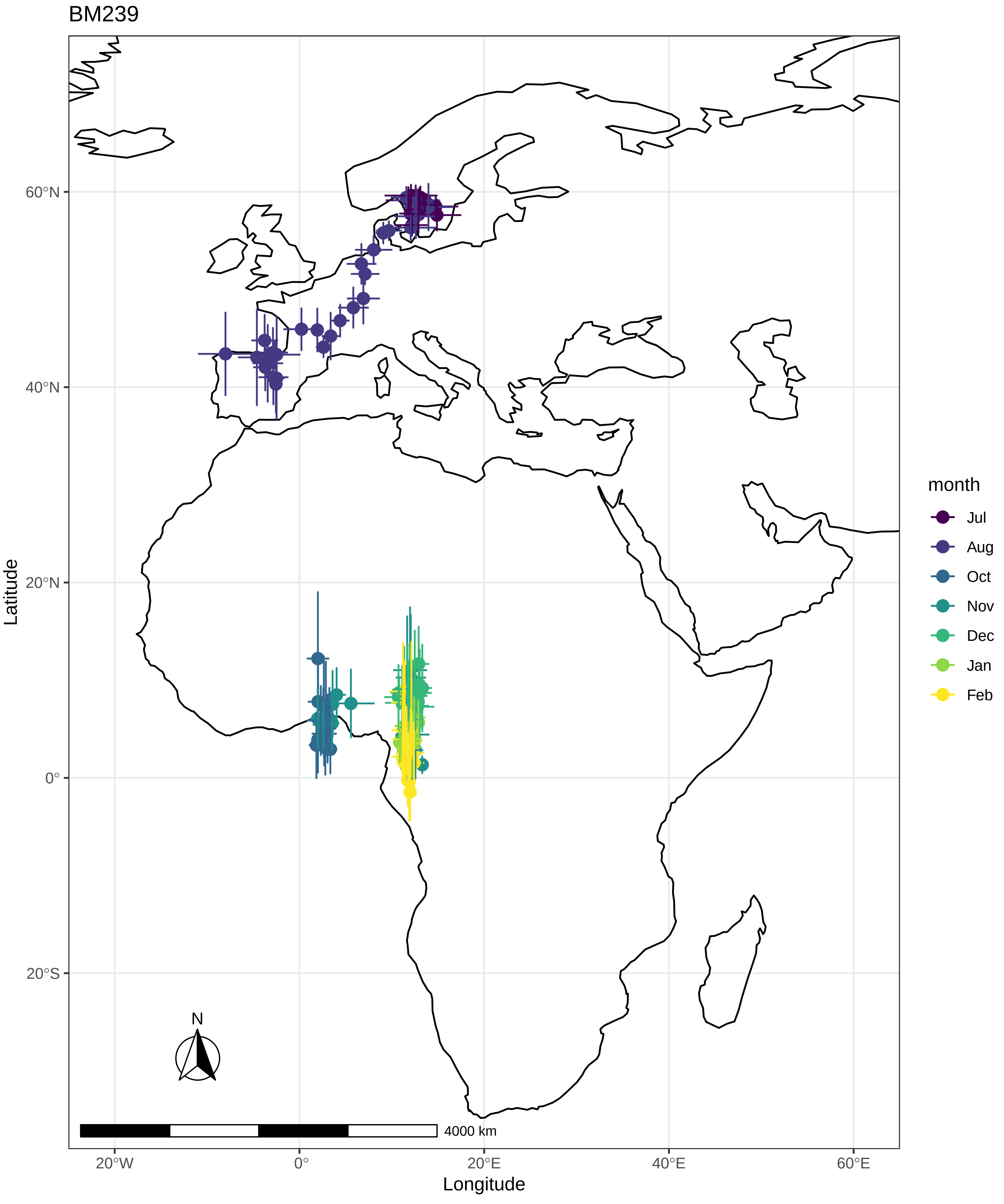
BM239
60°N
40°N
20°N
0°
20°S
20°W
0°
20°E
40°E
60°E
Latitude
Longitude
N
4000 km
month
Jul
Aug
Oct
Nov
Dec
Jan
Feb

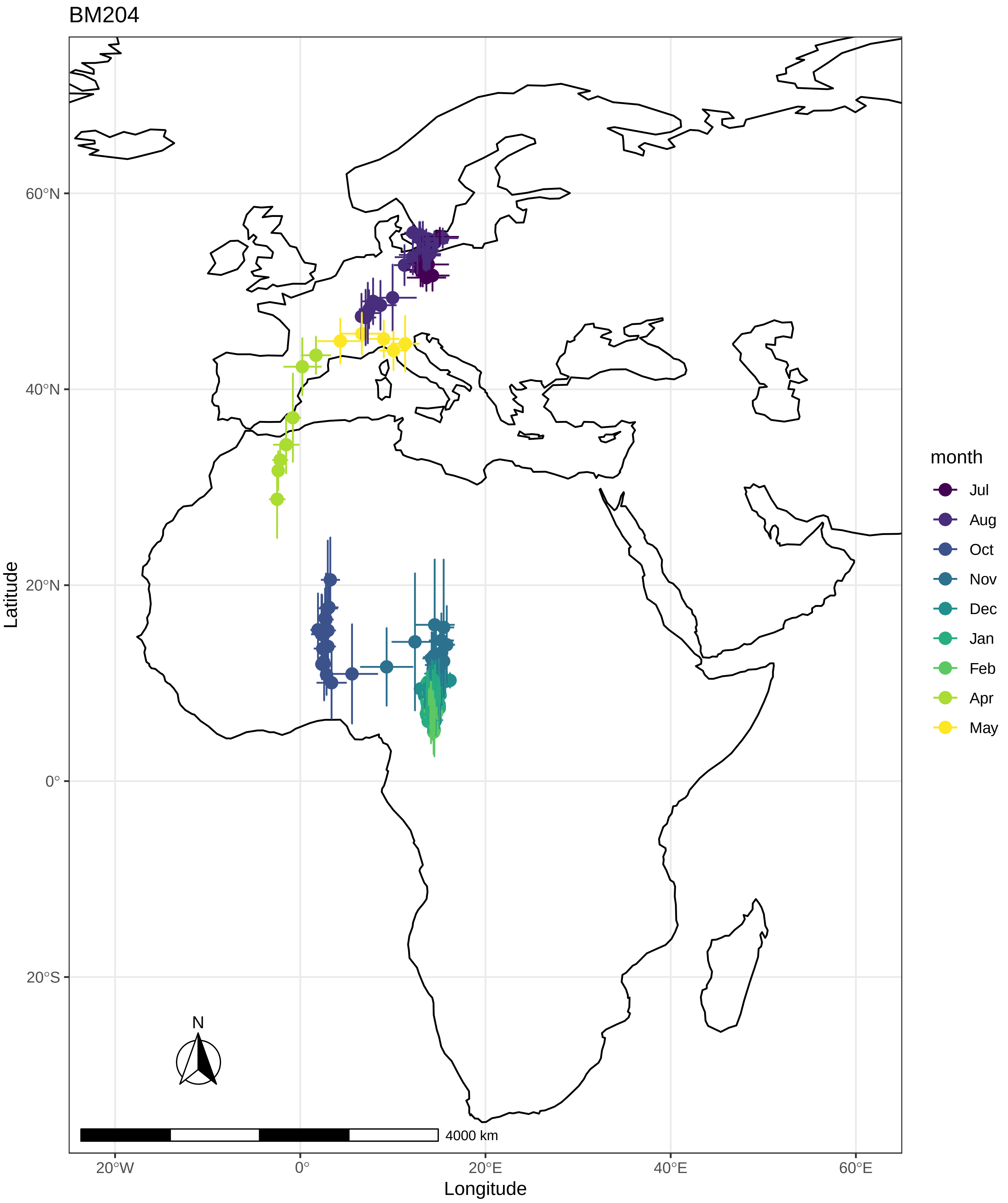

BM204
60°N
40°N
20°N
0°
20°S
Latitude
20°W
0°
20°E
40°E
60°E
Longitude
month
Jul
Aug
Oct
Nov
Dec
Jan
Feb
Apr
May
N
4000 km

BM214

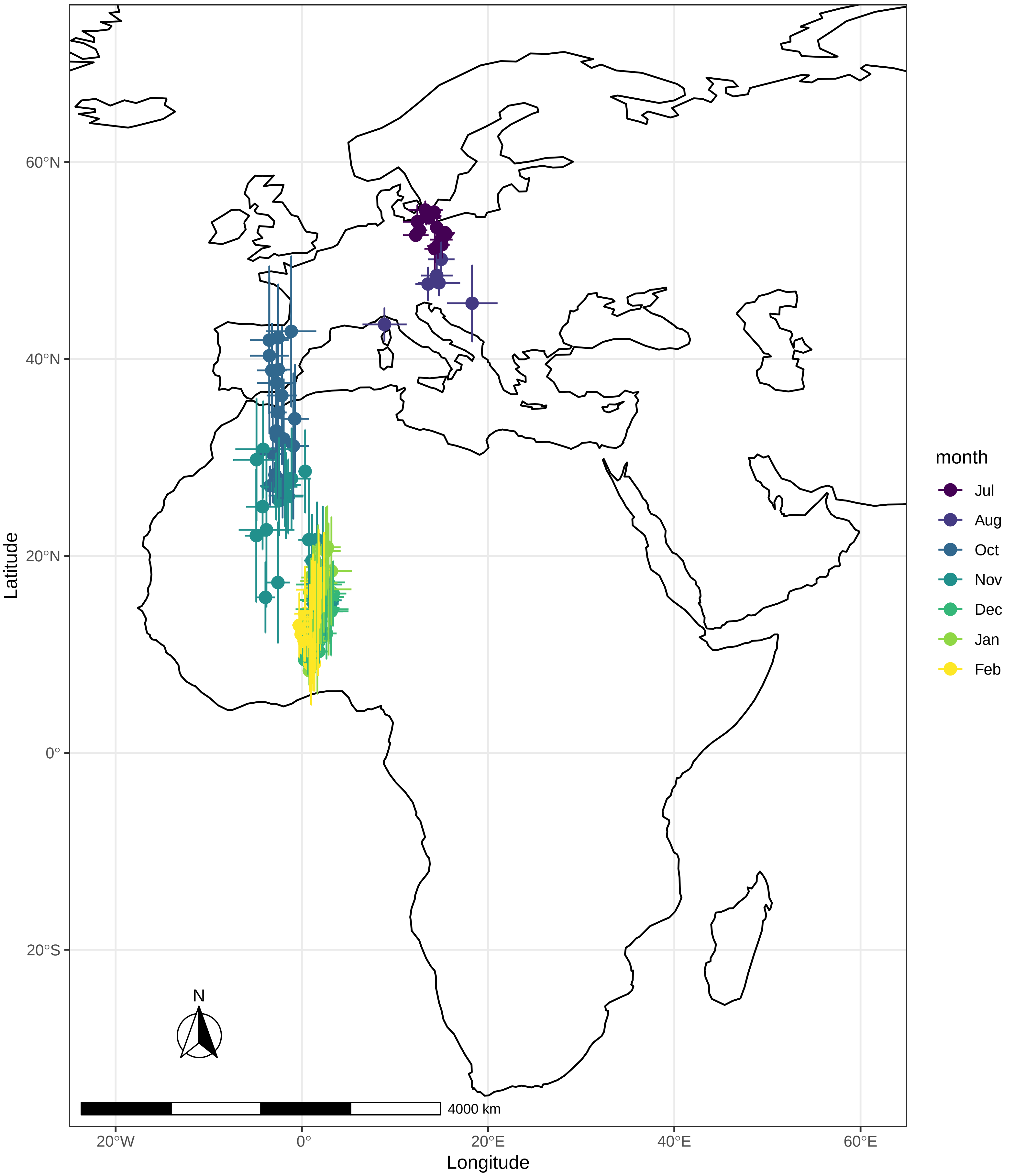

60°N
40°N
20°N
0°
20°S
20°W
0°
20°E
40°E
60°E
Latitude
Longitude
N
4000 km
month
Jul
Aug
Oct
Nov
Dec
Jan
Feb

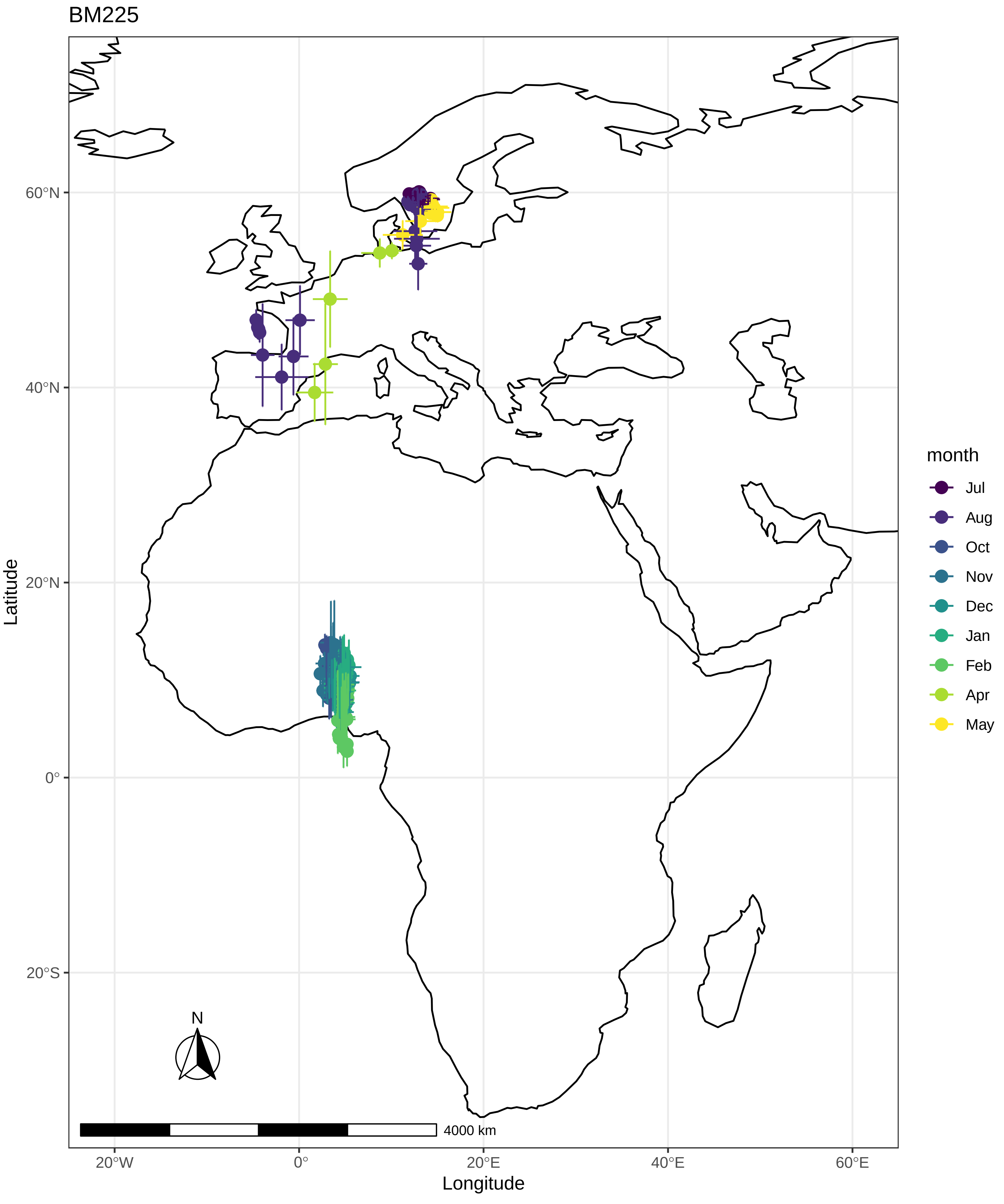

BM225
60°N
40°N
20°N
0°
20°S
Latitude
20°W
0°
20°E
40°E
60°E
Longitude
N
4000 km
month
Jul
Aug
Oct
Nov
Dec
Jan
Feb
Apr
May

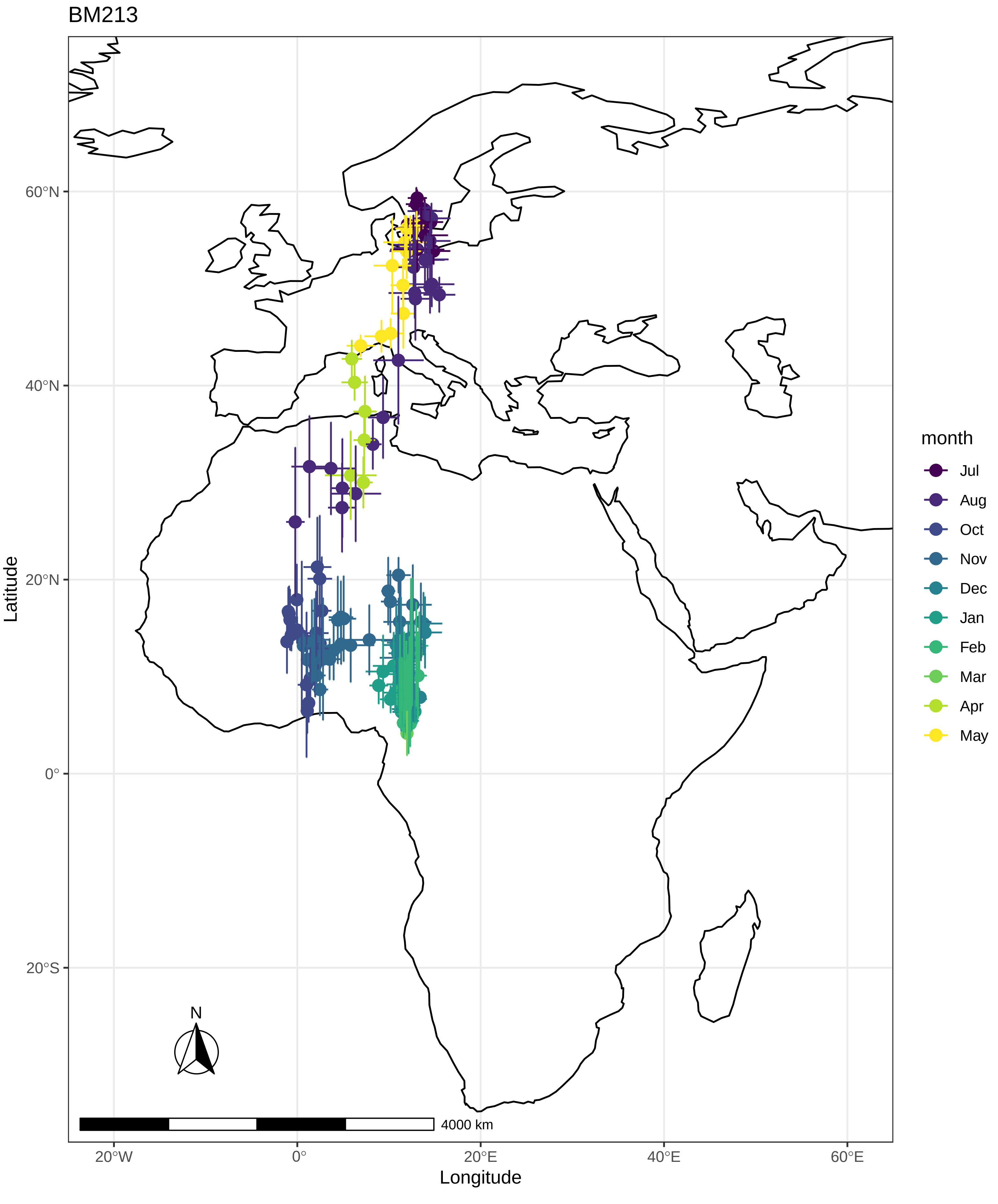

BM213
60°N
40°N
20°N
0°
20°S
20°W
0°
20°E
40°E
60°E
Latitude
Longitude
month
Jul
Aug
Oct
Nov
Dec
Jan
Feb
Mar
Apr
May
N
4000 km

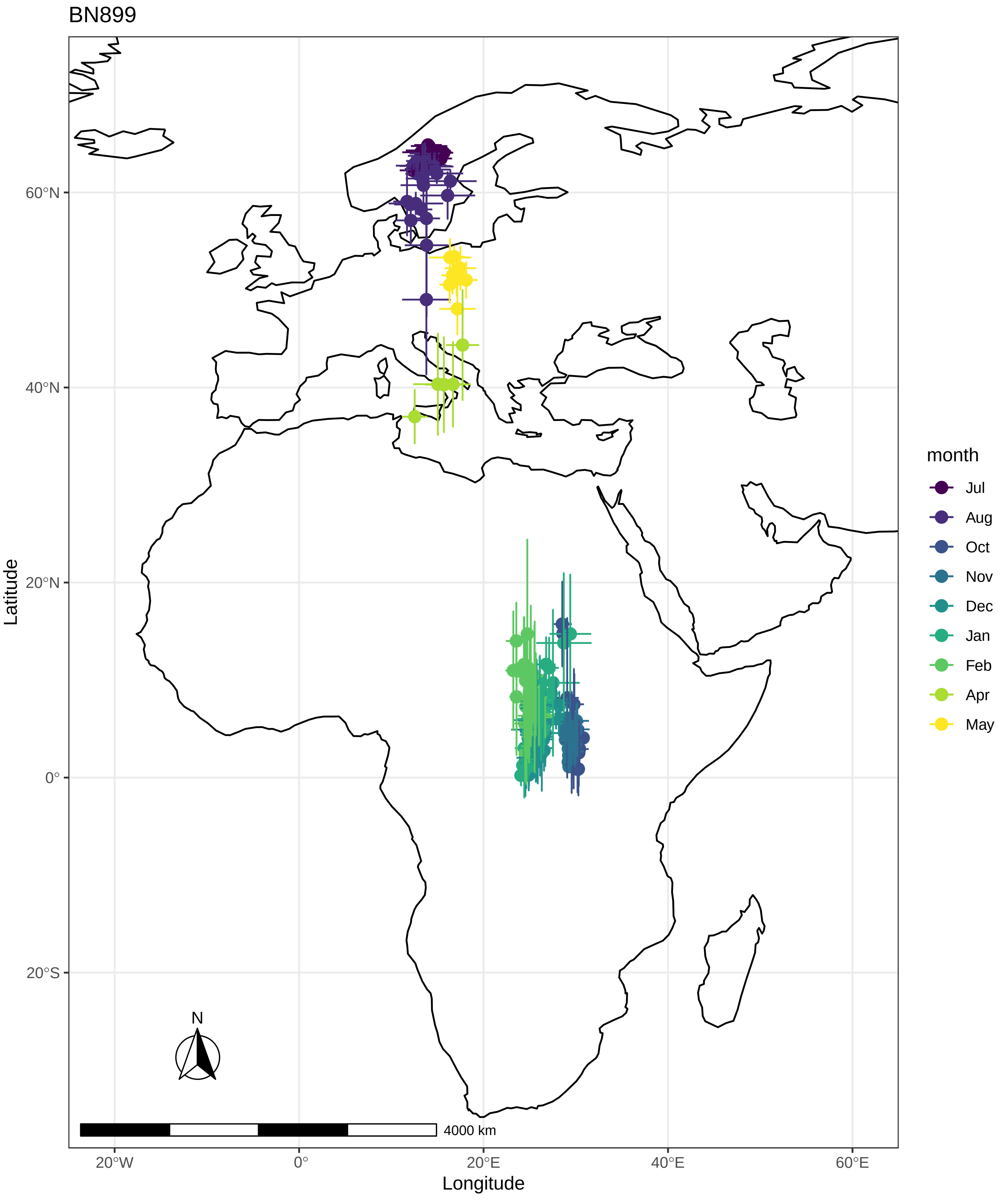

BN899
60°N
40°N
20°N
0°
20°S
Latitude
20°W
0°
20°E
40°E
60°E
Longitude
N
4000 km
month
Jul
Aug
Oct
Nov
Dec
Jan
Feb
Apr
May

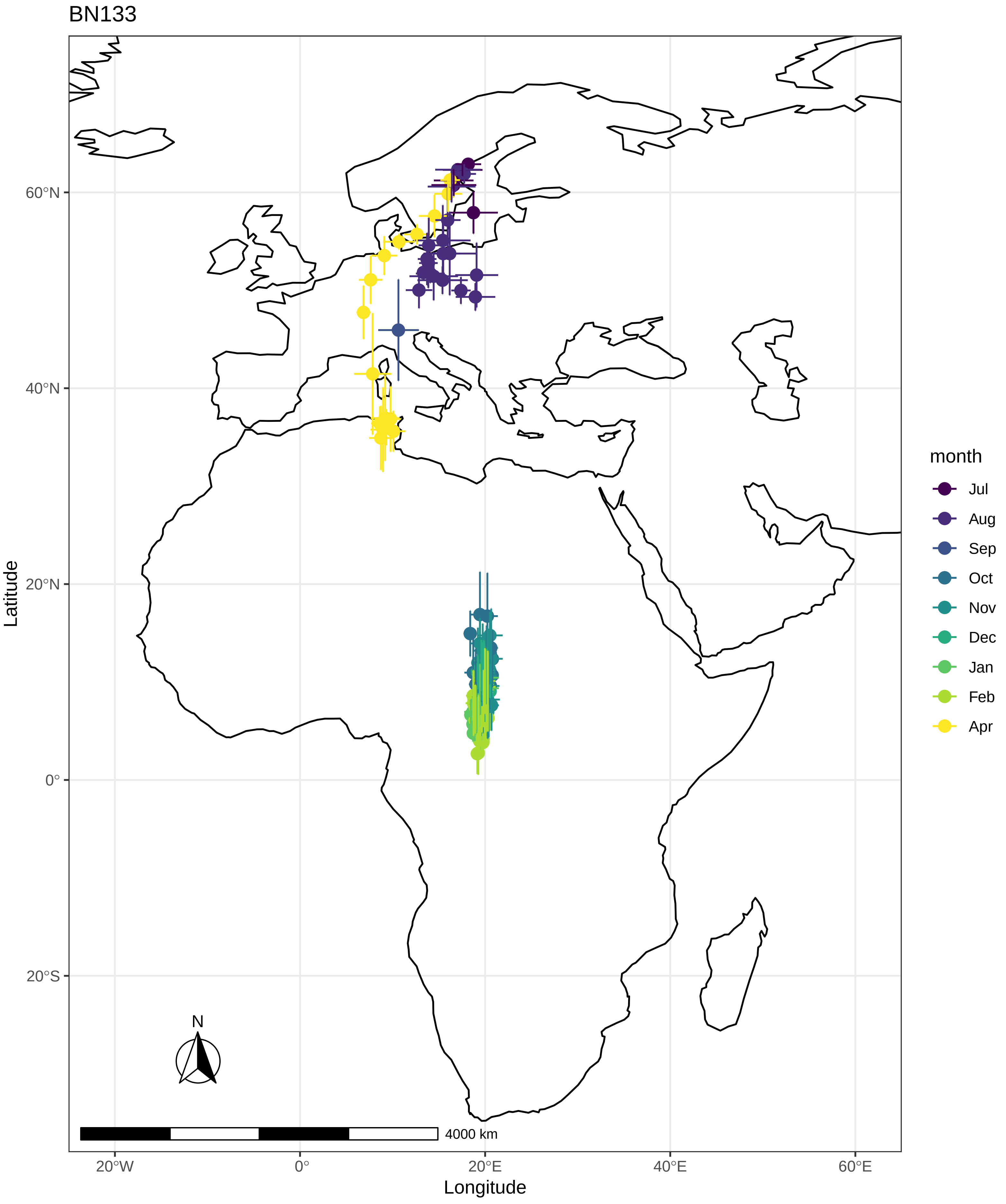

BN133
60°N
40°N
20°N
0°
20°S
20°W
0°
20°E
40°E
60°E
Latitude
Longitude
month
Jul
Aug
Sep
Oct
Nov
Dec
Jan
Feb
Apr
N
4000 km

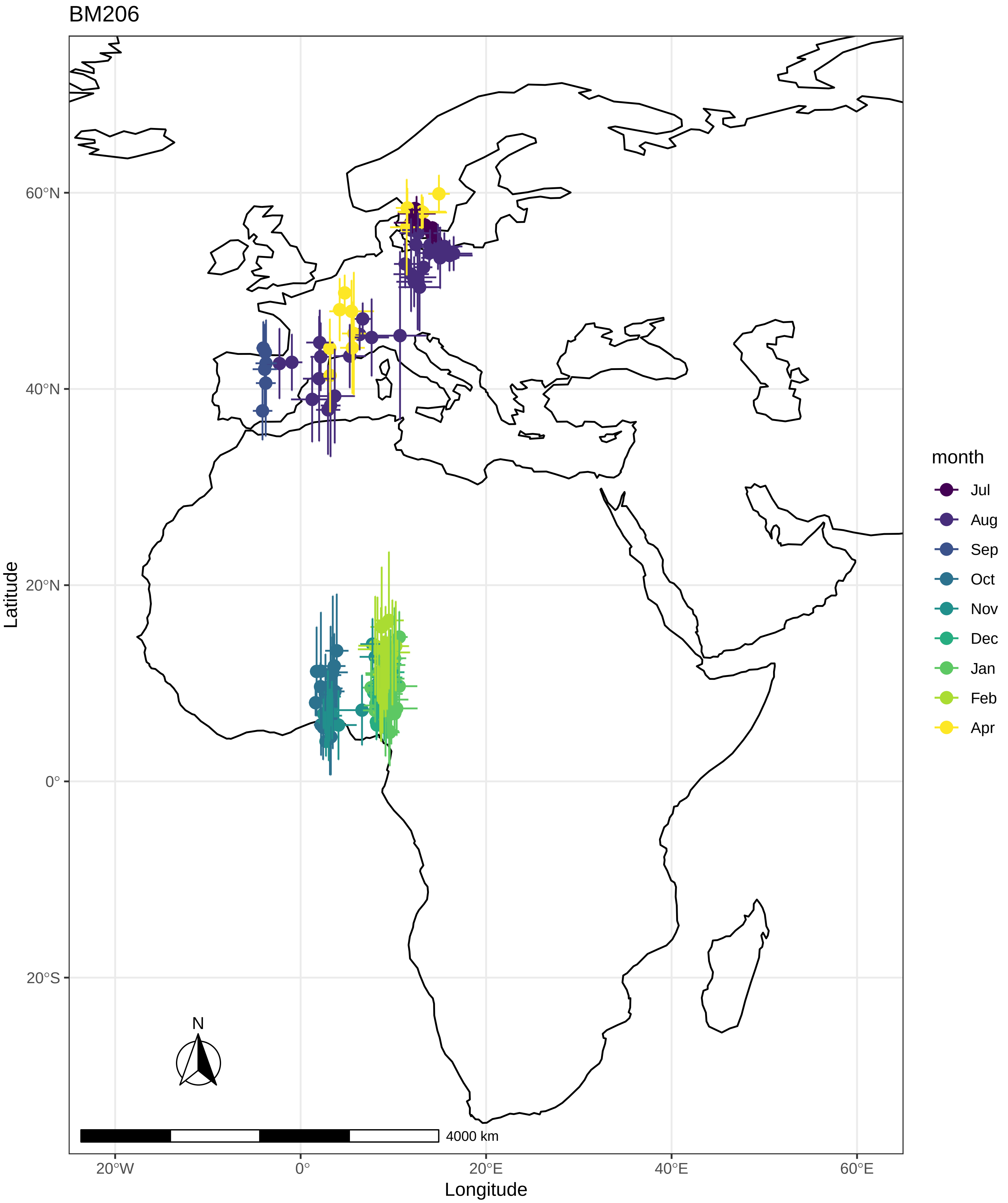
BM206
60°N
40°N
20°N
0°
20°S
20°W
0°
20°E
40°E
60°E
Latitude
Longitude
N
4000 km
month
Jul
Aug
Sep
Oct
Nov
Dec
Jan
Feb
Apr

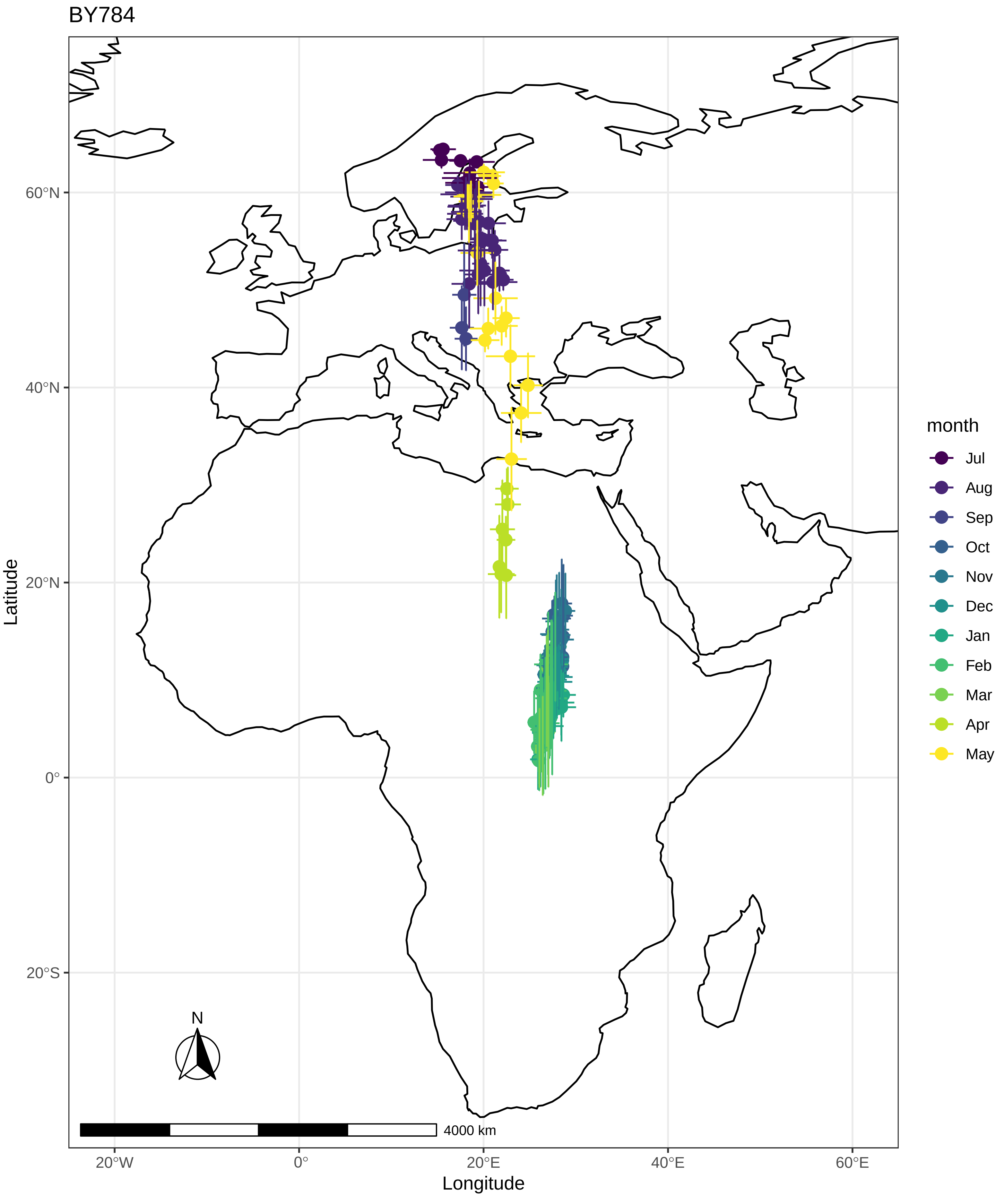

BY784
60°N
40°N
20°N
0°
20°S
Latitude
20°W
0°
20°E
40°E
60°E
Longitude
N
4000 km
month
Jul
Aug
Sep
Oct
Nov
Dec
Jan
Feb
Mar
Apr
May

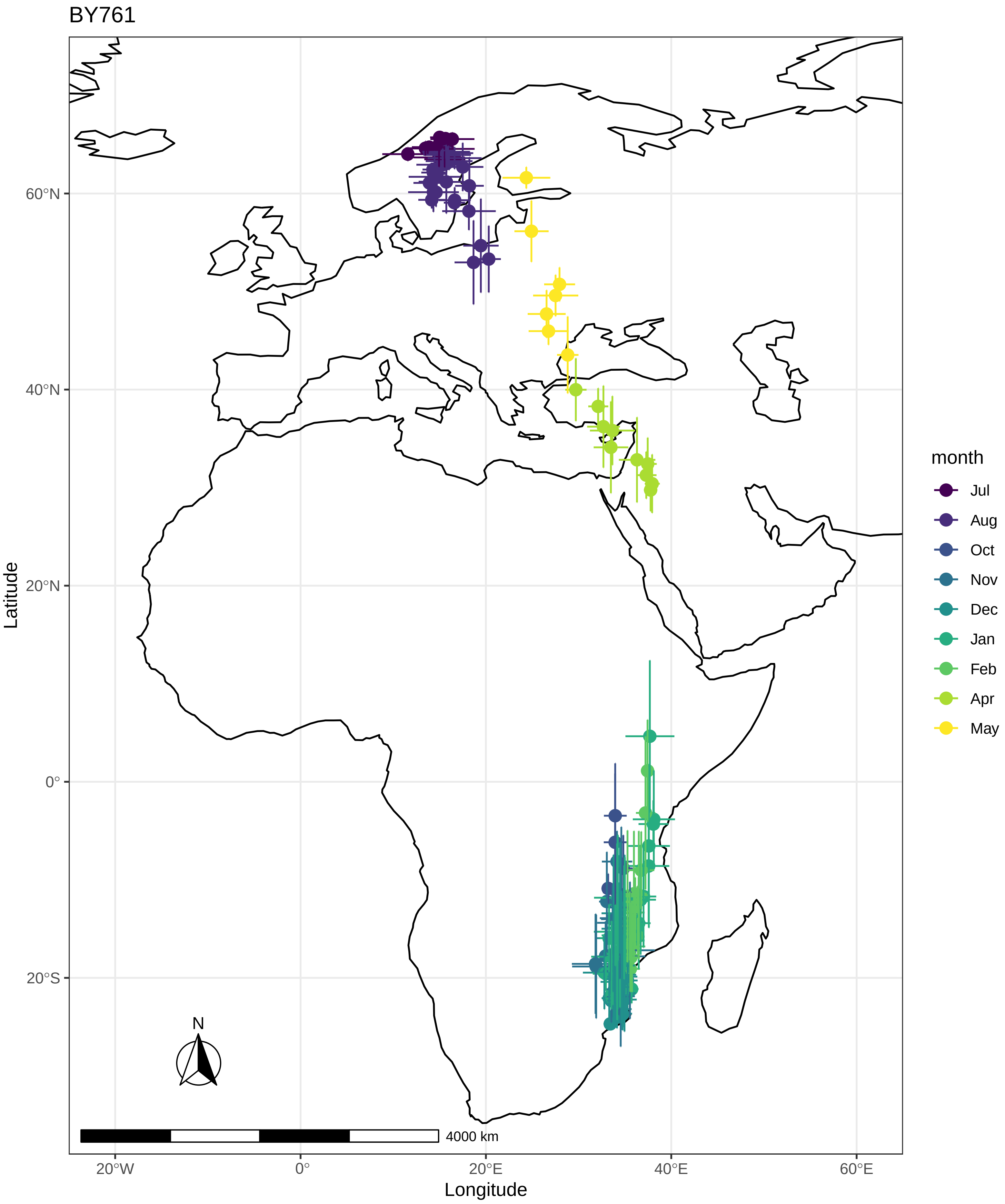
BY761
60°N
40°N
20°N
0°
20°S
Latitude
20°W
0°
20°E
40°E
60°E
Longitude
N
4000 km
month
Jul
Aug
Oct
Nov
Dec
Jan
Feb
Apr
May

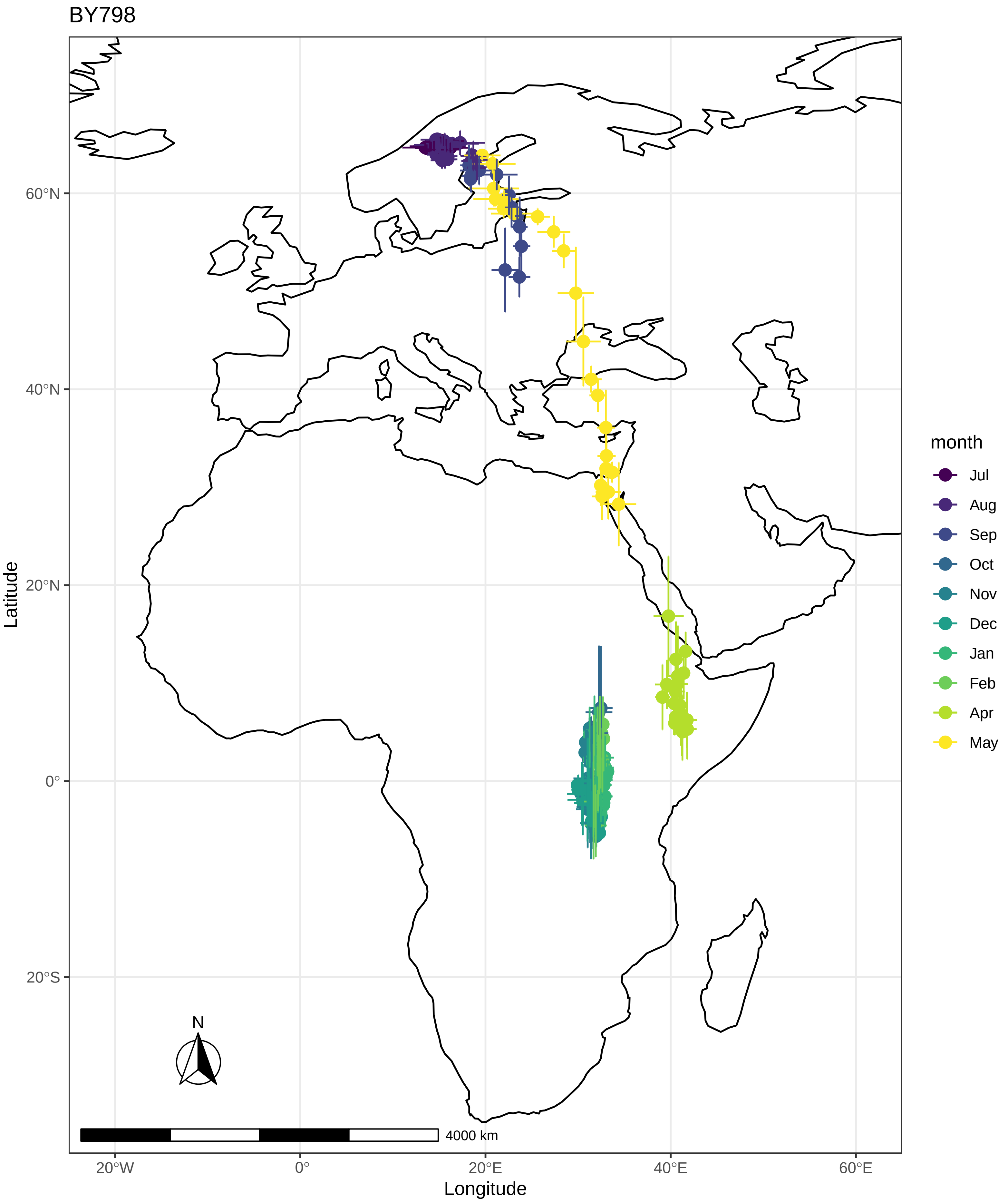
BY798
60°N
40°N
20°N
0°
20°S
Latitude
20°W
0°
20°E
40°E
60°E
Longitude
month
Jul
Aug
Sep
Oct
Nov
Dec
Jan
Feb
Apr
May
N
4000 km

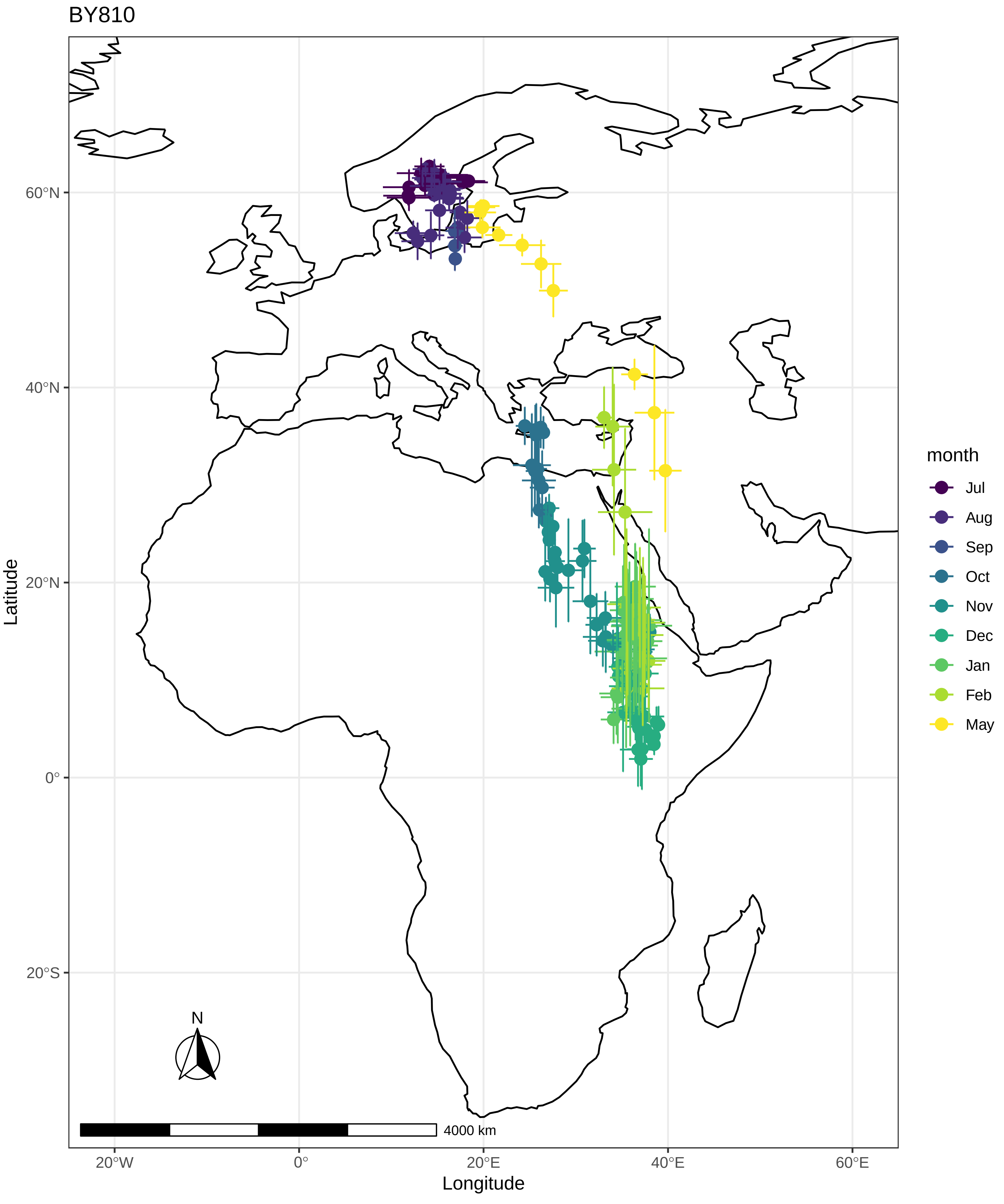
BY810
60°N
40°N
20°N
0°
20°S
Latitude
20°W
0°
20°E
40°E
60°E
Longitude
N
4000 km
month
Jul
Aug
Sep
Oct
Nov
Dec
Jan
Feb
May

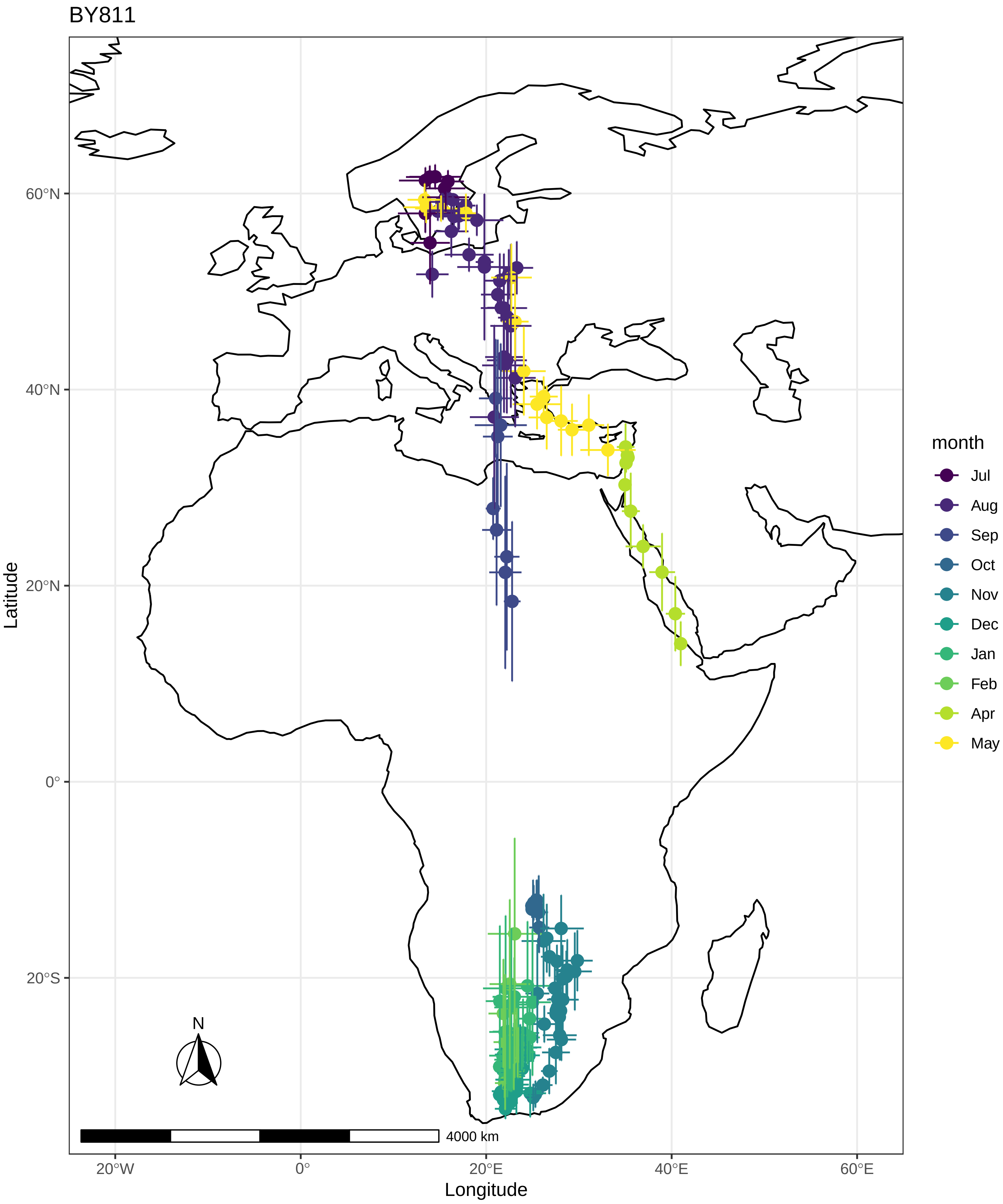
BY811
60°N
40°N
20°N
0°
20°S
Latitude
20°W
0°
20°E
40°E
60°E
Longitude
N
4000 km
month
Jul
Aug
Sep
Oct
Nov
Dec
Jan
Feb
Apr
May

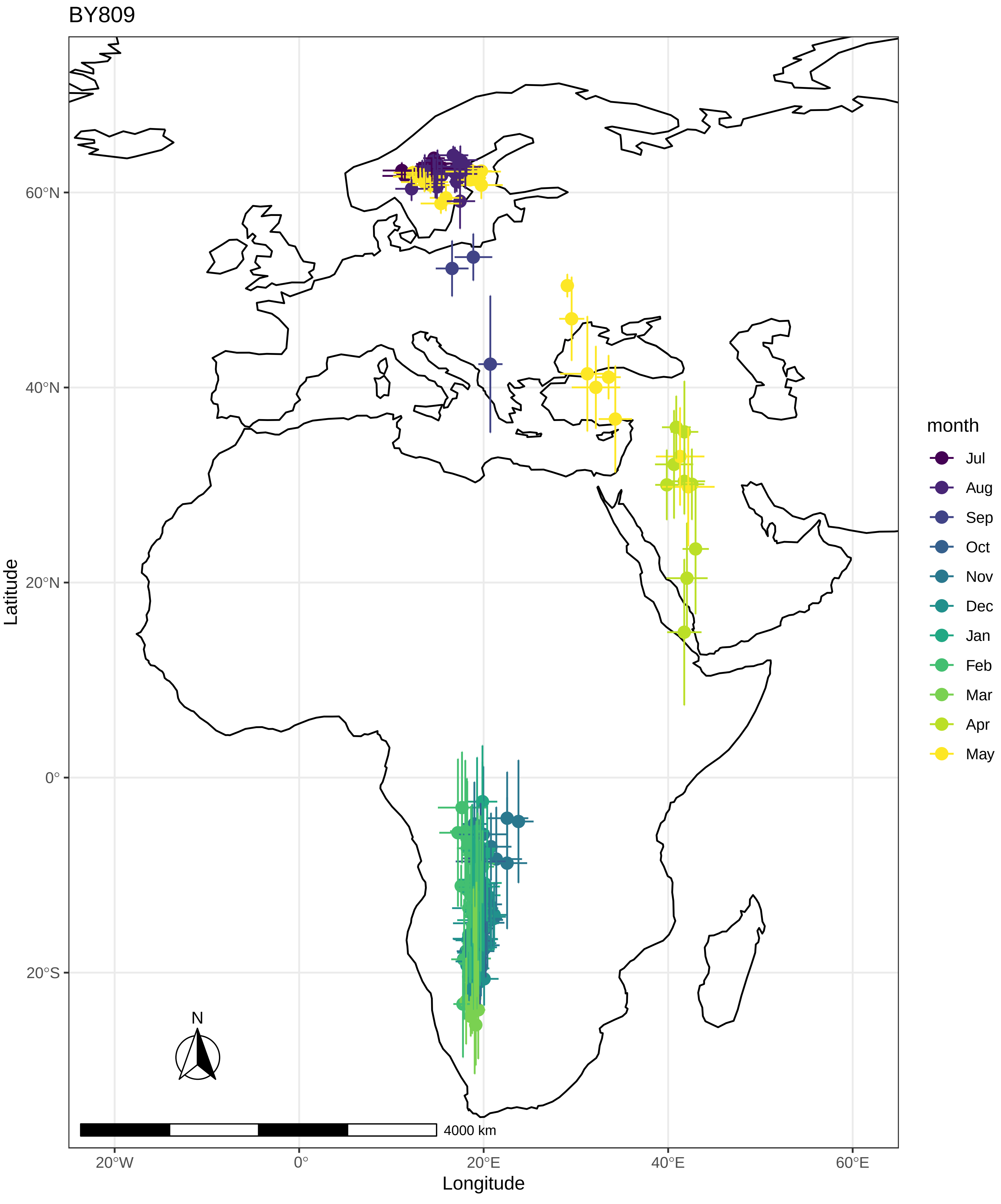

BY809
Latitude
60°N
40°N
20°N
0°
20°S
Longitude
20°W
0°
20°E
40°E
60°E
N
4000 km
month
Jul
Aug
Sep
Oct
Nov
Dec
Jan
Feb
Mar
Apr
May

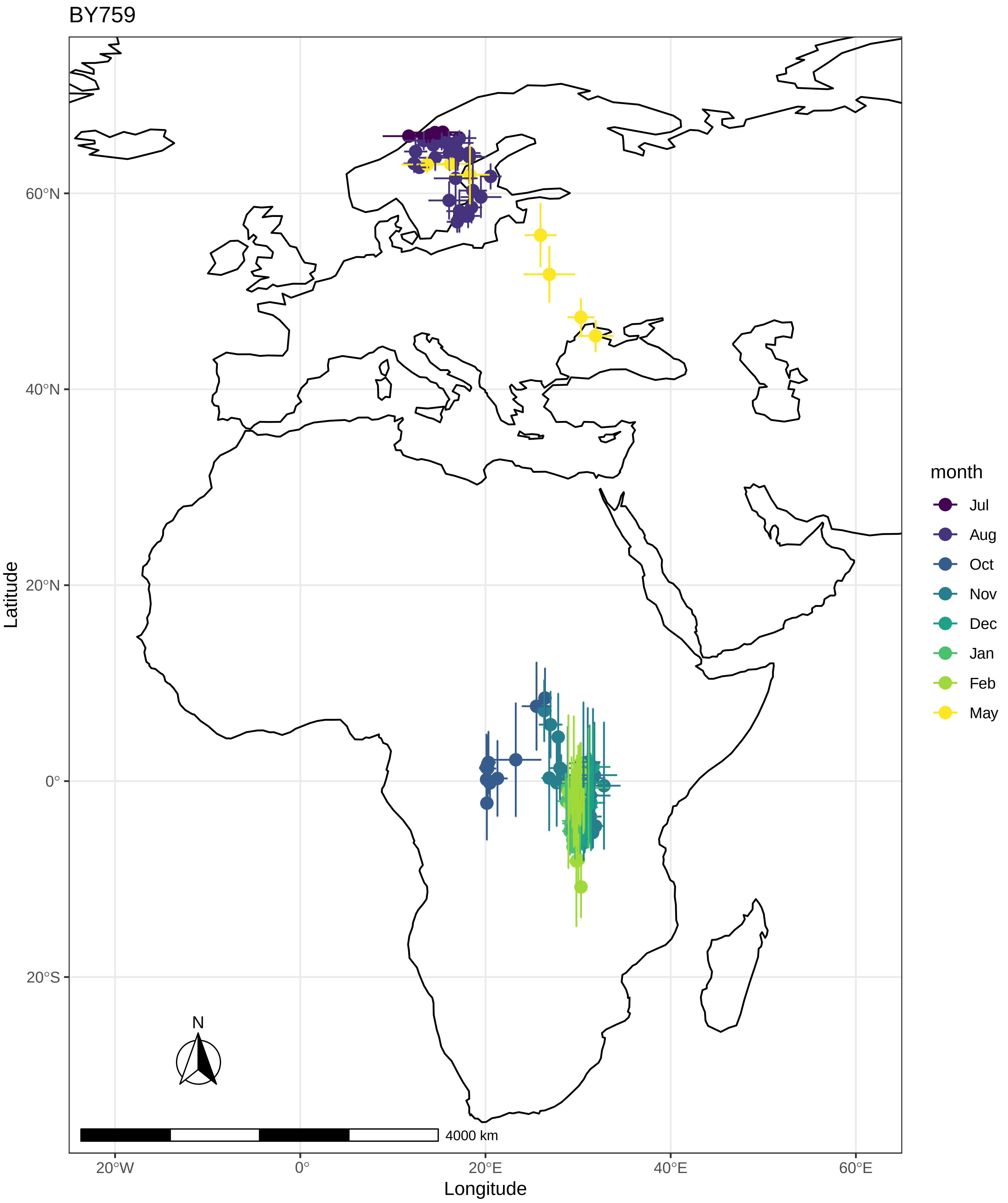
BY759
60°N
40°N
20°N
0°
20°S
Latitude
20°W
0°
20°E
40°E
60°E
Longitude
N
4000 km
month
Jul
Aug
Oct
Nov
Dec
Jan
Feb
May

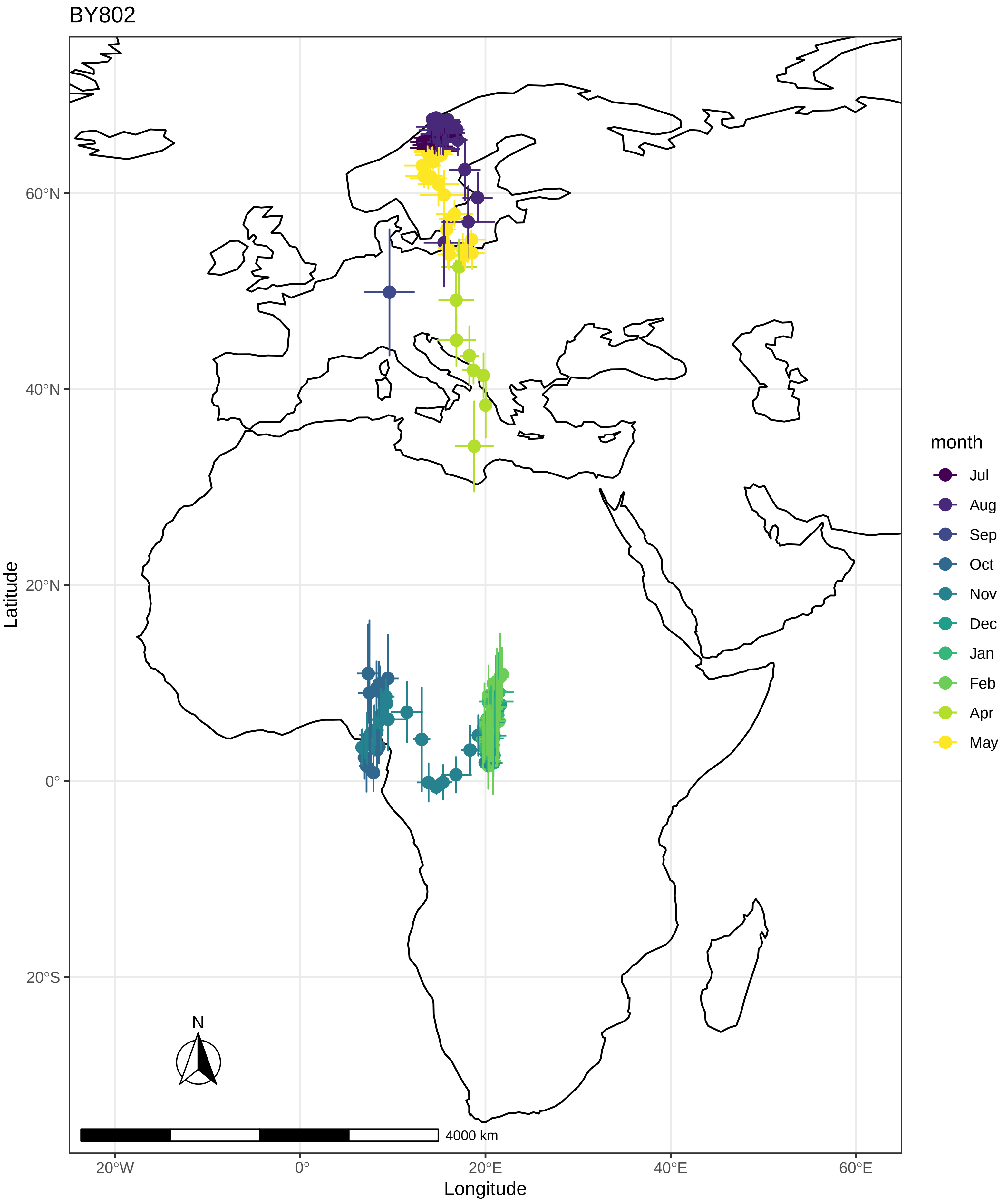
BY802
60°N
40°N
20°N
0°
20°S
Latitude
20°W
0°
20°E
40°E
60°E
Longitude
month
Jul
Aug
Sep
Oct
Nov
Dec
Jan
Feb
Apr
May
N
4000 km

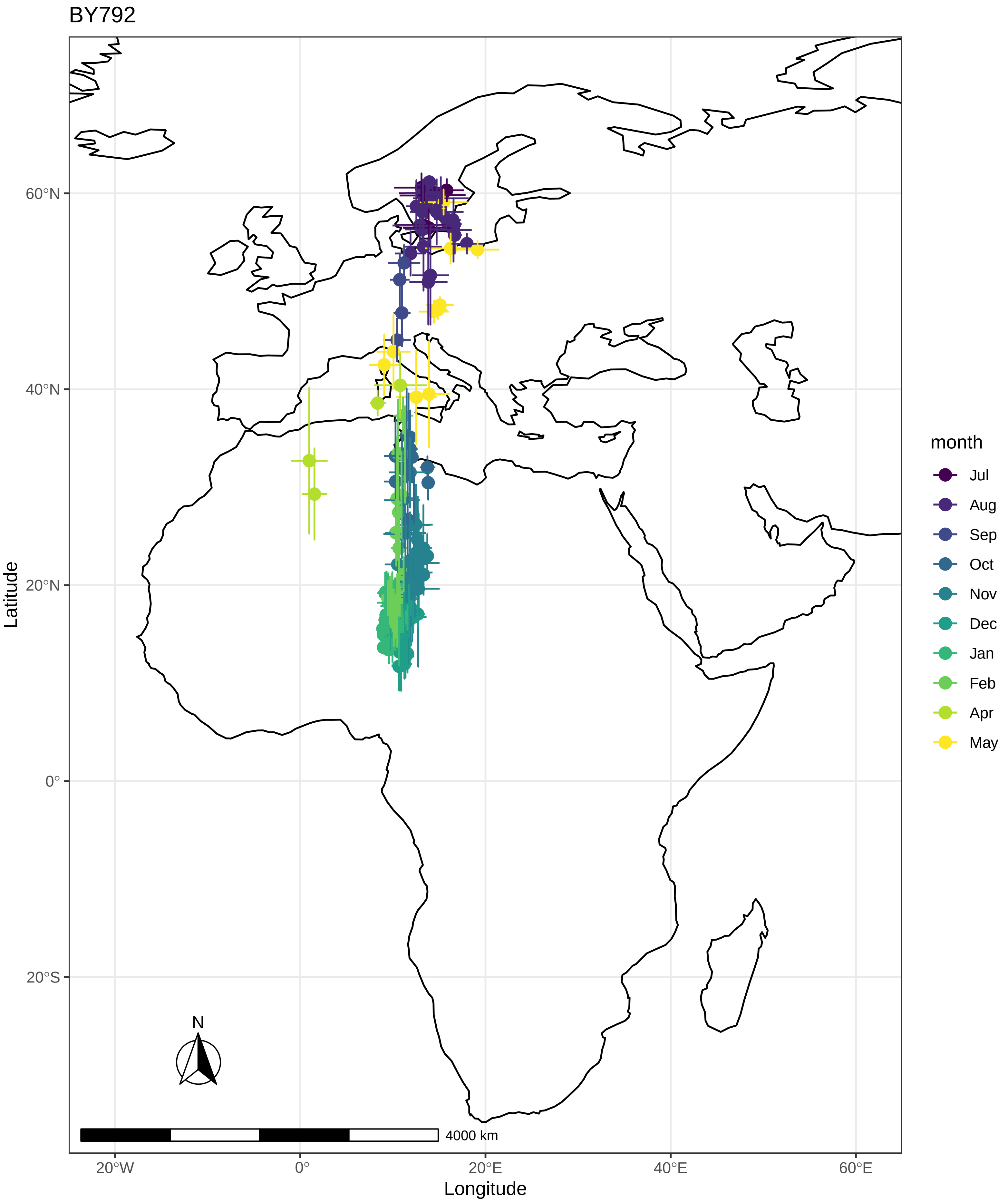
BY792
60°N
40°N
20°N
0°
20°S
Latitude
20°W
0°
20°E
40°E
60°E
Longitude
N
4000 km
month
Jul
Aug
Sep
Oct
Nov
Dec
Jan
Feb
Apr
May

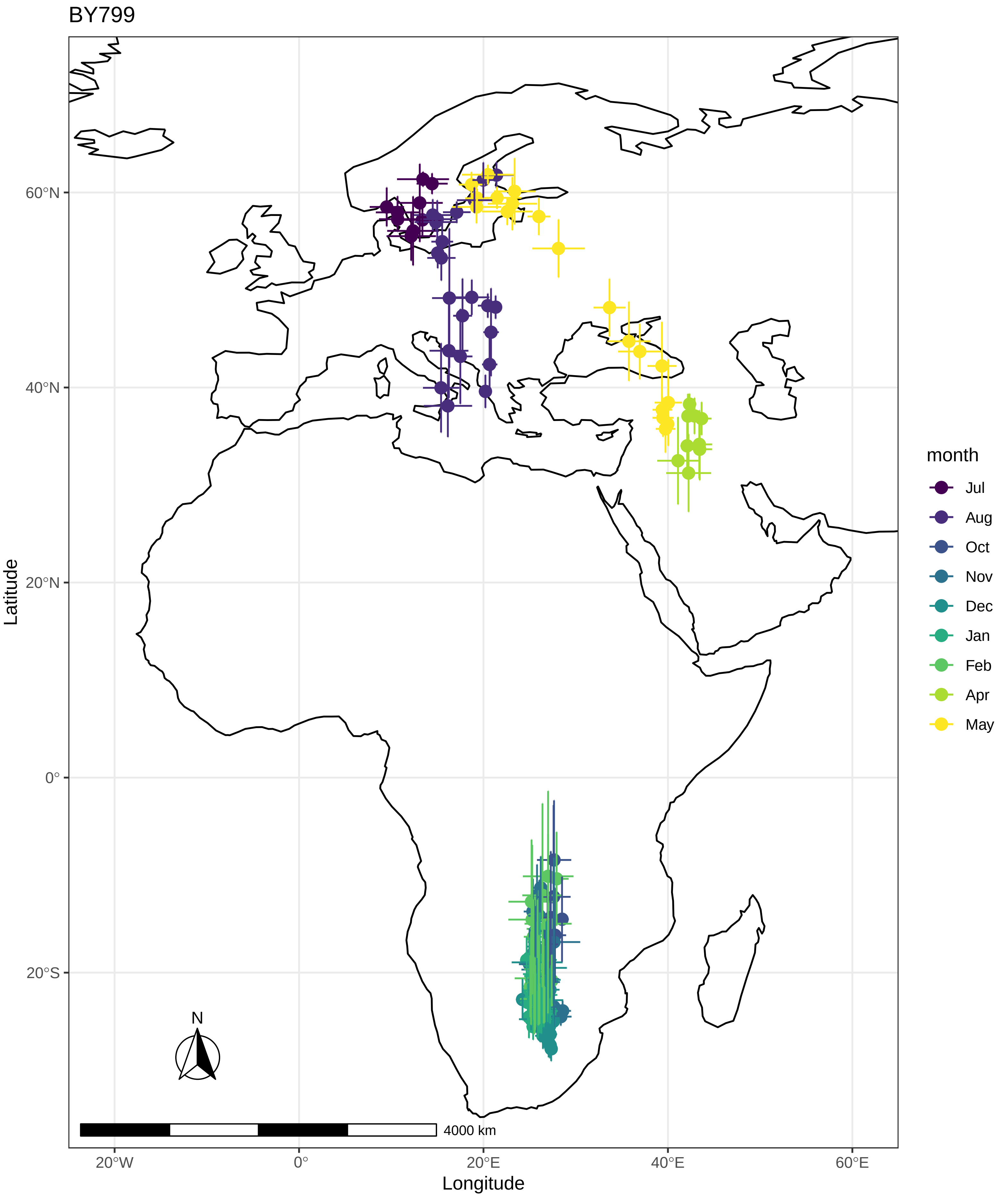

BY799
60°N
40°N
20°N
0°
20°S
20°W
0°
20°E
40°E
60°E
Latitude
Longitude
N
4000 km
month
Jul
Aug
Oct
Nov
Dec
Jan
Feb
Apr
May

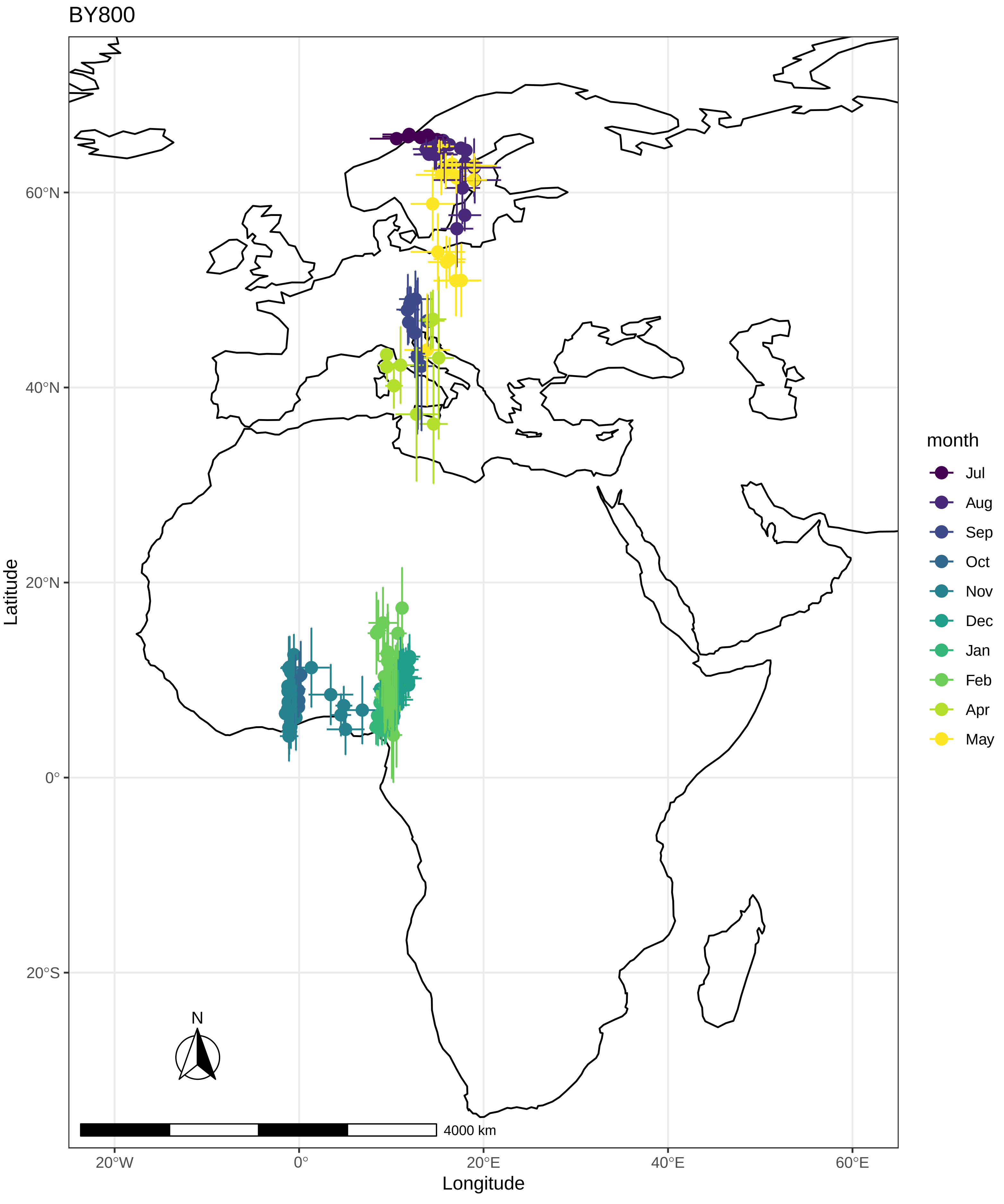
BY800
60°N
40°N
20°N
0°
20°S
Latitude
20°W
0°
20°E
40°E
60°E
Longitude
N
4000 km
month
Jul
Aug
Sep
Oct
Nov
Dec
Jan
Feb
Apr
May

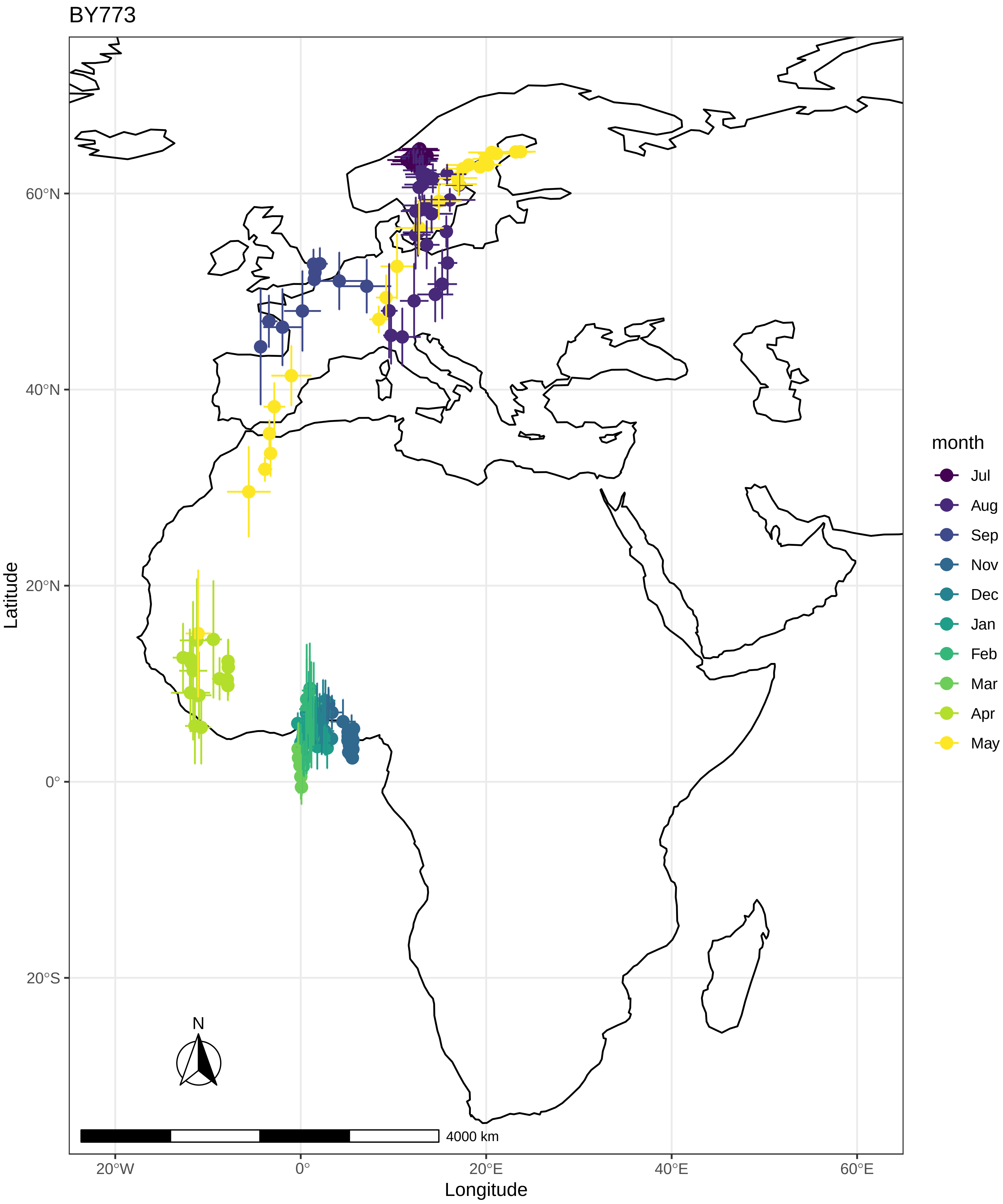

BY773
60°N
40°N
20°N
0°
20°S
Latitude
20°W
0°
20°E
40°E
60°E
Longitude
N
4000 km
month
Jul
Aug
Sep
Nov
Dec
Jan
Feb
Mar
Apr
May

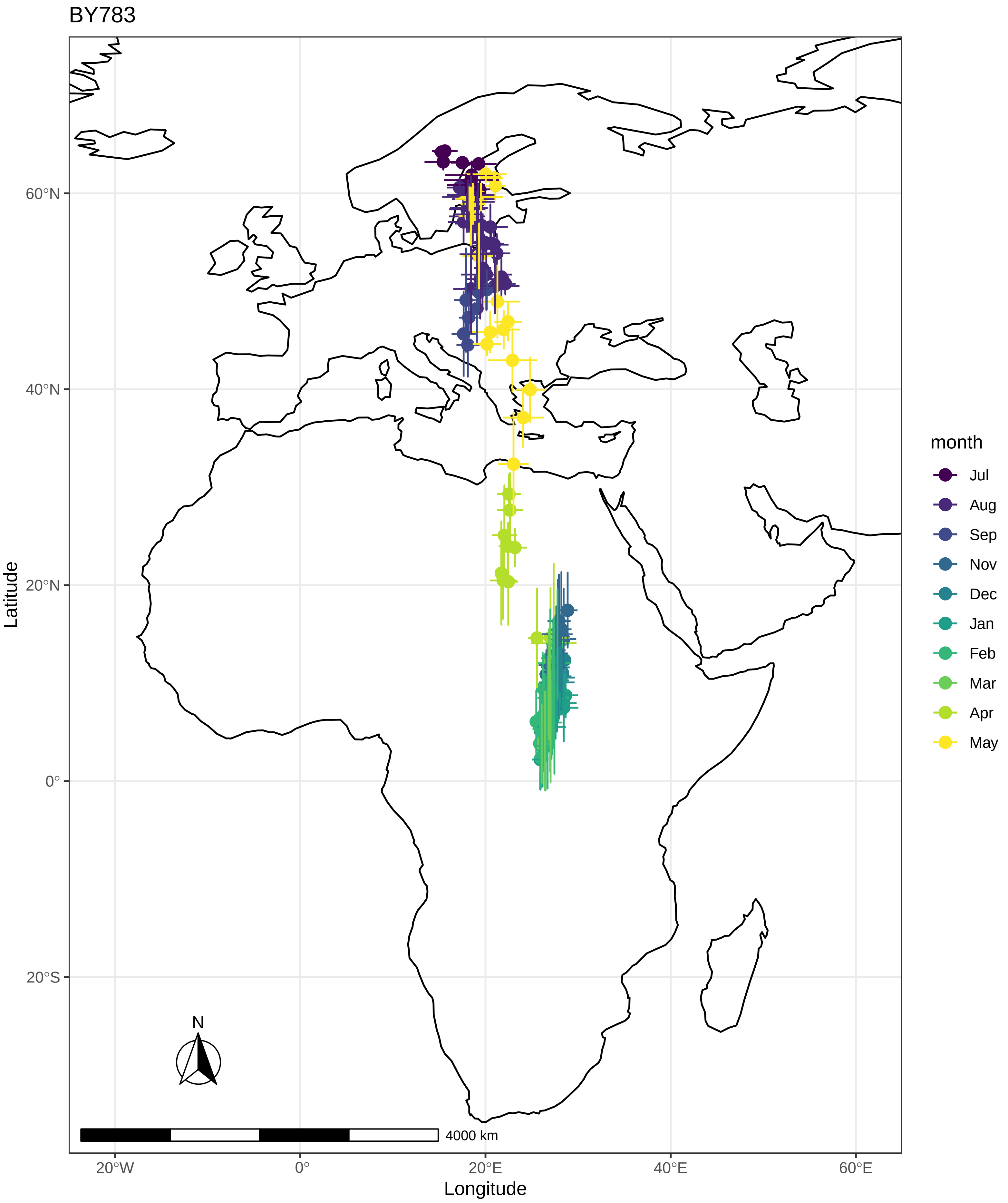
BY783
60°N
40°N
20°N
0°
20°S
Latitude
20°W
0°
20°E
40°E
60°E
Longitude
N
4000 km
month
Jul
Aug
Sep
Nov
Dec
Jan
Feb
Mar
Apr
May

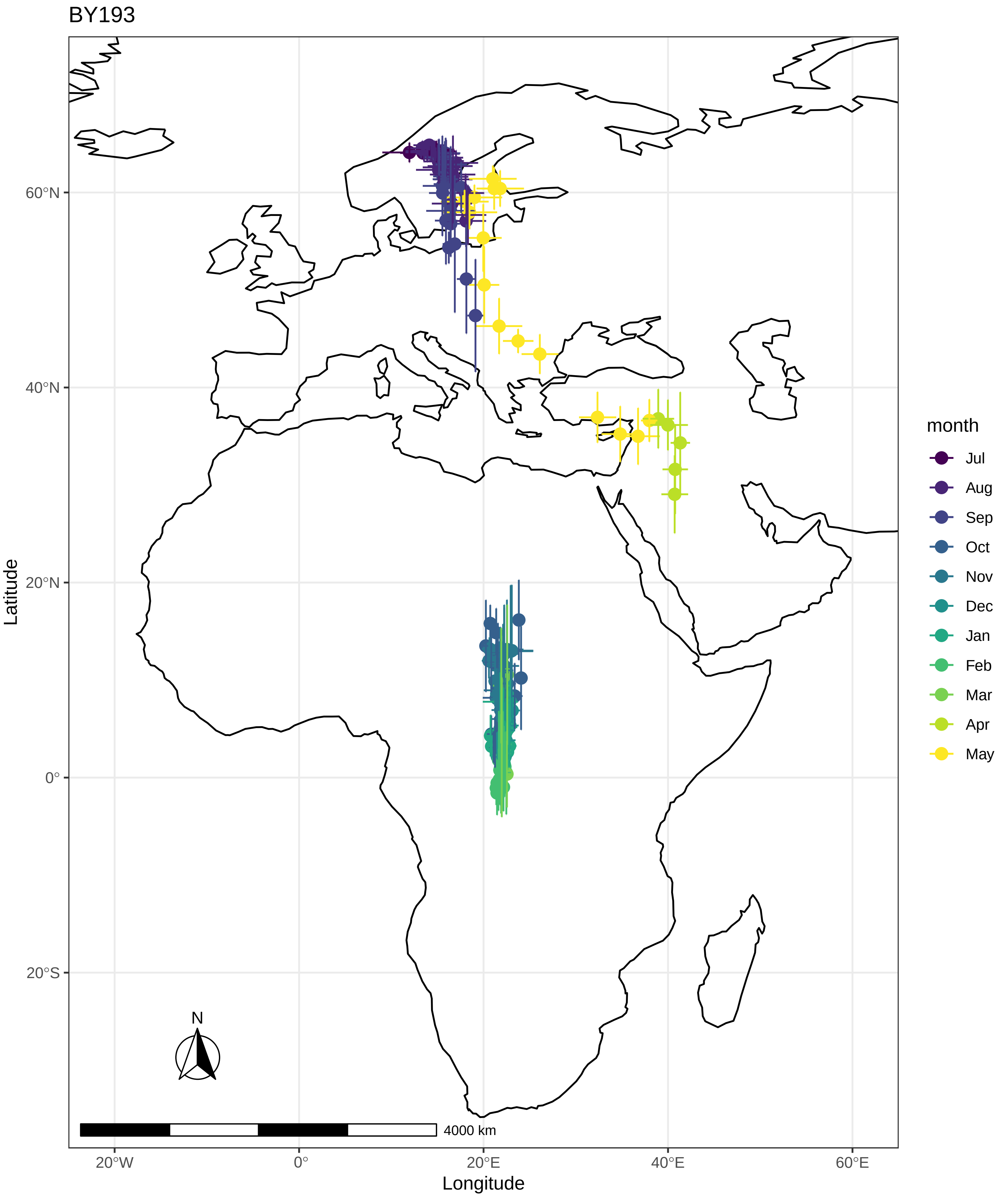

BY193
60°N
40°N
20°N
0°
20°S
Latitude
20°W
0°
20°E
40°E
60°E
Longitude
N
4000 km
month
Jul
Aug
Sep
Oct
Nov
Dec
Jan
Feb
Mar
Apr
May

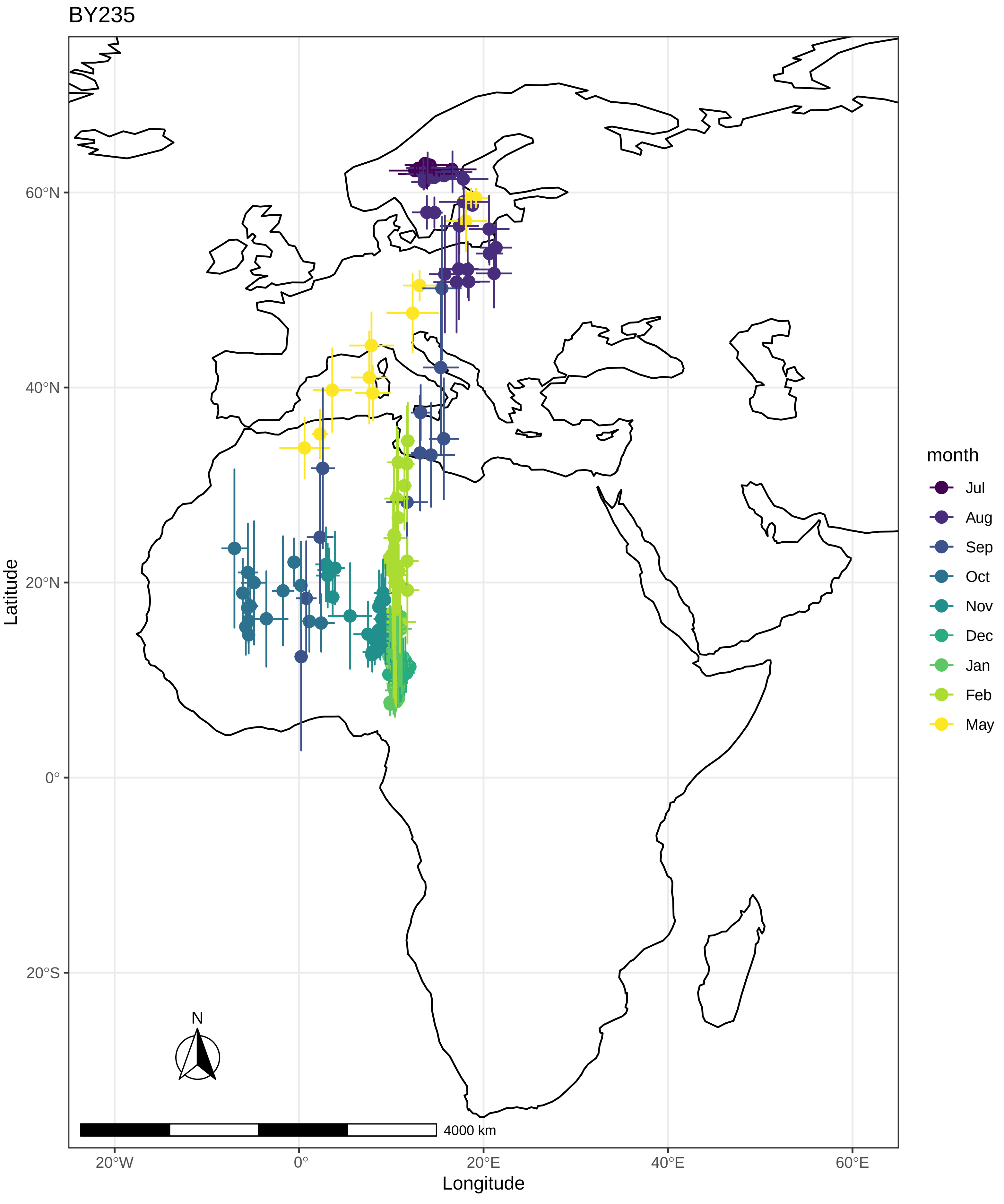

BY235
60°N
40°N
20°N
0°
20°S
20°W
0°
20°E
40°E
60°E
Latitude
Longitude
N
4000 km
month
Jul
Aug
Sep
Oct
Nov
Dec
Jan
Feb
May

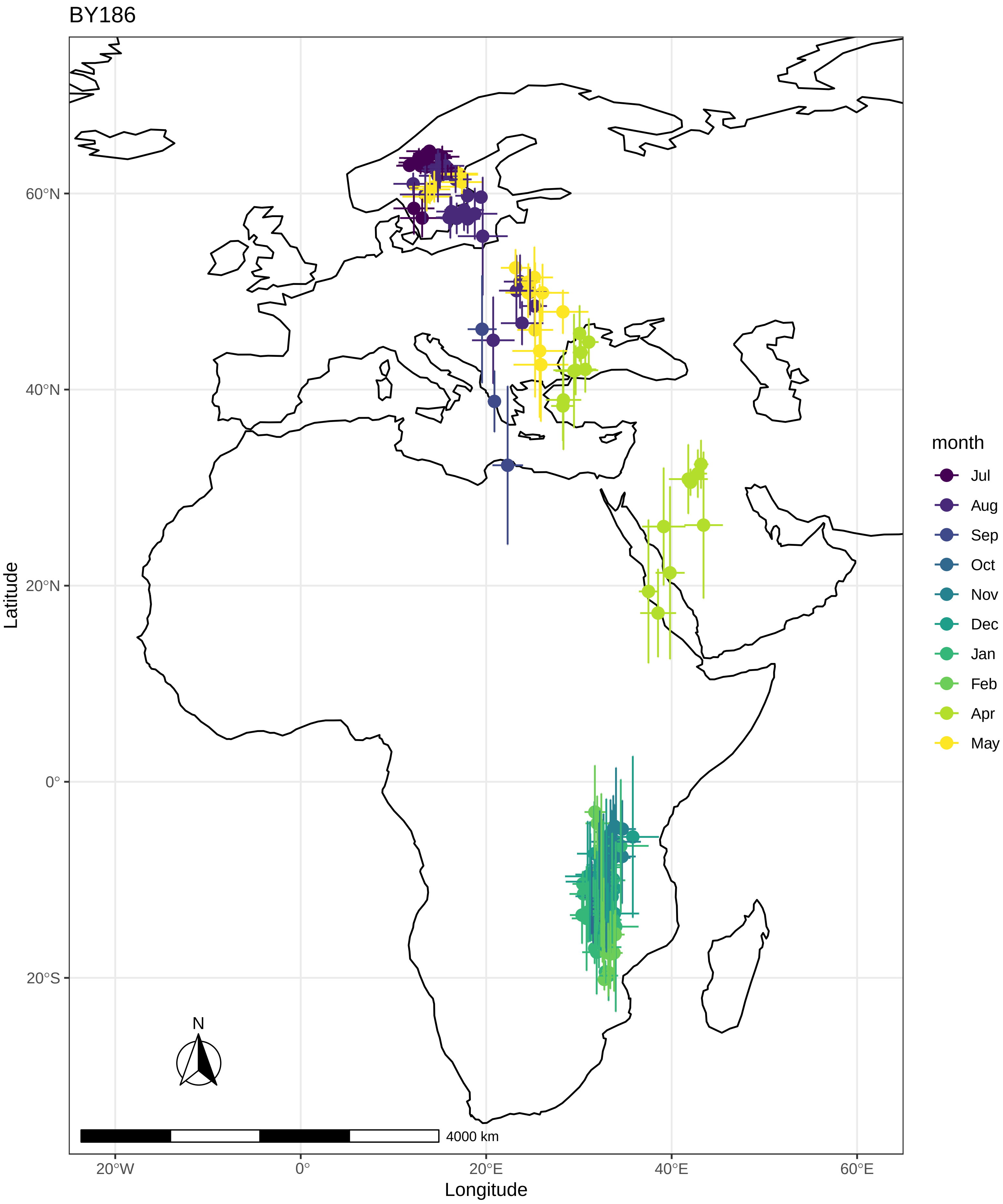
BY186
60°N
40°N
20°N
0°
20°S
N
4000 km
20°W
0°
20°E
40°E
60°E
Longitude
Latitude
month
Jul
Aug
Sep
Oct
Nov
Dec
Jan
Feb
Apr
May

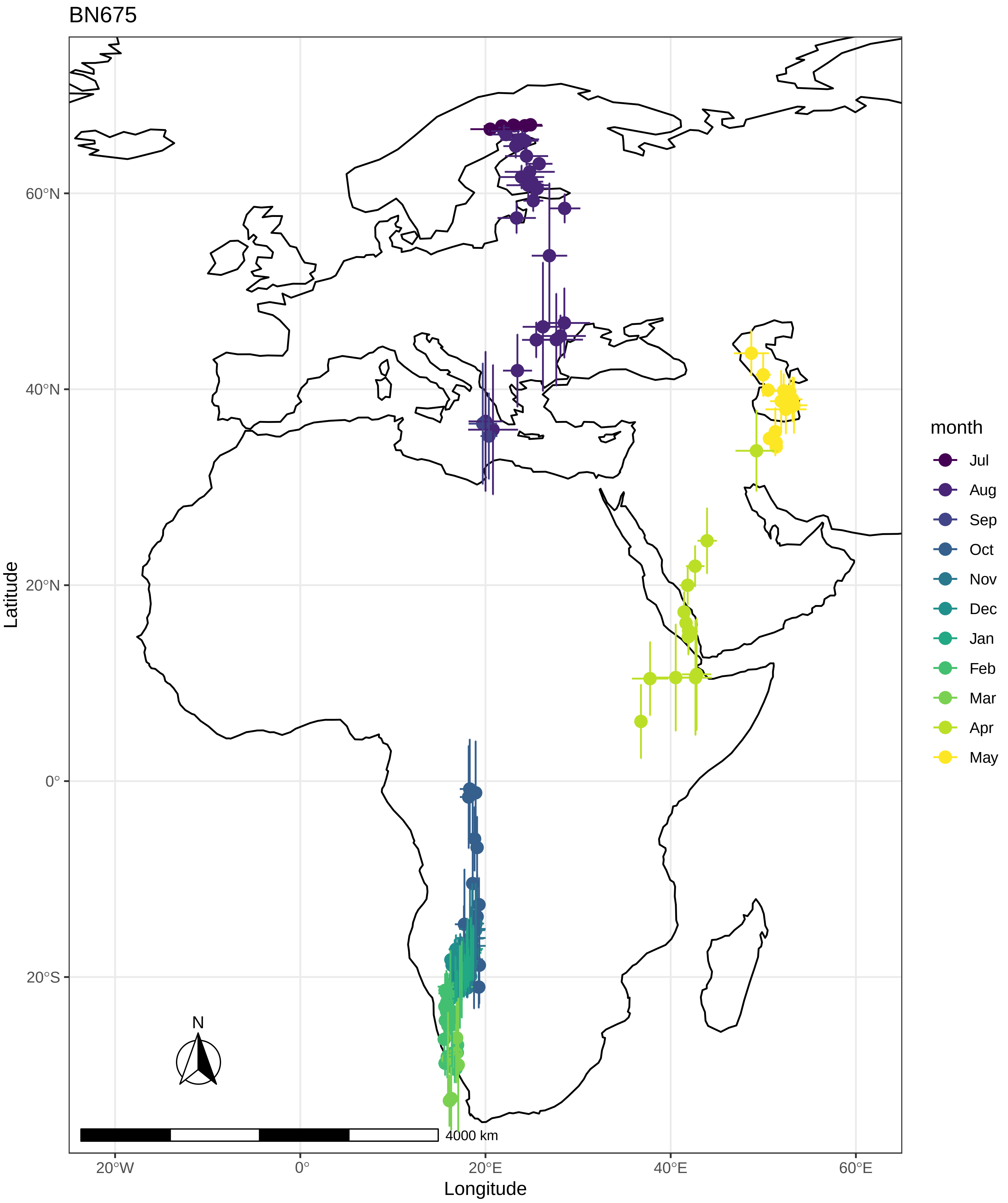
BN675
60°N
40°N
20°N
0°
20°S
Latitude
20°W
0°
20°E
40°E
60°E
Longitude
N
4000 km
month
Jul
Aug
Sep
Oct
Nov
Dec
Jan
Feb
Mar
Apr
May

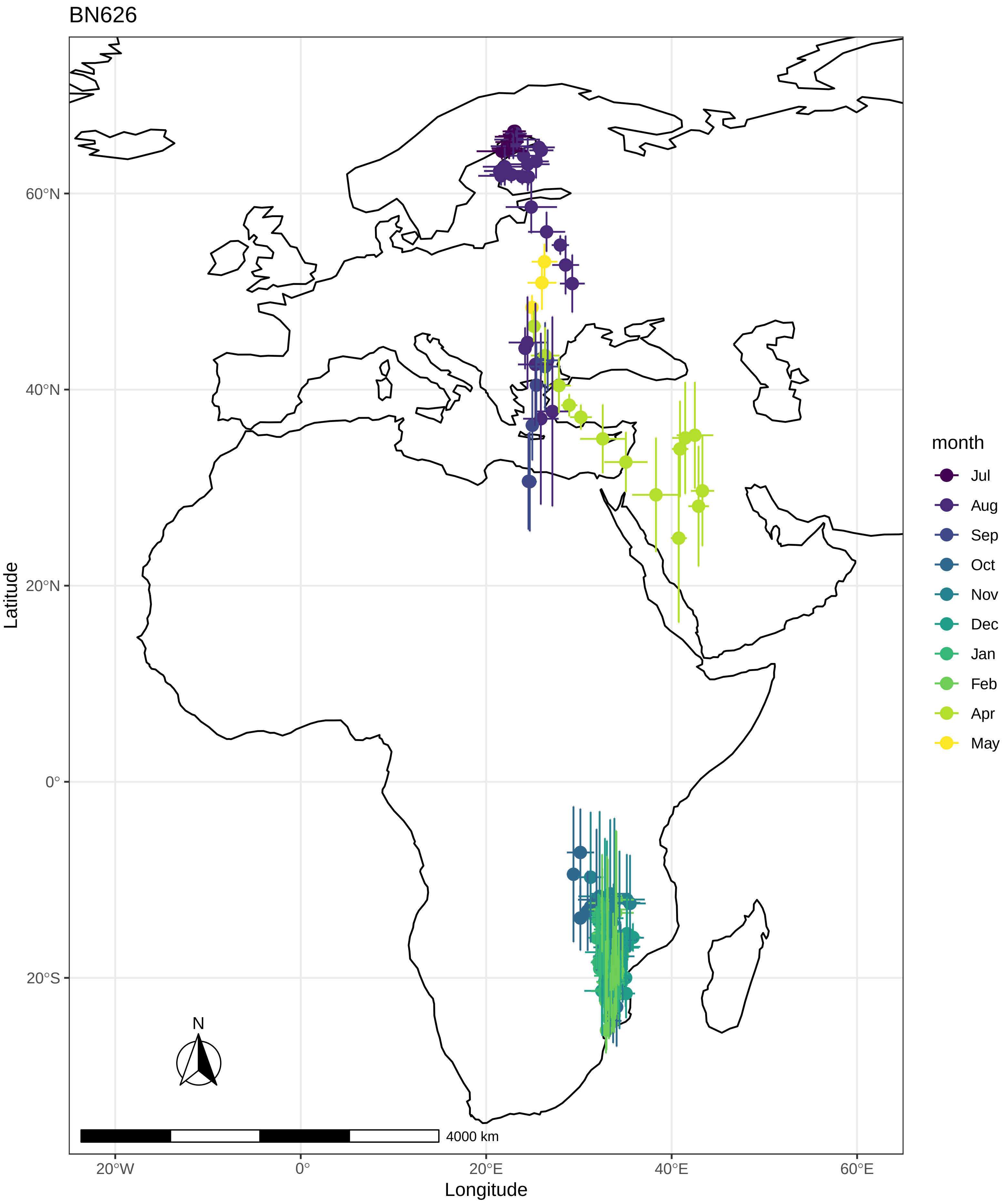
BN626
60°N
40°N
20°N
0°
20°S
Latitude
20°W
0°
20°E
40°E
60°E
Longitude
month
Jul
Aug
Sep
Oct
Nov
Dec
Jan
Feb
Apr
May
N
4000 km

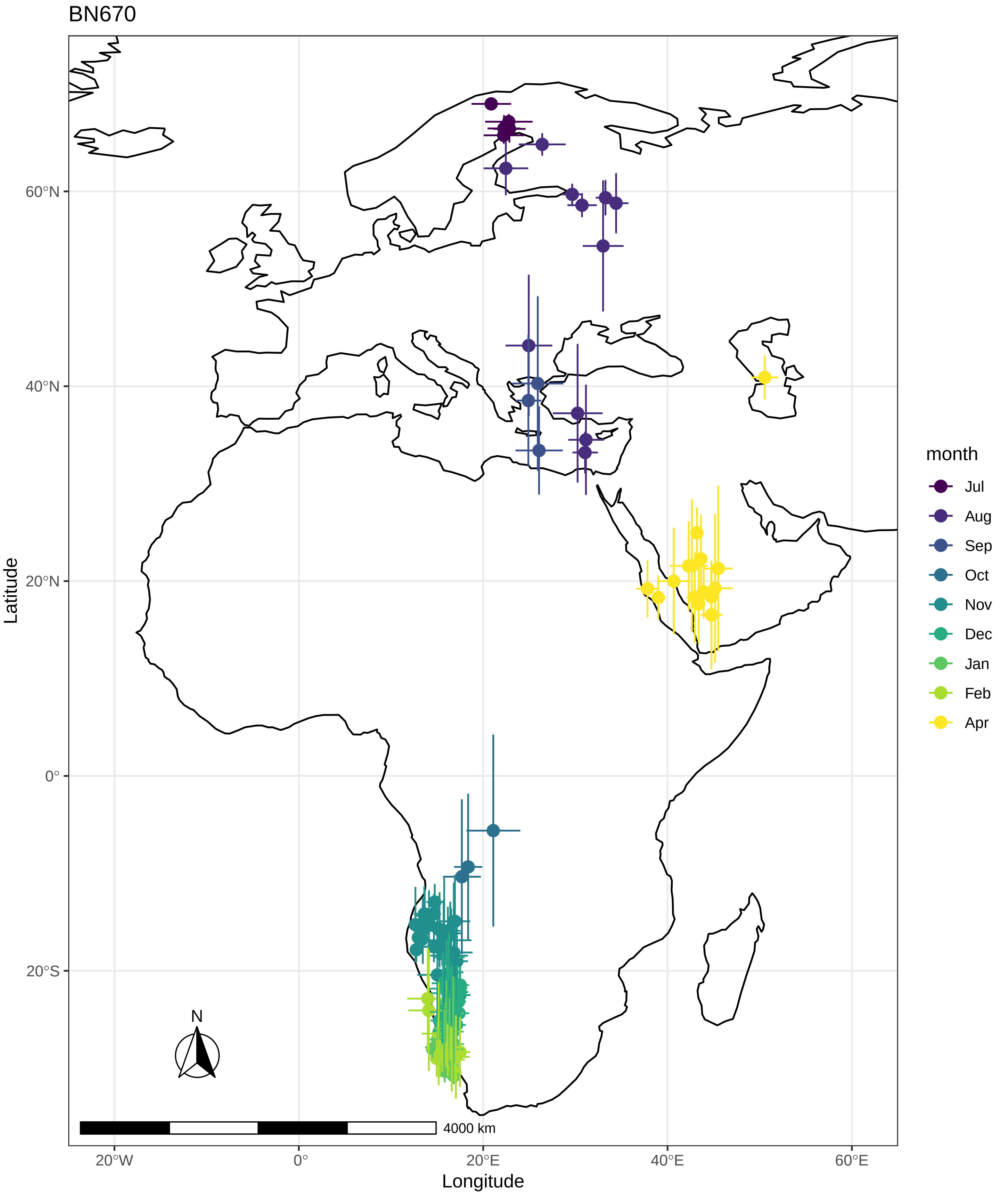

BN670
60°N
40°N
20°N
0°
20°S
20°W
0°
20°E
40°E
60°E
Latitude
Longitude
month
Jul
Aug
Sep
Oct
Nov
Dec
Jan
Feb
Apr
N
4000 km

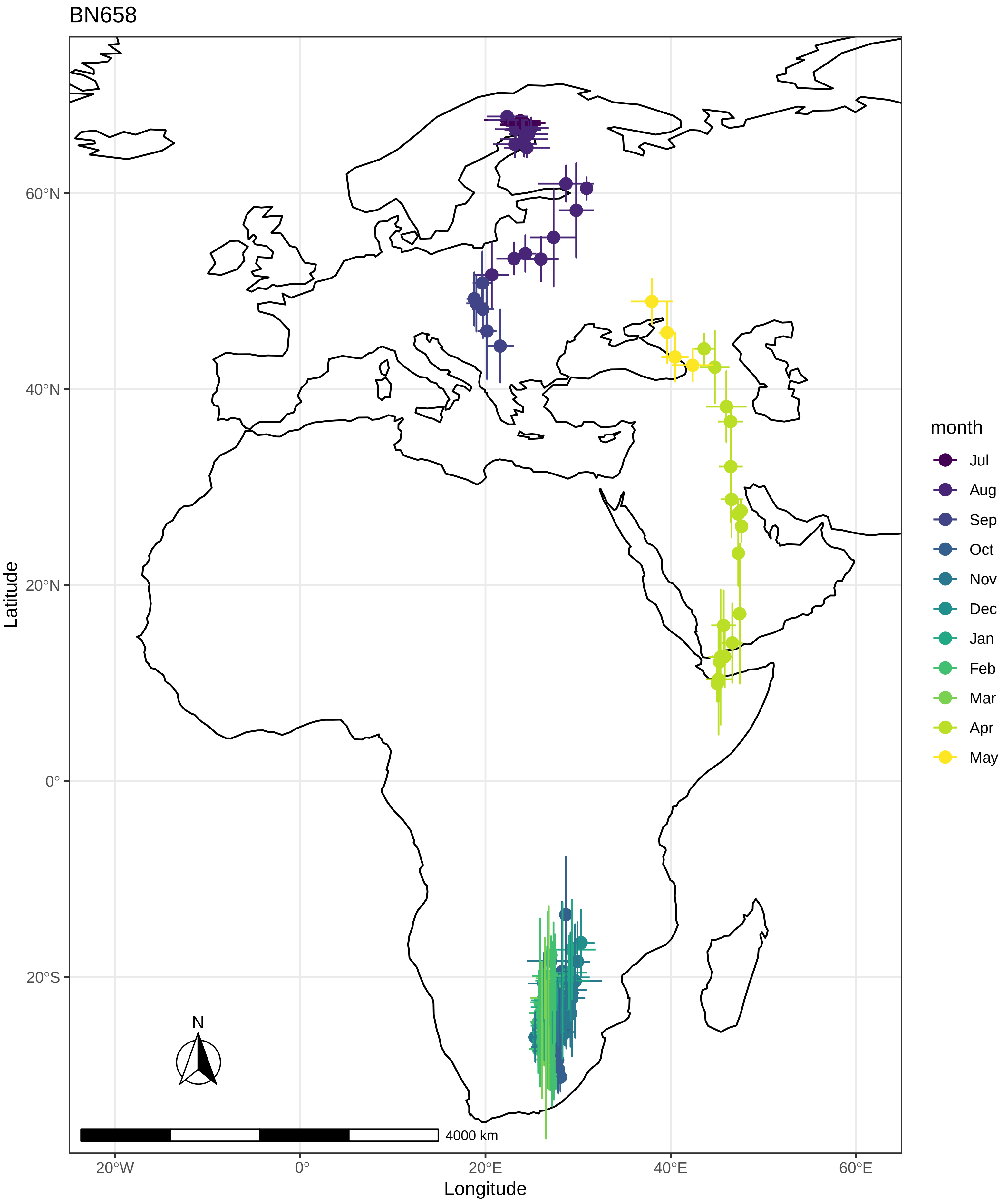

BN658
60°N
40°N
20°N
0°
20°S
20°W
0°
20°E
40°E
60°E
Latitude
Longitude
N
4000 km
month
Jul
Aug
Sep
Oct
Nov
Dec
Jan
Feb
Mar
Apr
May

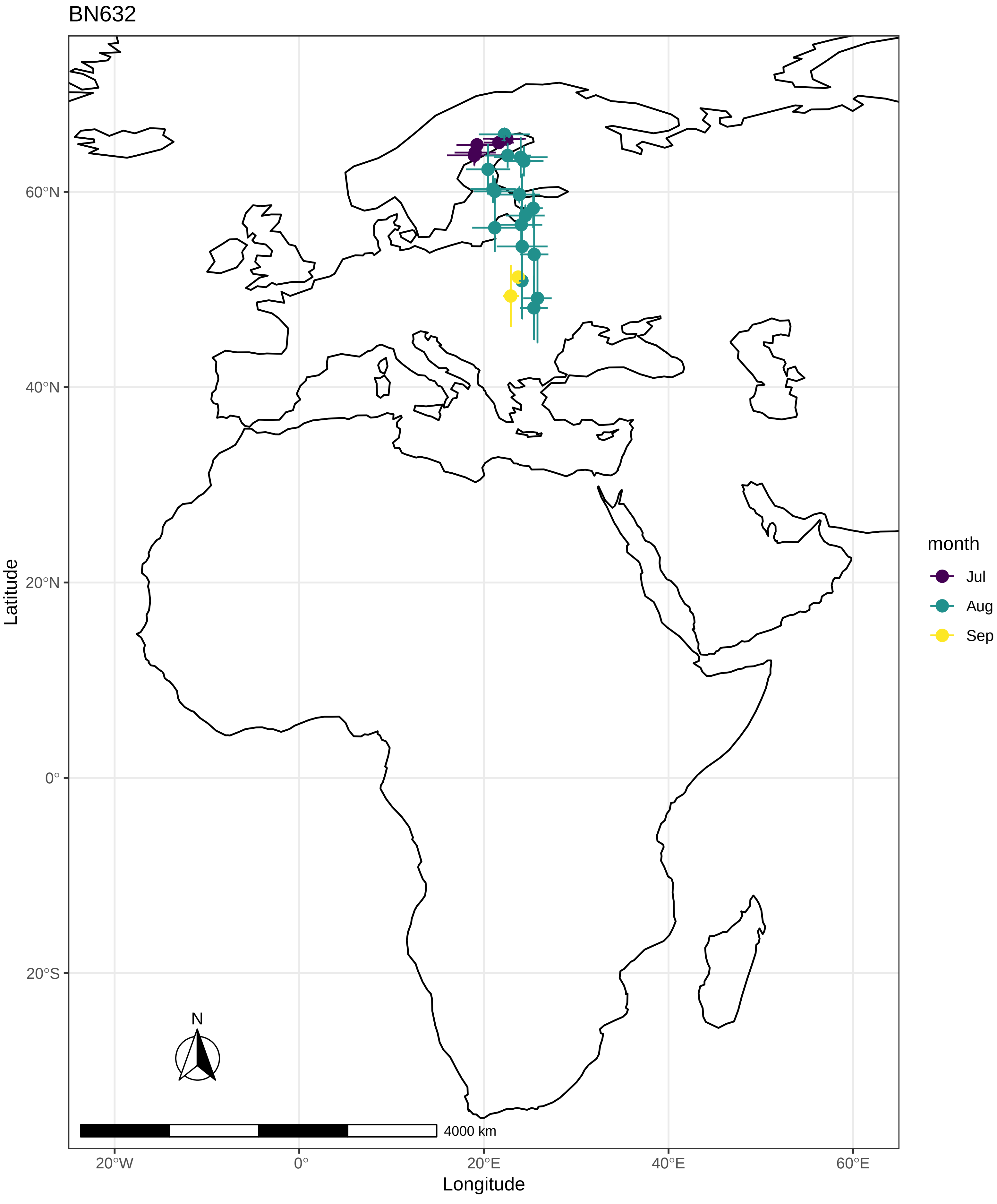

BN632
60°N
40°N
20°N
0°
20°S
Latitude
20°W
0°
20°E
40°E
60°E
Longitude
month
Jul
Aug
Sep
N
4000 km

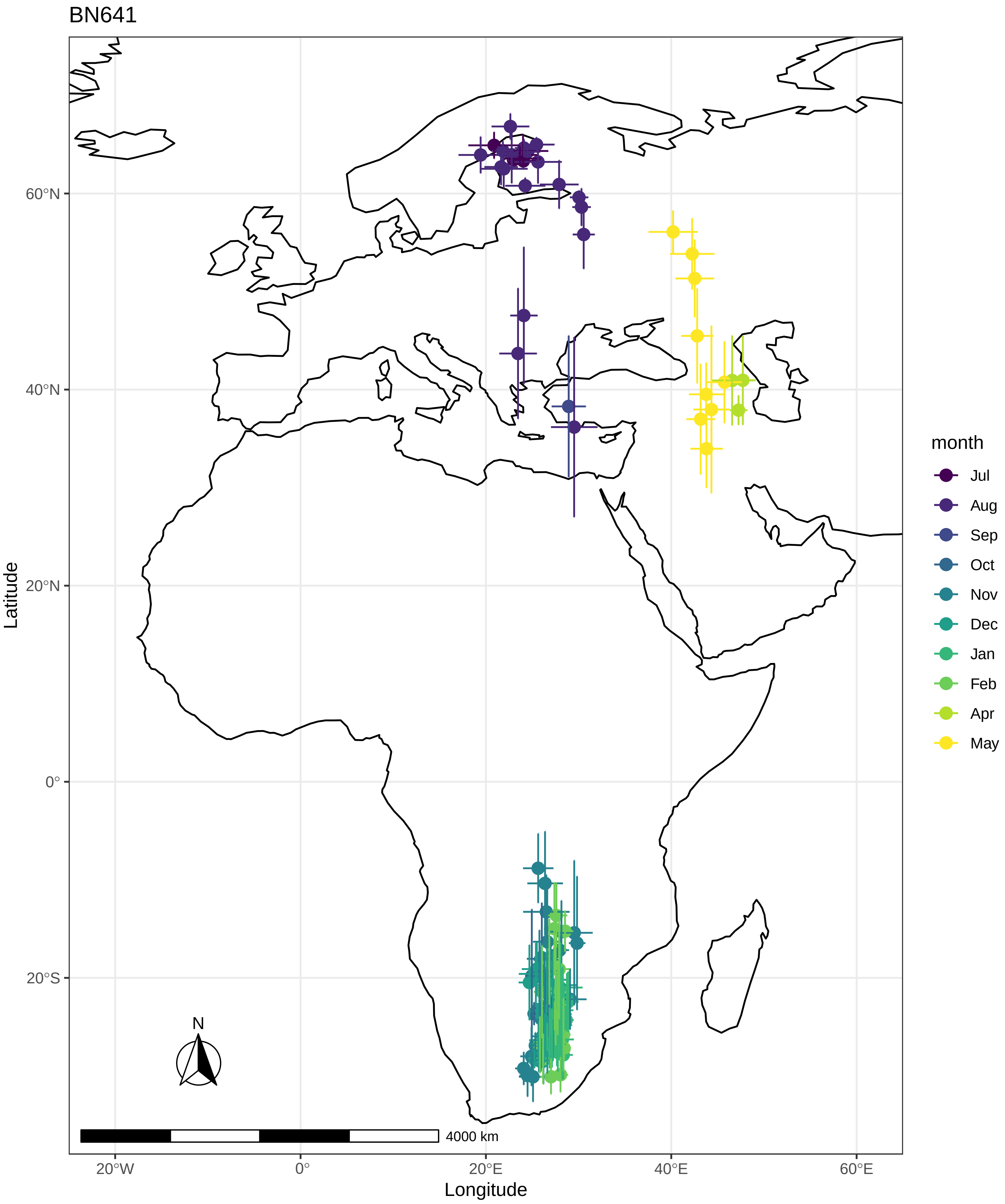
BN641
60°N
40°N
20°N
0°
20°S
Latitude
20°W
0°
20°E
40°E
60°E
Longitude
month
Jul
Aug
Sep
Oct
Nov
Dec
Jan
Feb
Apr
May
N
4000 km

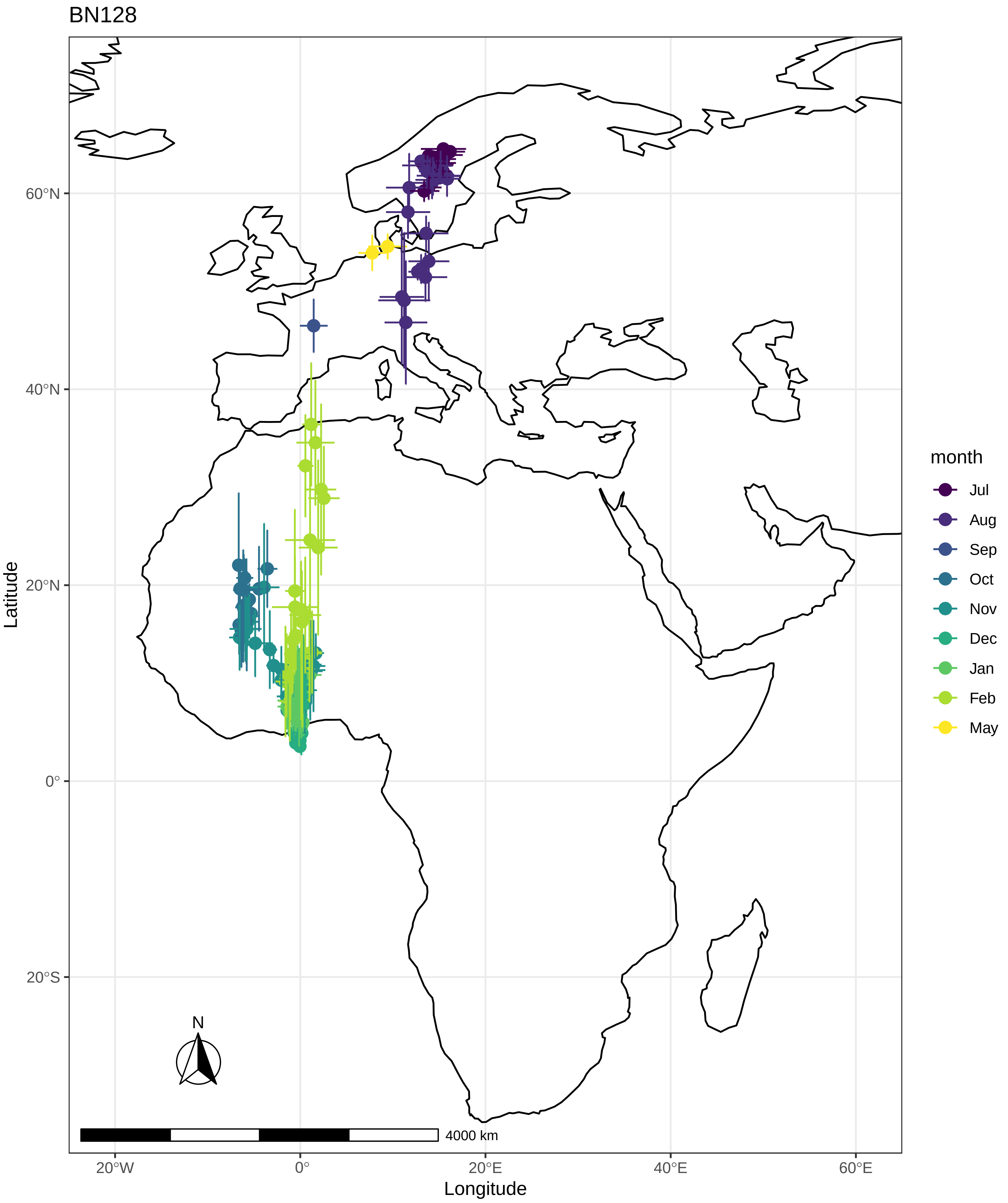

BN128
60°N
40°N
20°N
0°
20°S
Latitude
20°W
0°
20°E
40°E
60°E
Longitude
N
4000 km
month
Jul
Aug
Sep
Oct
Nov
Dec
Jan
Feb
May

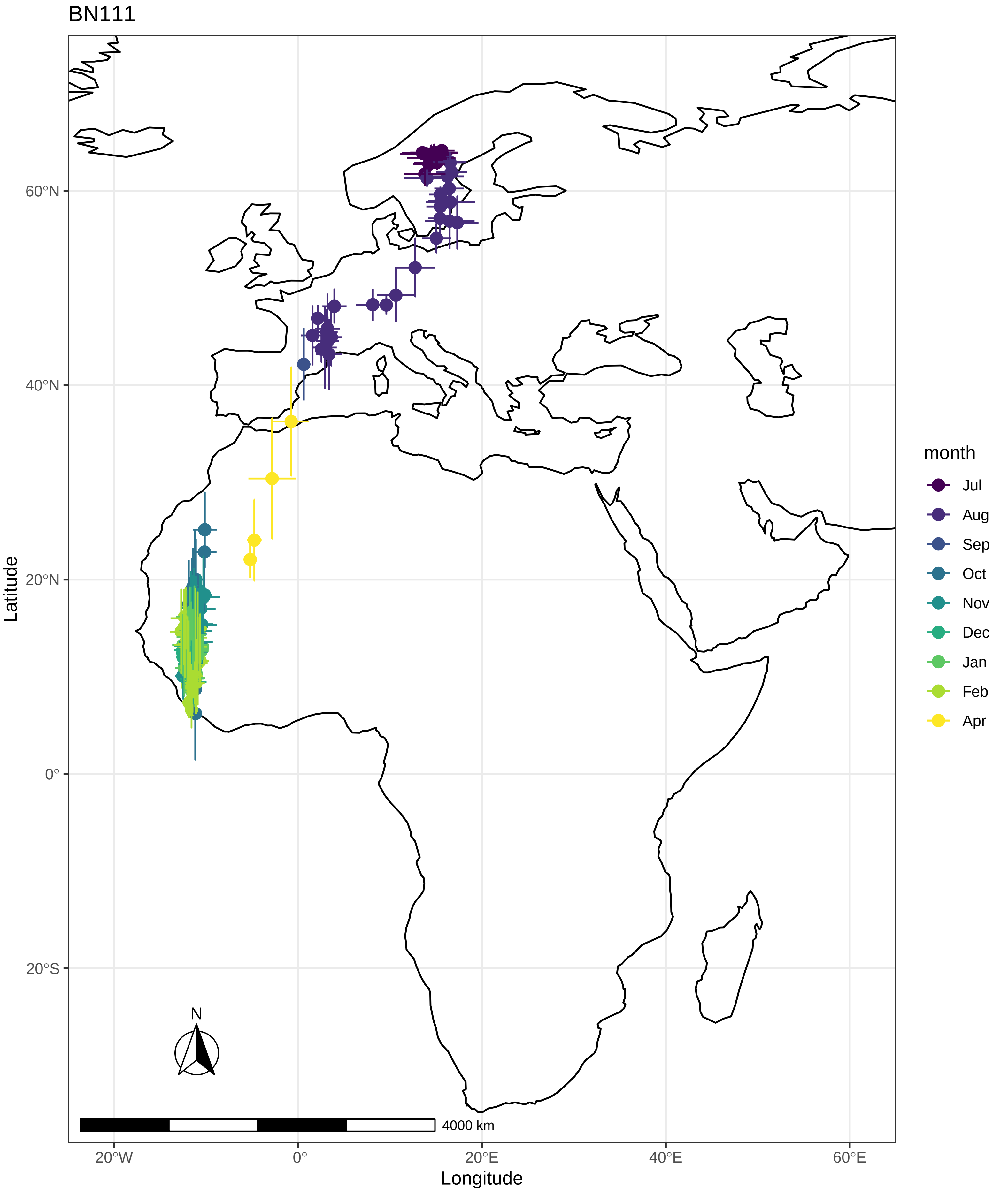

BN111
60°N
40°N
20°N
0°
20°S
Latitude
20°W
0°
20°E
40°E
60°E
Longitude
N
4000 km
month
Jul
Aug
Sep
Oct
Nov
Dec
Jan
Feb
Apr

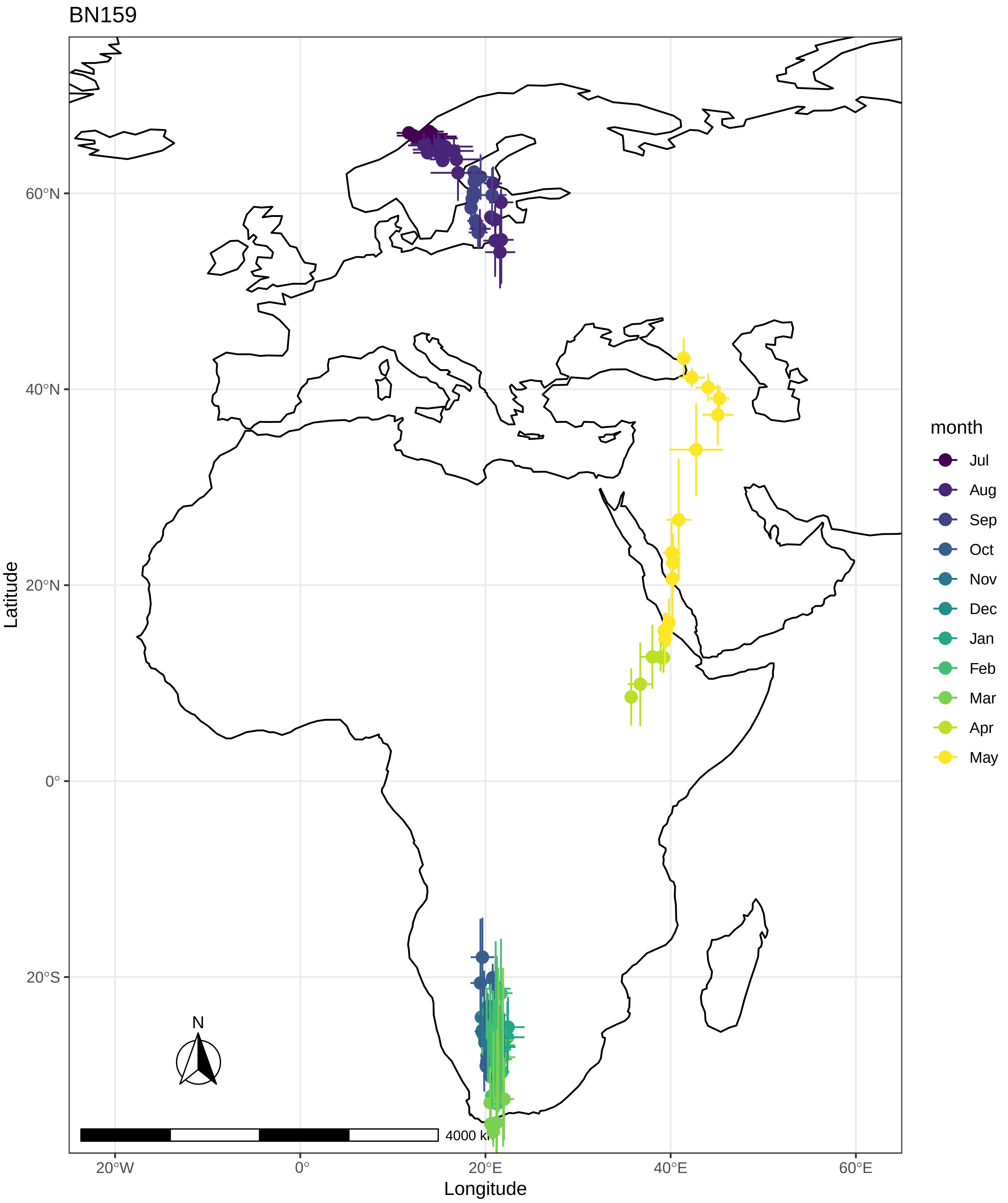

BN159
60°N
40°N
20°N
0°
20°S
20°W
0°
20°E
40°E
60°E
Longitude
Latitude
N
4000 km
month
Jul
Aug
Sep
Oct
Nov
Dec
Jan
Feb
Mar
Apr
May

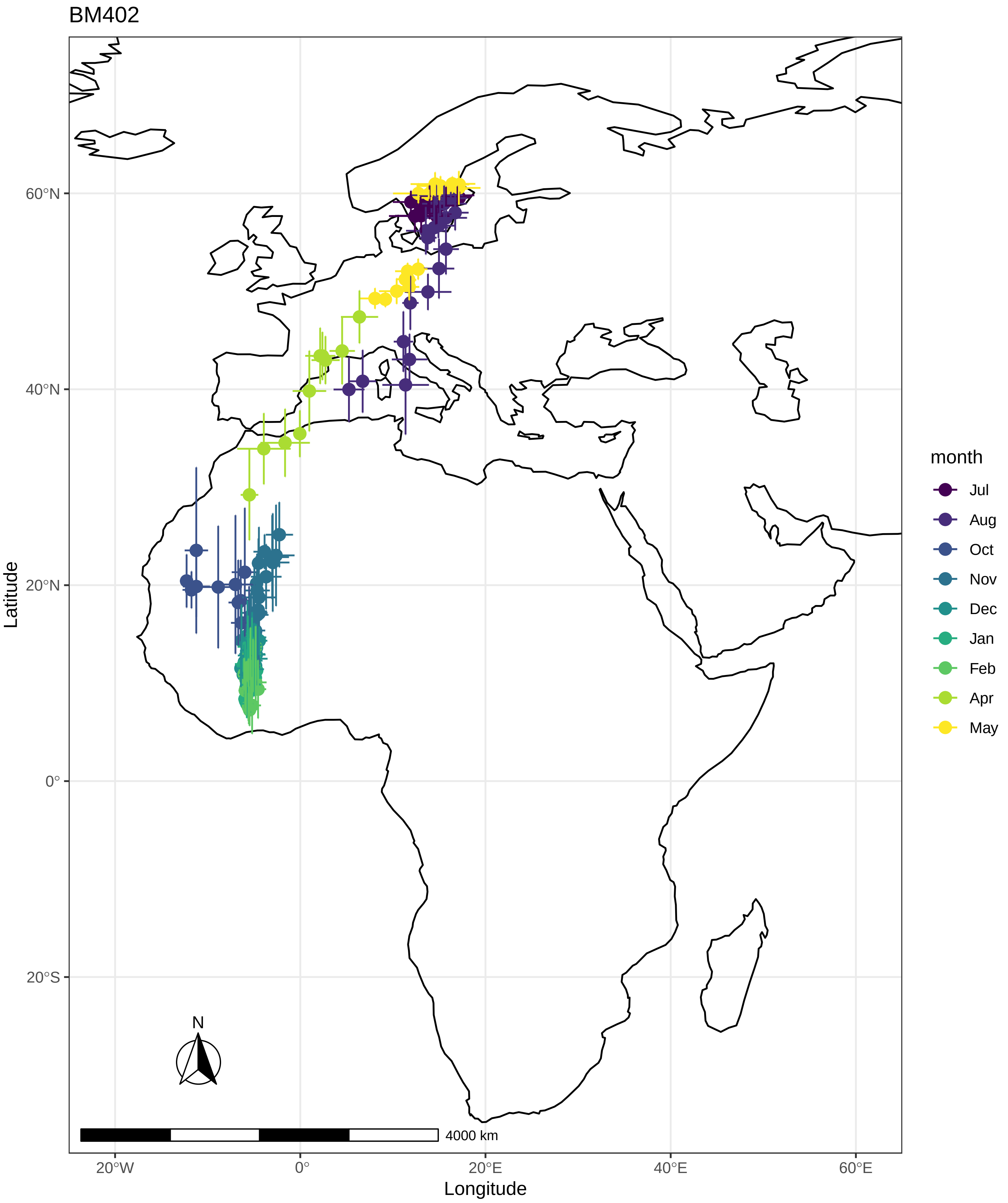

BM402
60°N
40°N
20°N
0°
20°S
Latitude
20°W
0°
20°E
40°E
60°E
Longitude
N
4000 km
month
Jul
Aug
Oct
Nov
Dec
Jan
Feb
Apr
May

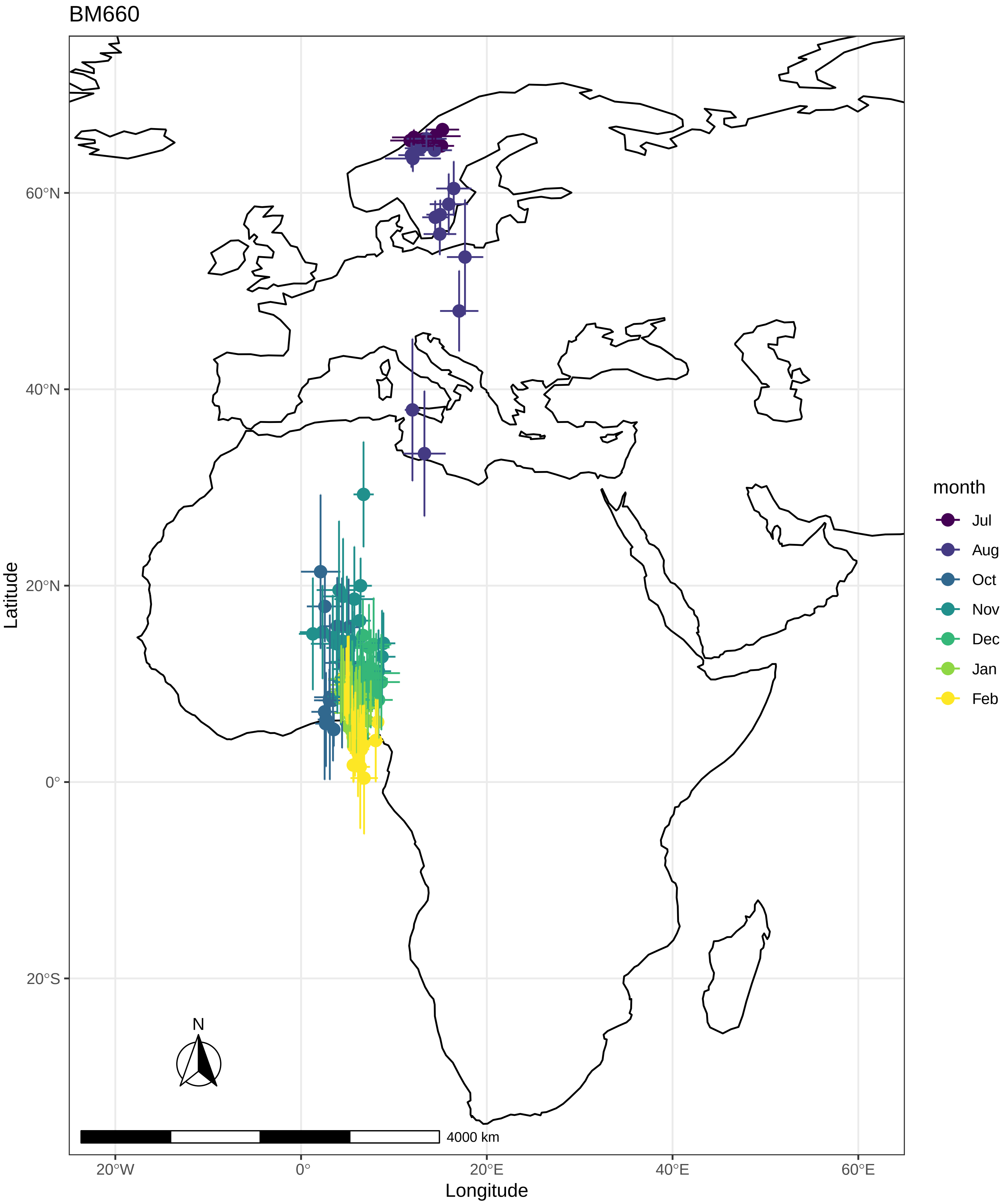
BM660
60°N
40°N
20°N
0°
20°S
Latitude
20°W
0°
20°E
40°E
60°E
Longitude
N
4000 km
month
Jul
Aug
Oct
Nov
Dec
Jan
Feb

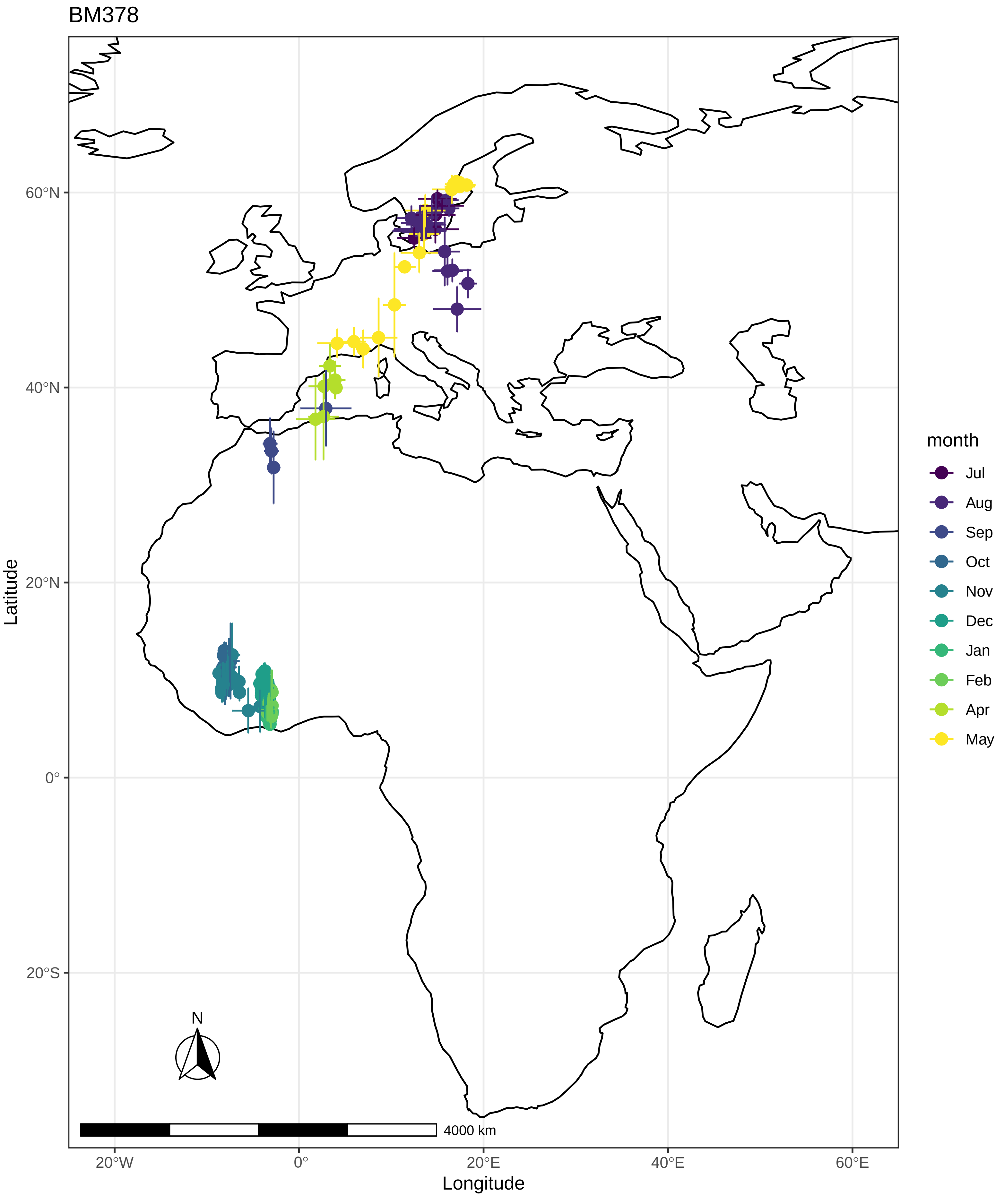
BM378
60°N
40°N
20°N
0°
20°S
Latitude
20°W
0°
20°E
40°E
60°E
Longitude
N
4000 km
month
Jul
Aug
Sep
Oct
Nov
Dec
Jan
Feb
Apr
May

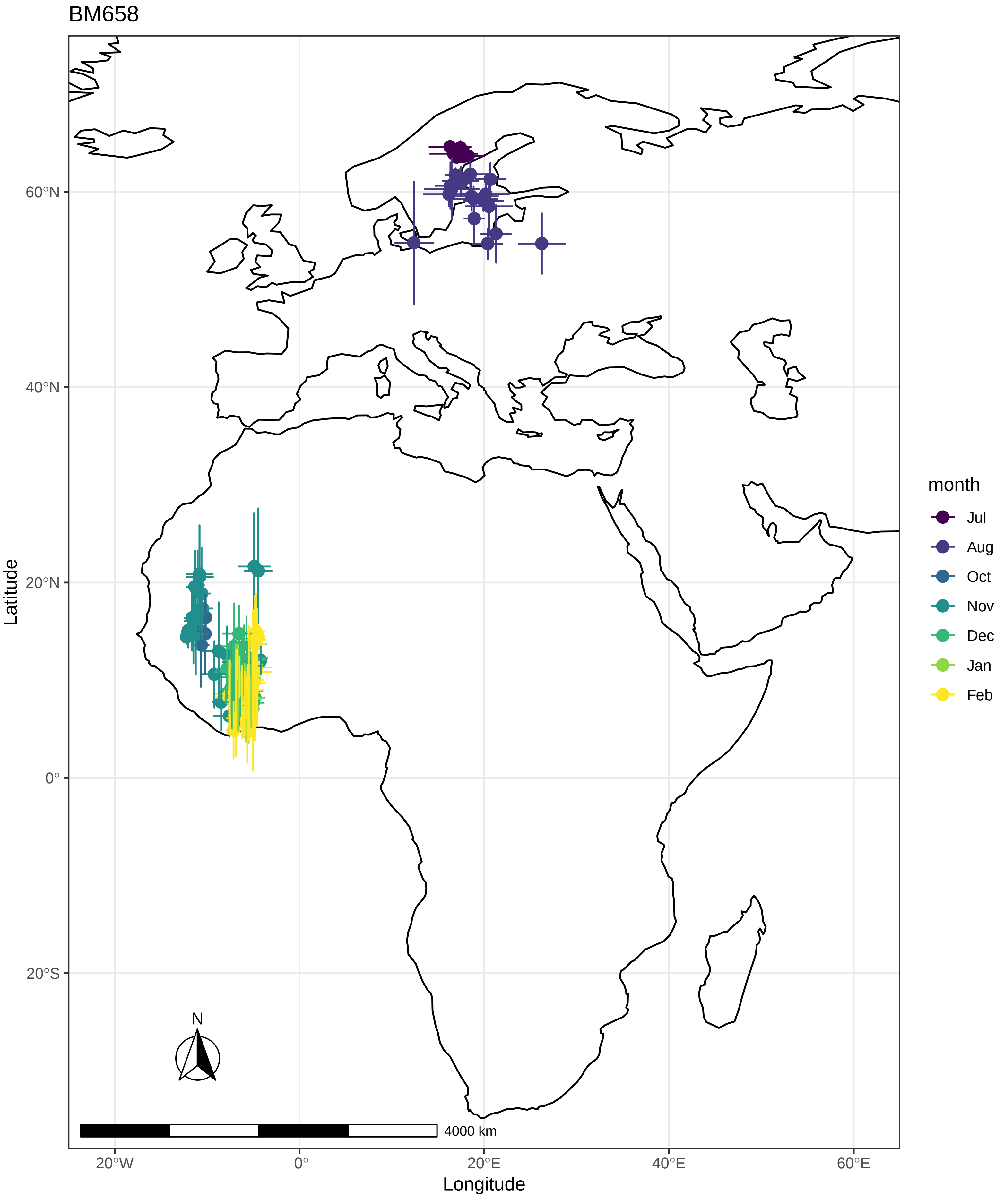
BM658
60°N
40°N
20°N
0°
20°S
Latitude
20°W
0°
20°E
40°E
60°E
Longitude
N
4000 km
month
Jul
Aug
Oct
Nov
Dec
Jan
Feb

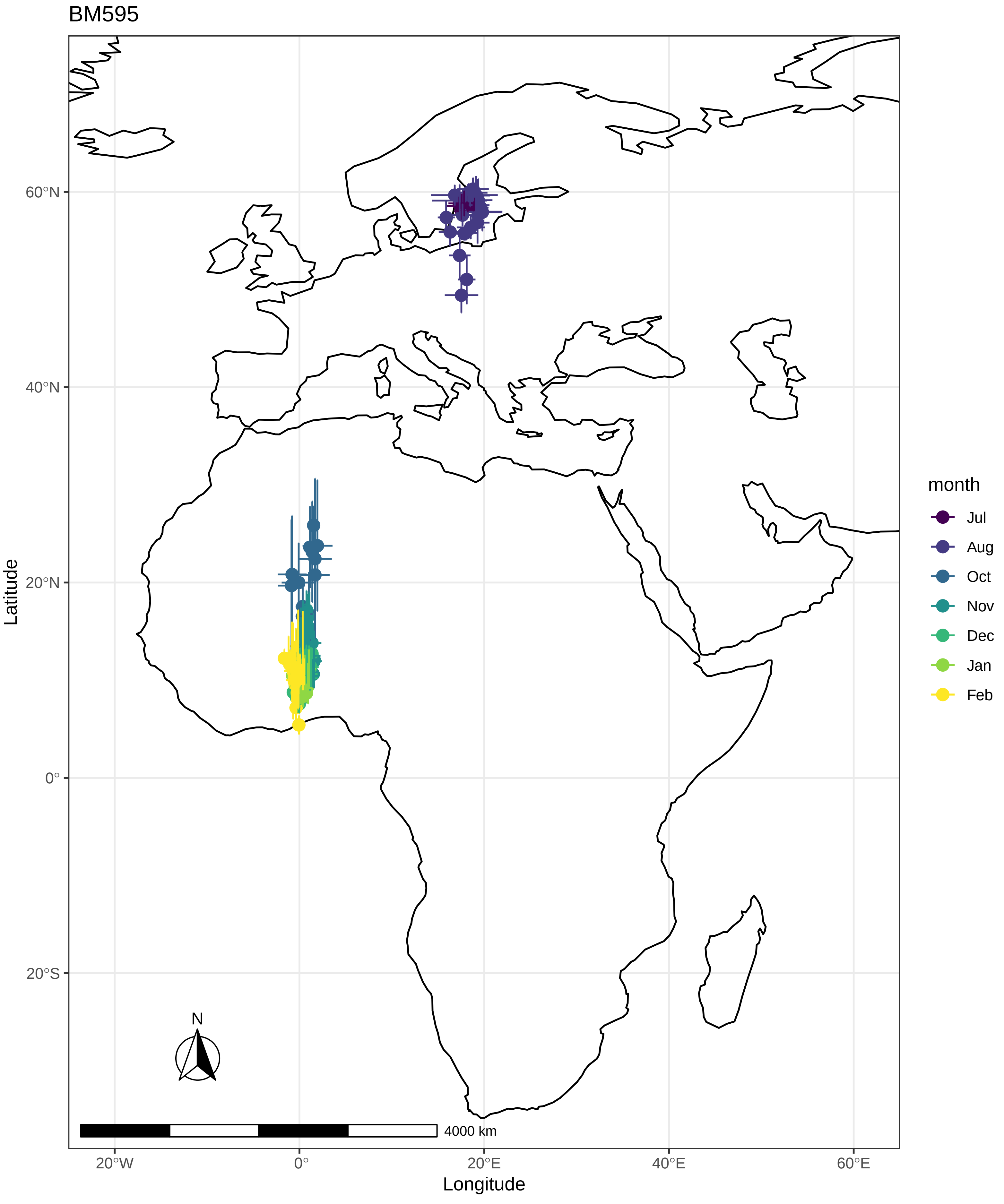

BM595
60°N
40°N
20°N
0°
20°S
Latitude
20°W
0°
20°E
40°E
60°E
Longitude
N
4000 km
month
Jul
Aug
Oct
Nov
Dec
Jan
Feb

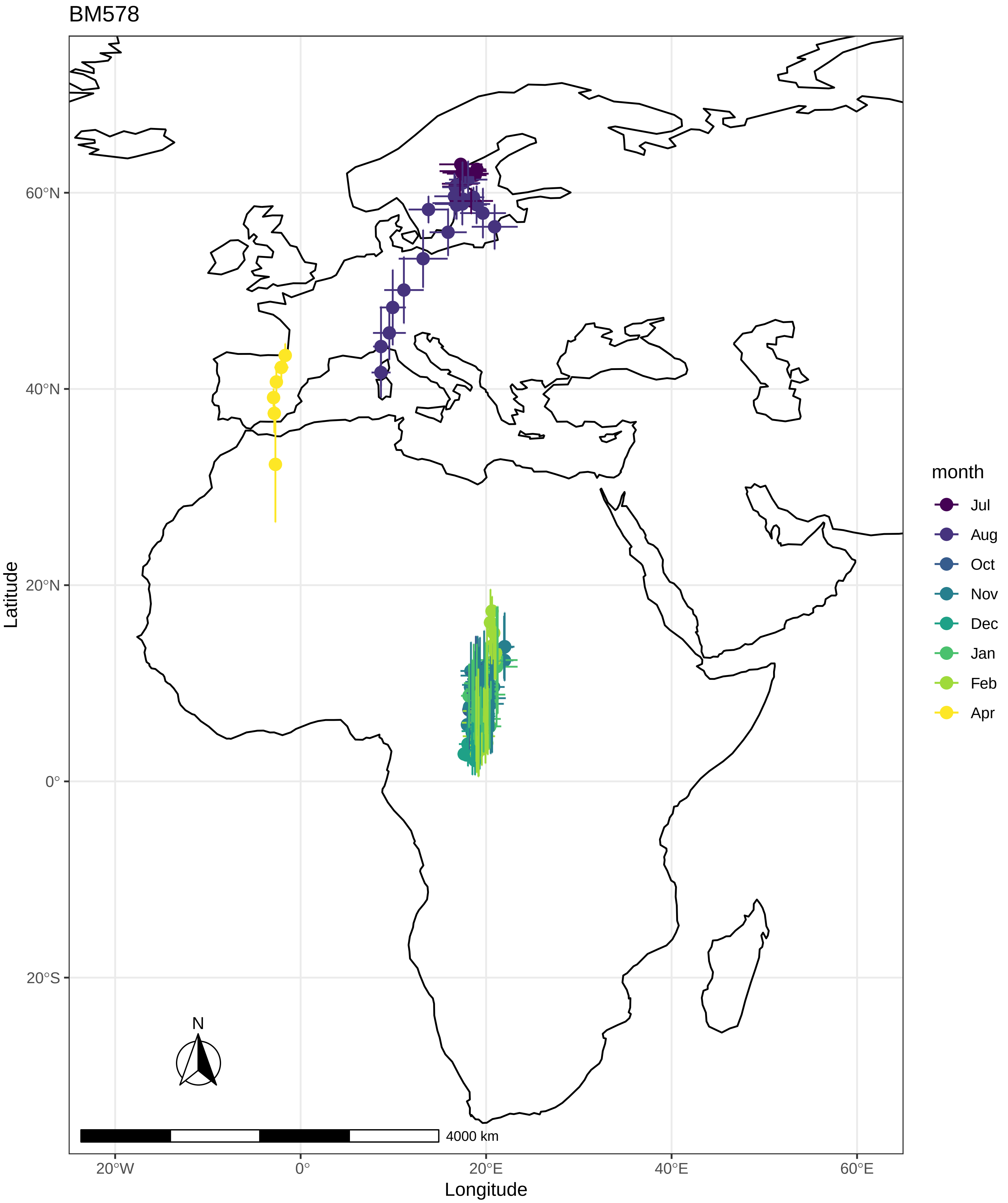
BM578
month
Jul
Aug
Oct
Nov
Dec
Jan
Feb
Apr
60°N
40°N
20°N
0°
20°S
20°W
0°
20°E
40°E
60°E
Latitude
Longitude
N
4000 km

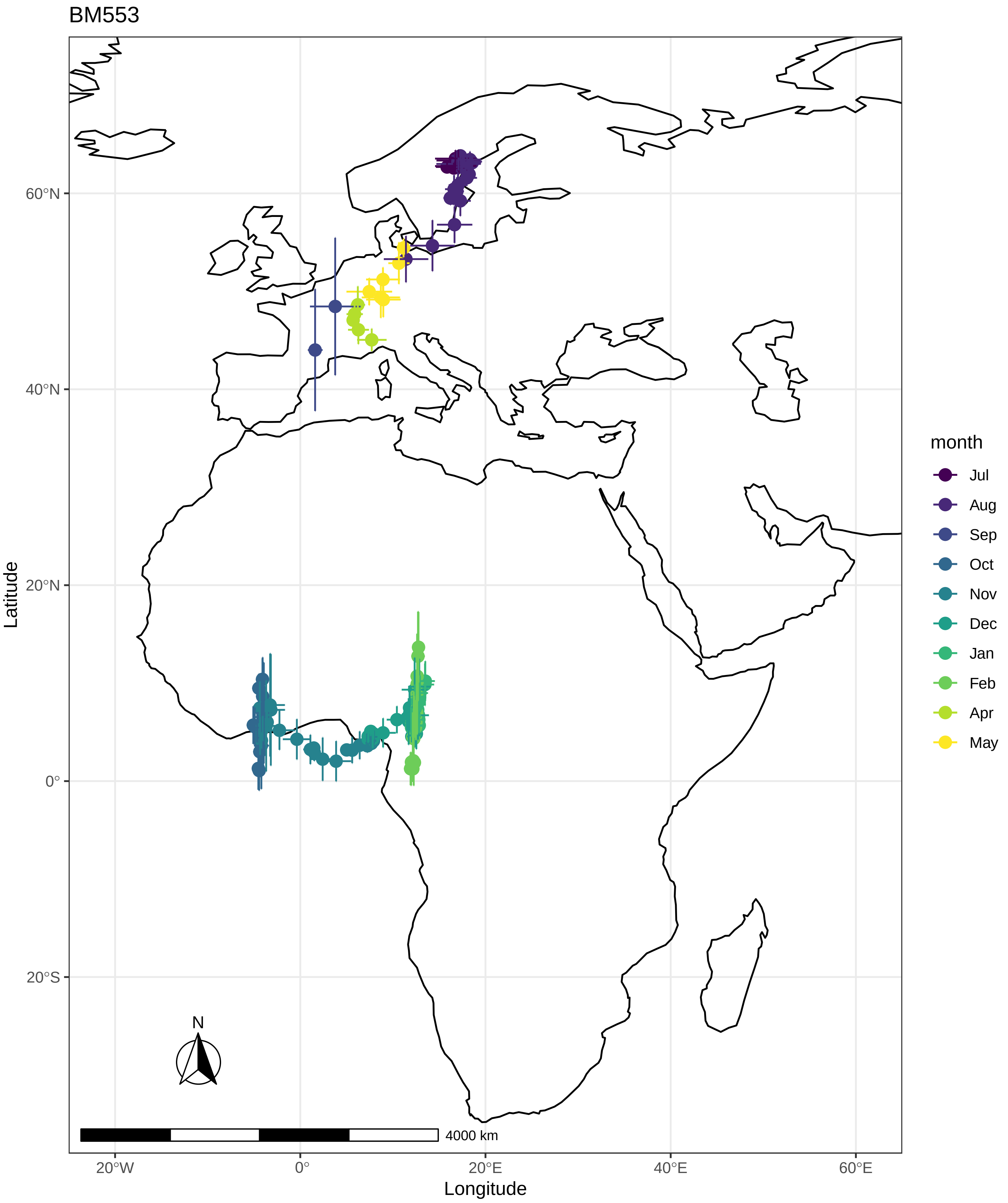

BM553
60°N
40°N
20°N
0°
20°S
Latitude
20°W
0°
20°E
40°E
60°E
Longitude
month
Jul
Aug
Sep
Oct
Nov
Dec
Jan
Feb
Apr
May
N
4000 km

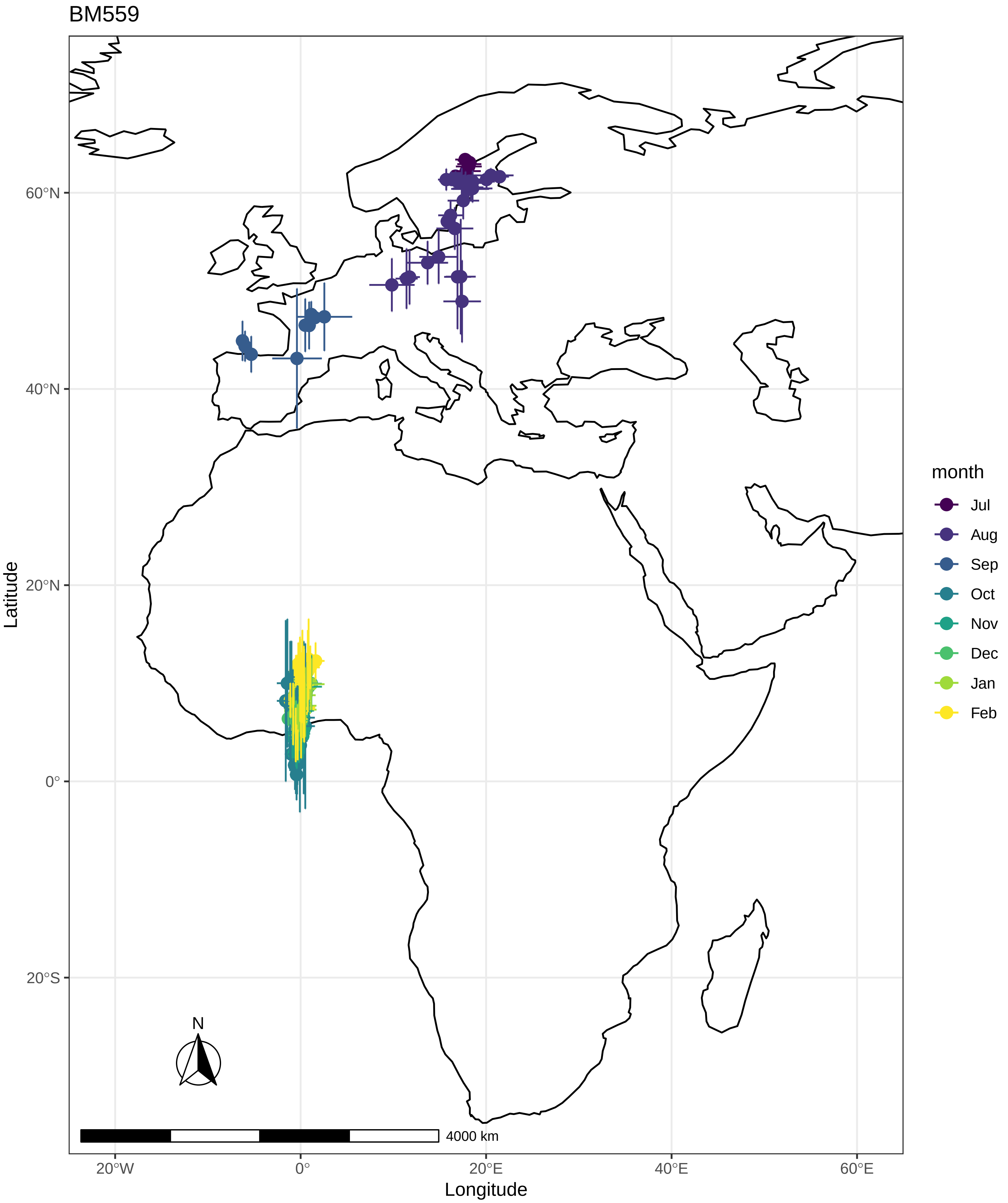

BM559
60°N
40°N
20°N
0°
20°S
Latitude
20°W
0°
20°E
40°E
60°E
Longitude
N
4000 km
month
Jul
Aug
Sep
Oct
Nov
Dec
Jan
Feb

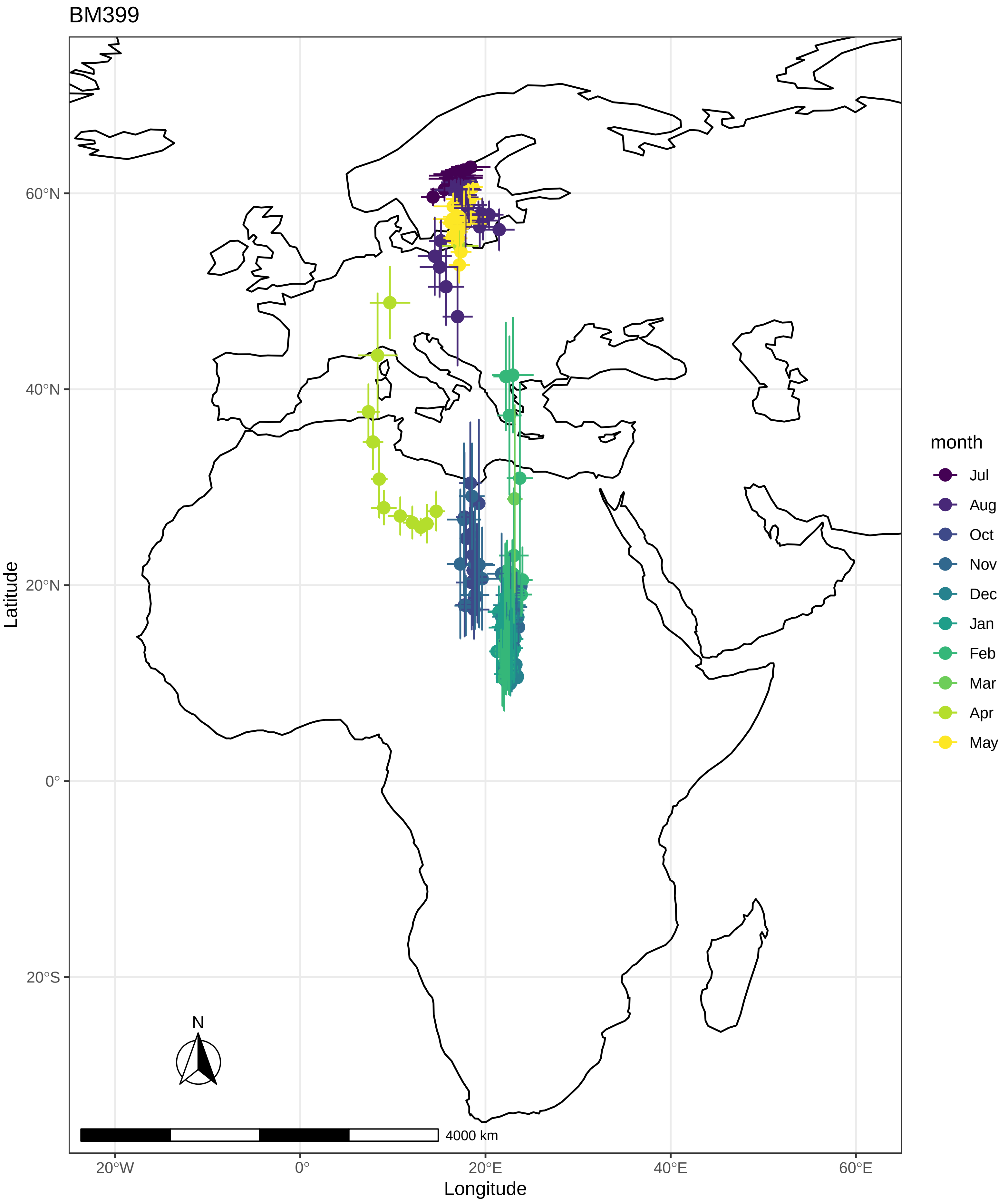
BM399
60°N
40°N
20°N
0°
20°S
Latitude
20°W
0°
20°E
40°E
60°E
Longitude
N
4000 km
month
Jul
Aug
Oct
Nov
Dec
Jan
Feb
Mar
Apr
May

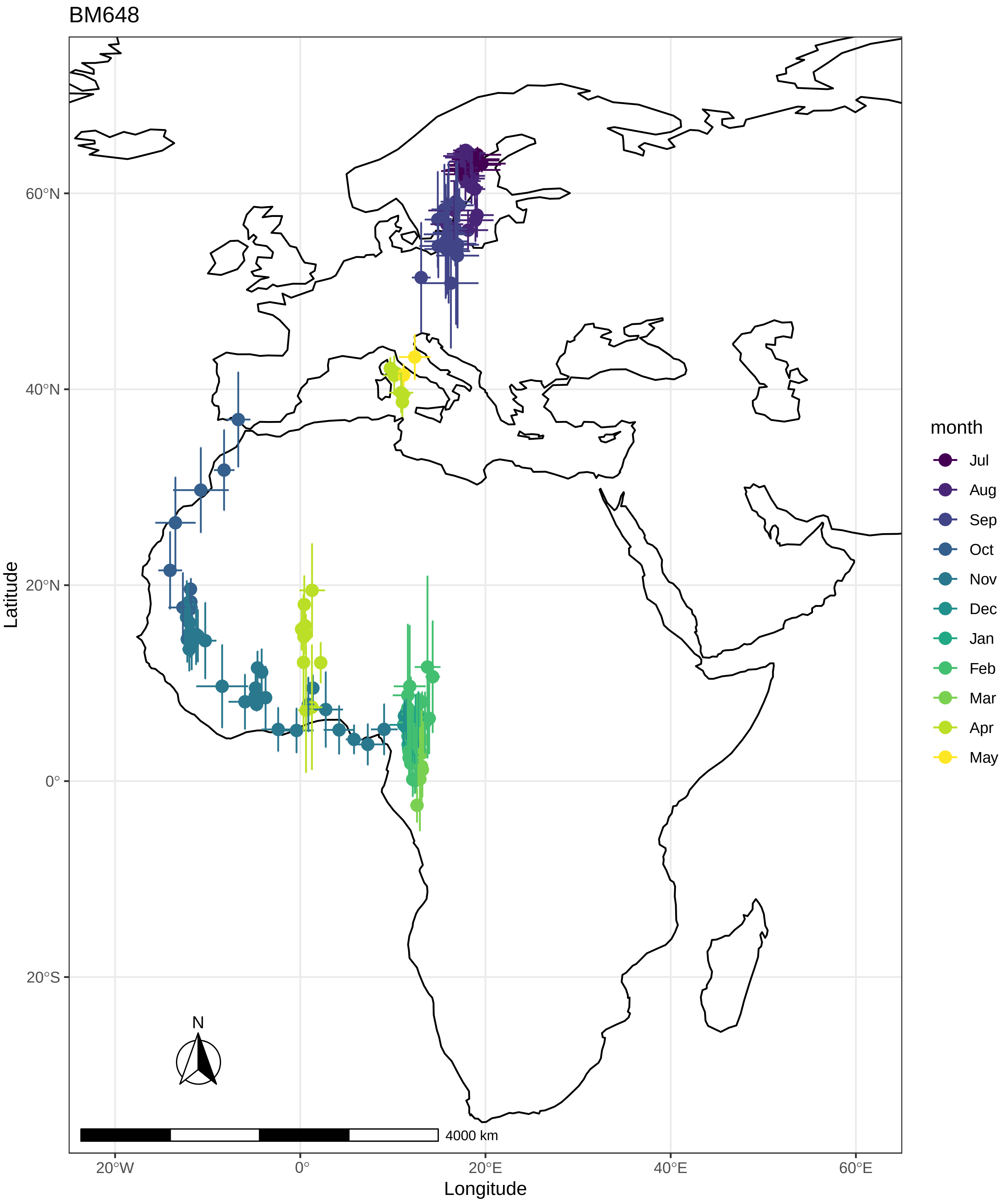

BM648
60°N
40°N
20°N
0°
20°S
20°W
0°
20°E
40°E
60°E
Latitude
Longitude
N
4000 km
month
Jul
Aug
Sep
Oct
Nov
Dec
Jan
Feb
Mar
Apr
May

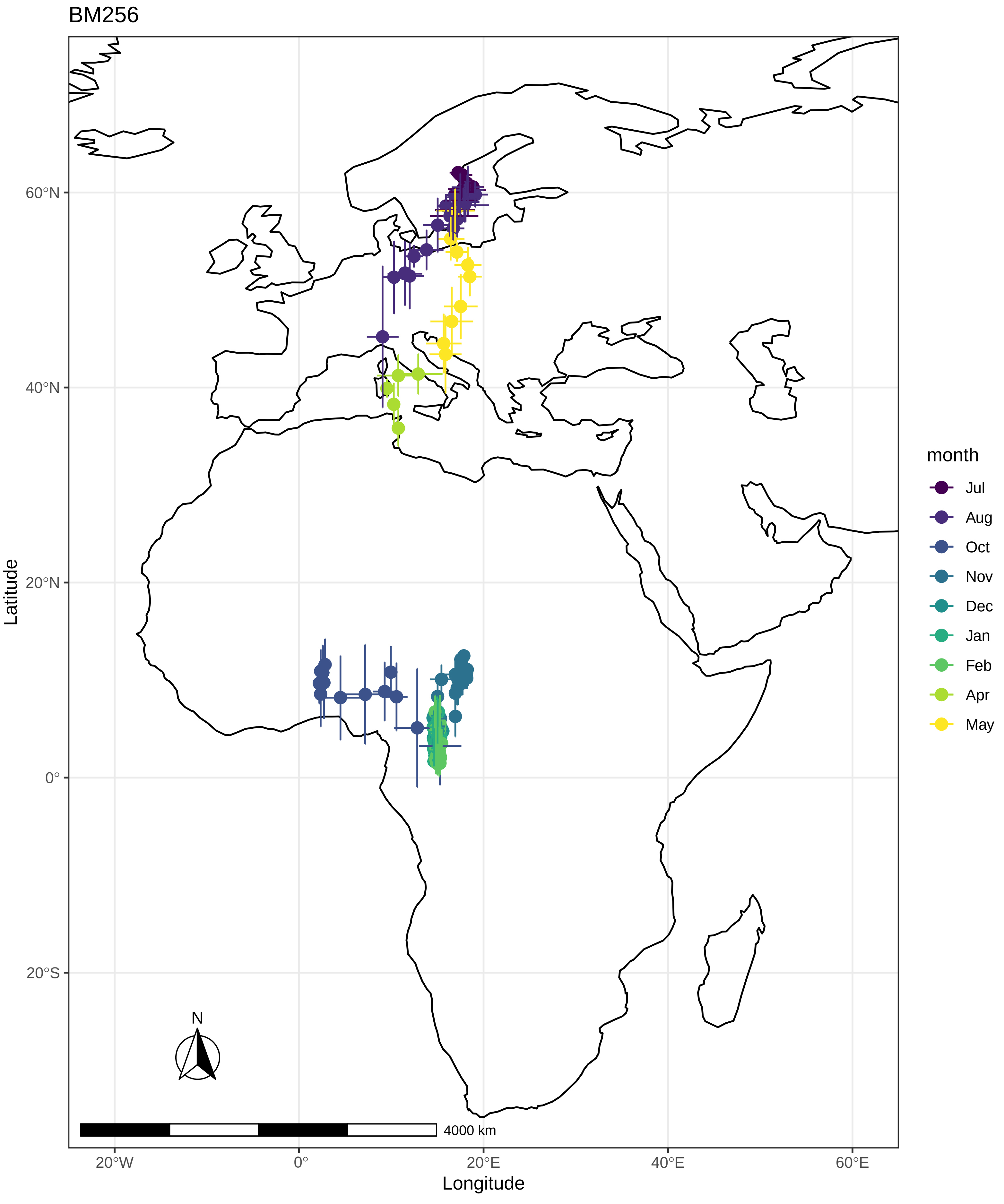

BM256
60°N
40°N
20°N
0°
20°S
Latitude
20°W
0°
20°E
40°E
60°E
Longitude
N
4000 km
month
Jul
Aug
Oct
Nov
Dec
Jan
Feb
Apr
May

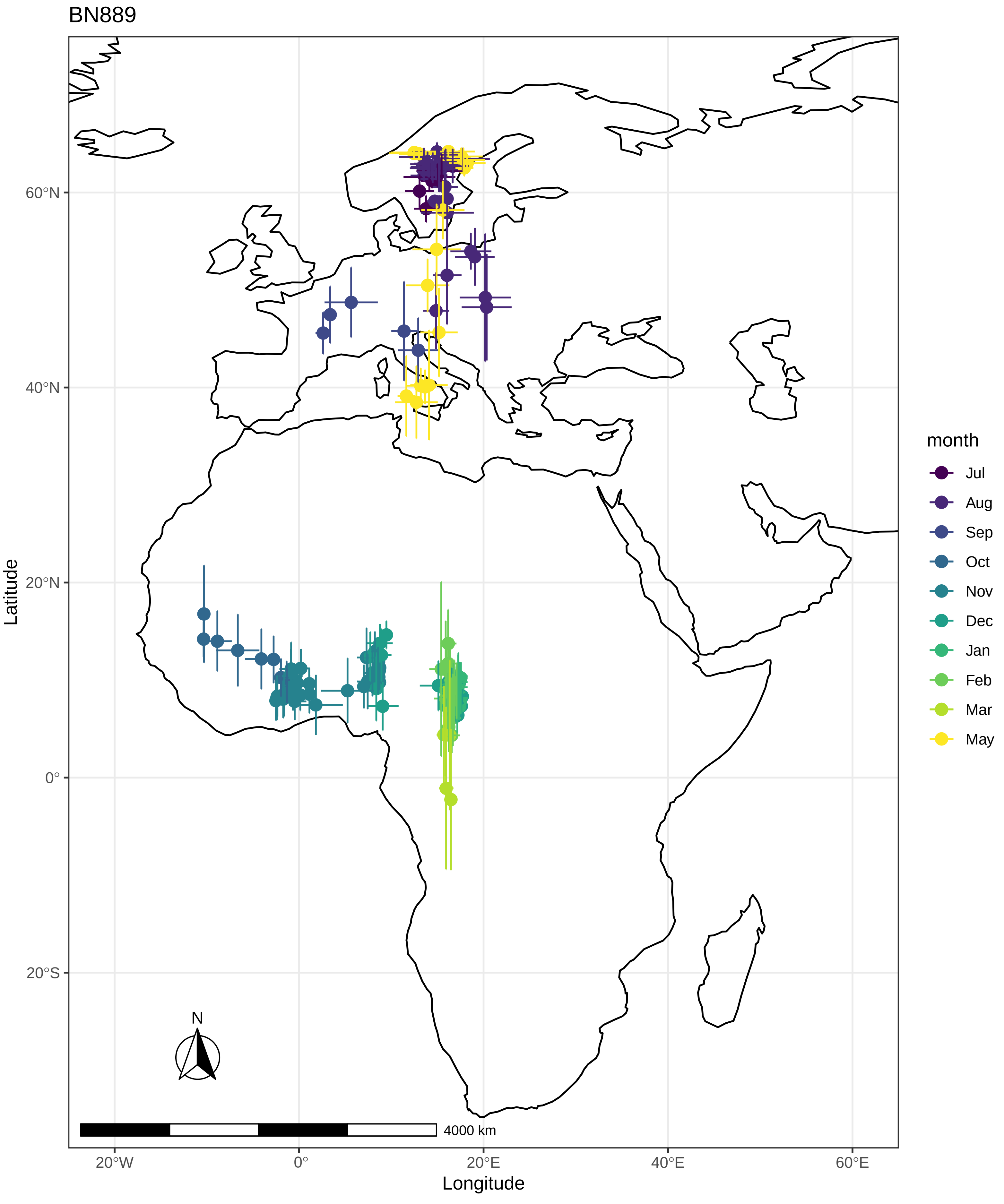

BN889
60°N
40°N
20°N
0°
20°S
20°W
0°
20°E
40°E
60°E
Latitude
Longitude
N
4000 km
month
Jul
Aug
Sep
Oct
Nov
Dec
Jan
Feb
Mar
May

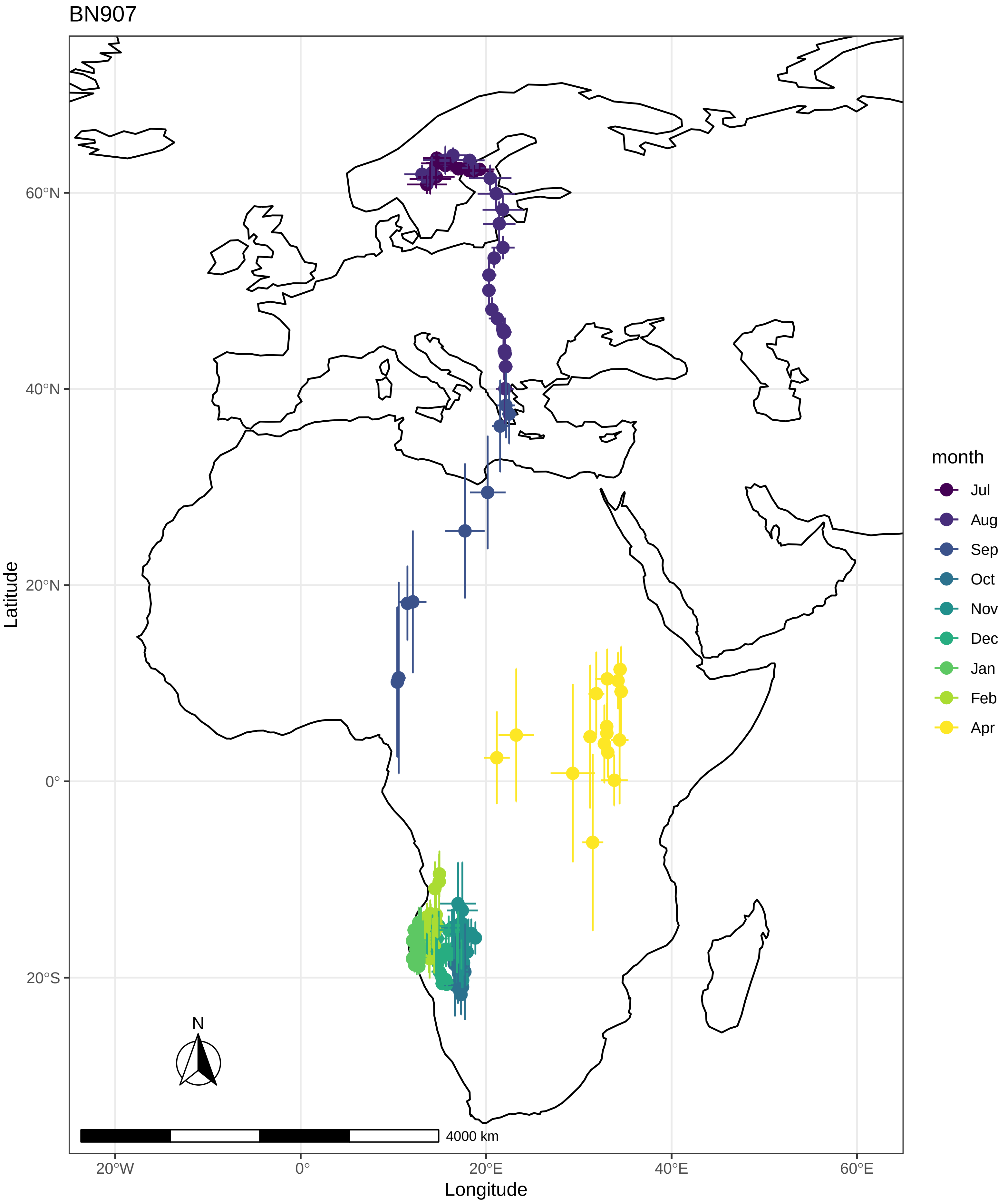

BN907
60°N
40°N
20°N
0°
20°S
Latitude
20°W
0°
20°E
40°E
60°E
Longitude
N
4000 km
month
Jul
Aug
Sep
Oct
Nov
Dec
Jan
Feb
Apr

BN886

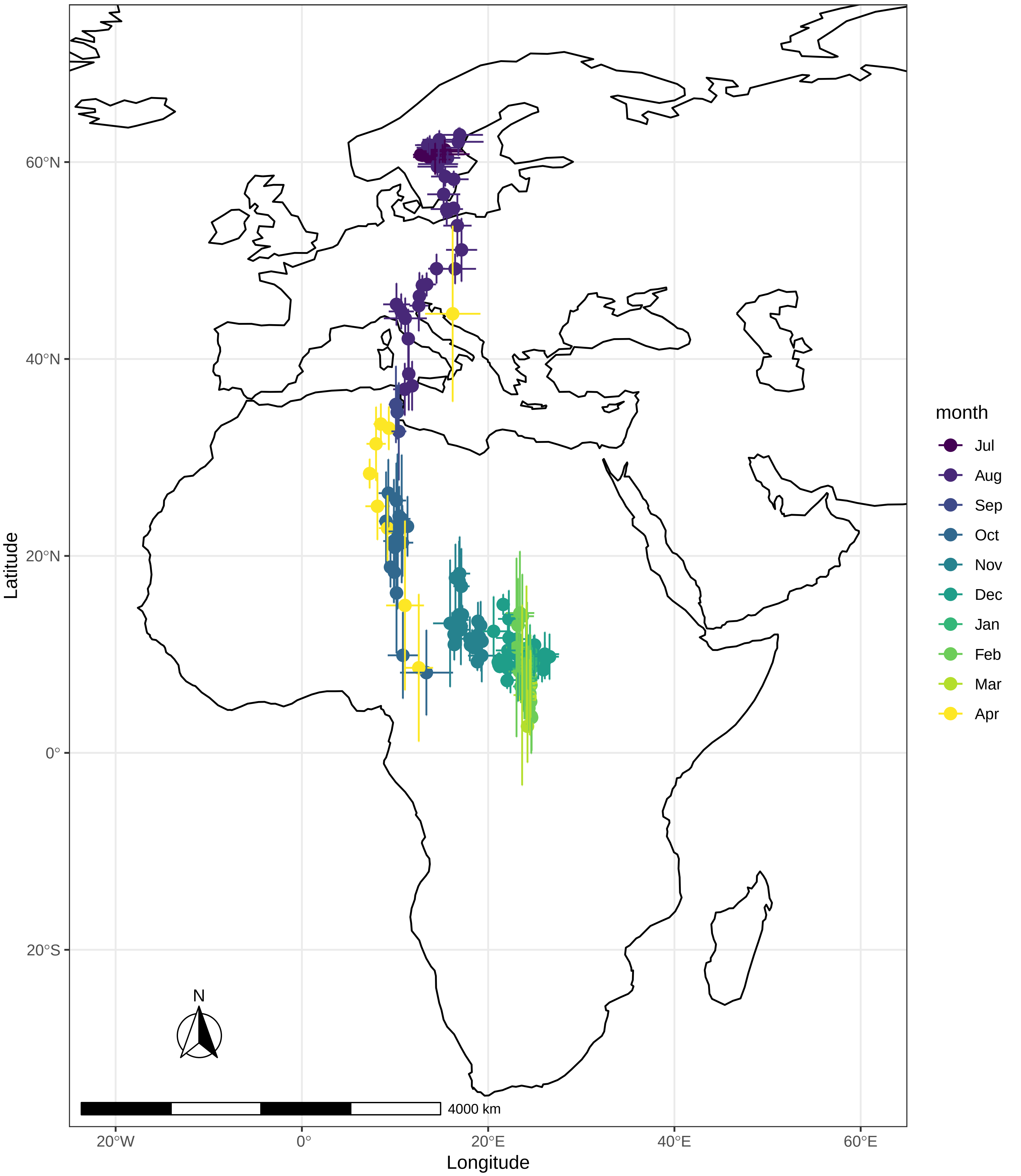

60°N
40°N
20°N
0°
20°S
Latitude
20°W
0°
20°E
40°E
60°E
Longitude
N
4000 km
month
Jul
Aug
Sep
Oct
Nov
Dec
Jan
Feb
Mar
Apr

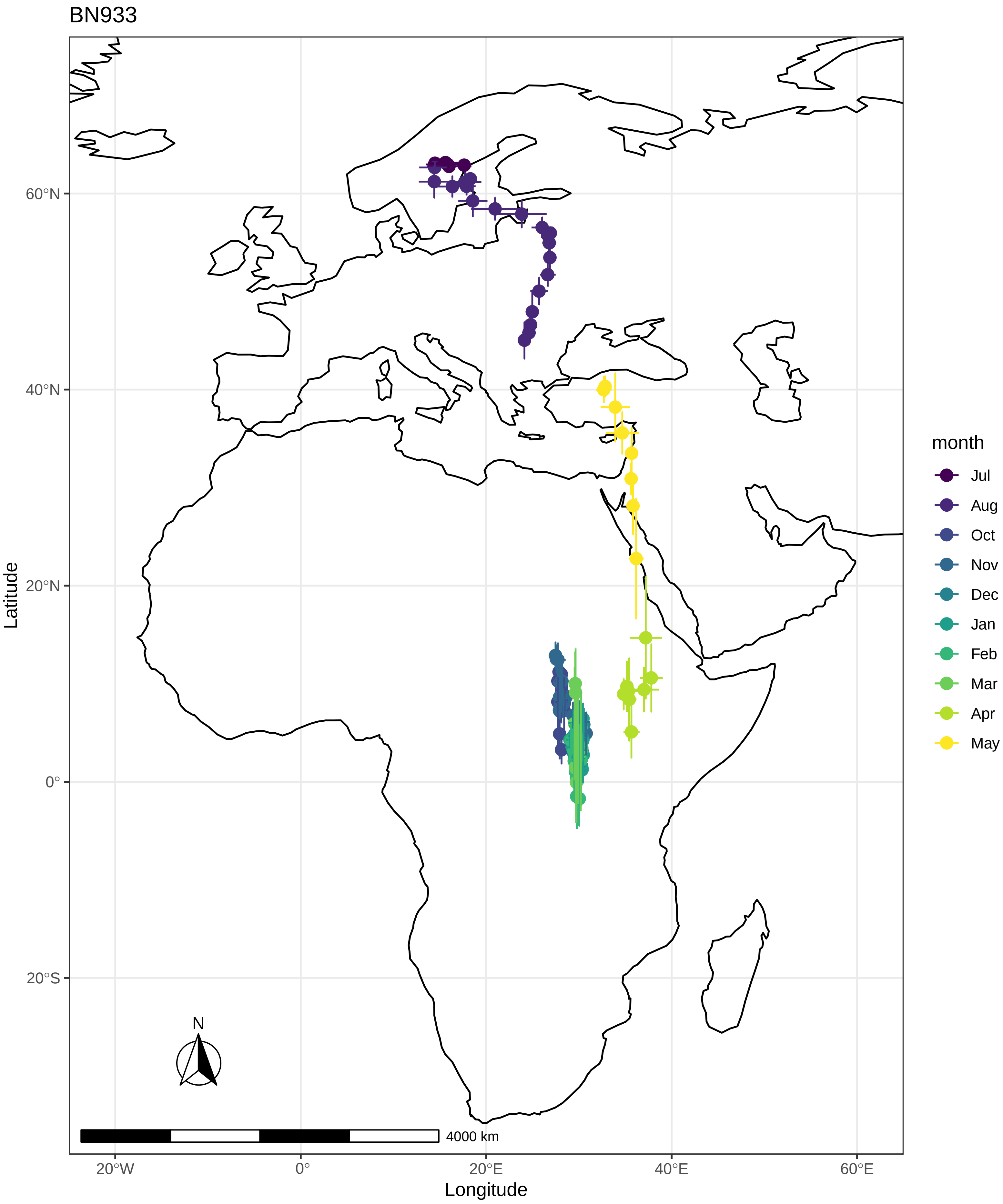

BN933
60°N
40°N
20°N
0°
20°S
Latitude
20°W
0°
20°E
40°E
60°E
Longitude
N
4000 km
month
Jul
Aug
Oct
Nov
Dec
Jan
Feb
Mar
Apr
May

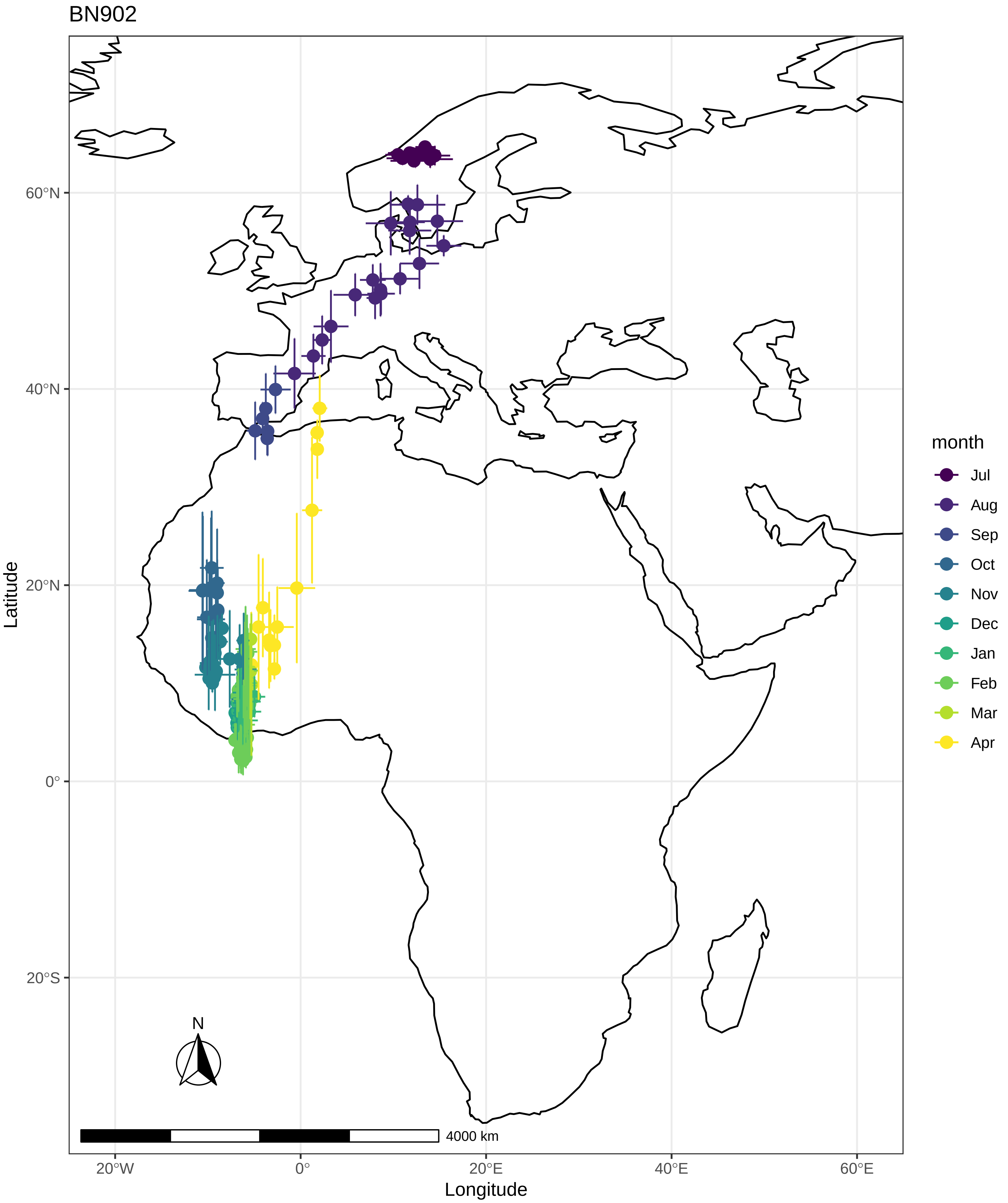

BN902
60°N
40°N
20°N
0°
20°S
20°W
0°
20°E
40°E
60°E
Latitude
Longitude
N
4000 km
month
Jul
Aug
Sep
Oct
Nov
Dec
Jan
Feb
Mar
Apr

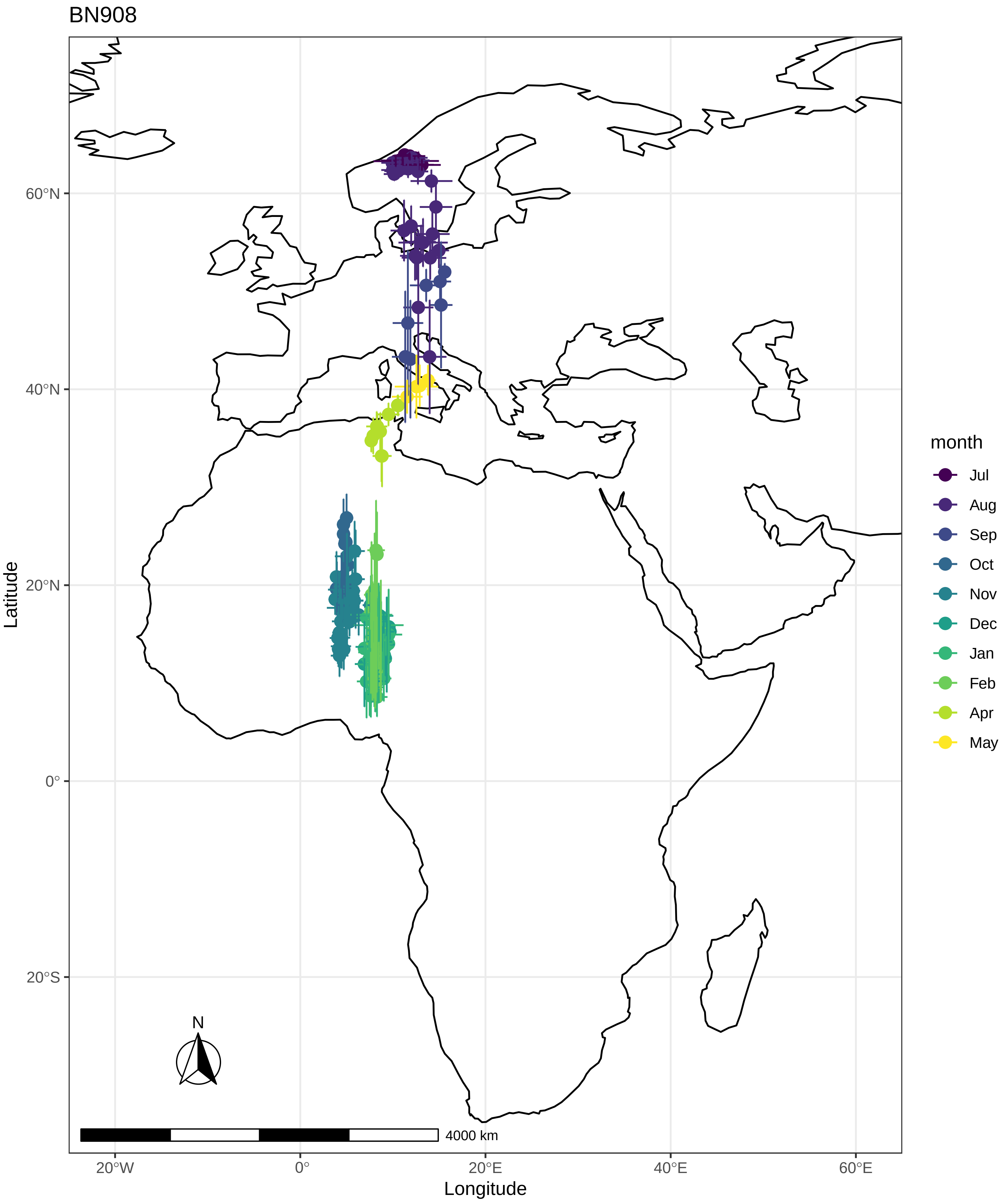

BN908
60°N
40°N
20°N
0°
20°S
Latitude
20°W
0°
20°E
40°E
60°E
Longitude
N
4000 km
month
Jul
Aug
Sep
Oct
Nov
Dec
Jan
Feb
Apr
May

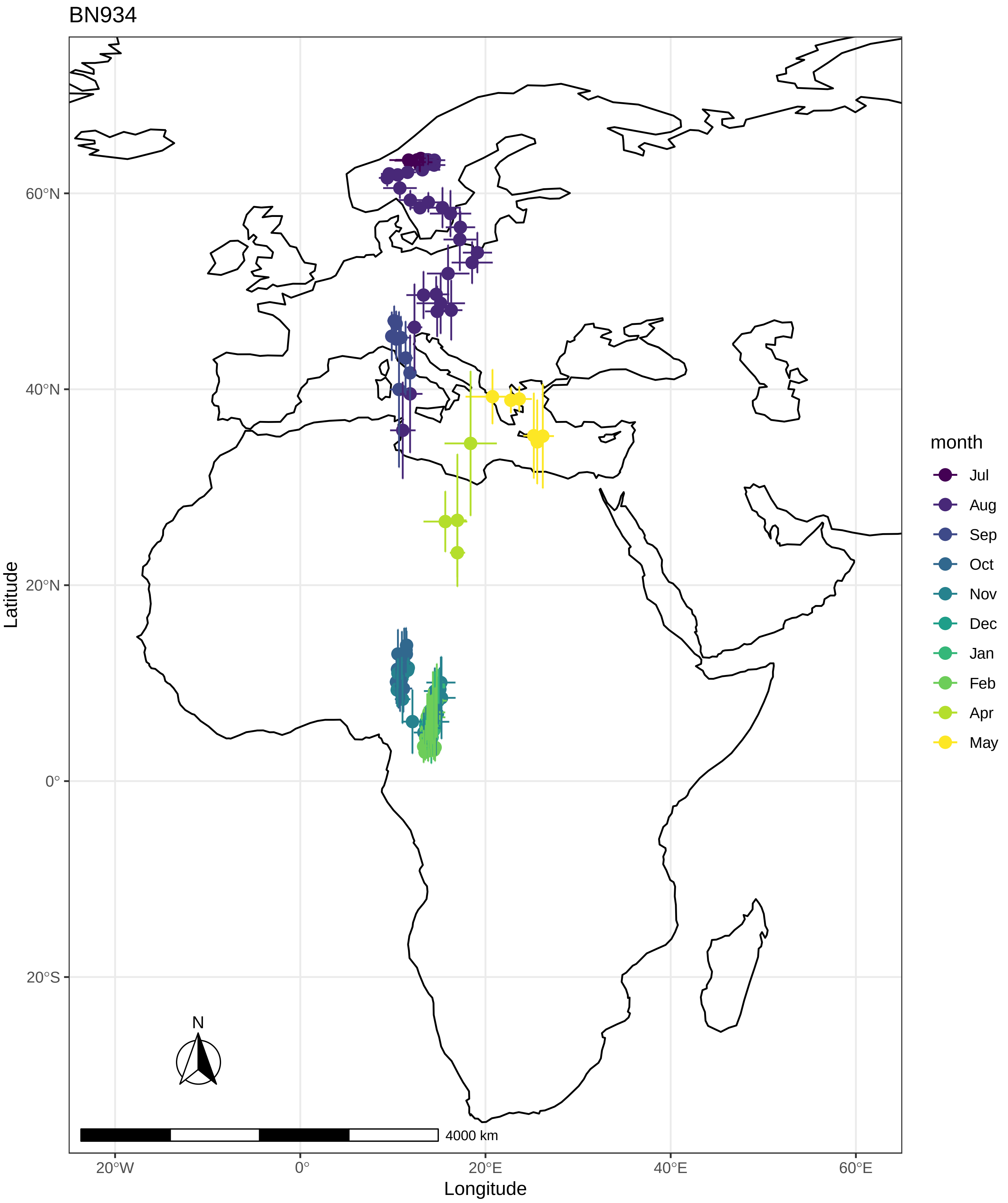

BN934
month
Jul
Aug
Sep
Oct
Nov
Dec
Jan
Feb
Apr
May
60°N
40°N
20°N
0°
20°S
Latitude
20°W
0°
20°E
40°E
60°E
Longitude
N
4000 km

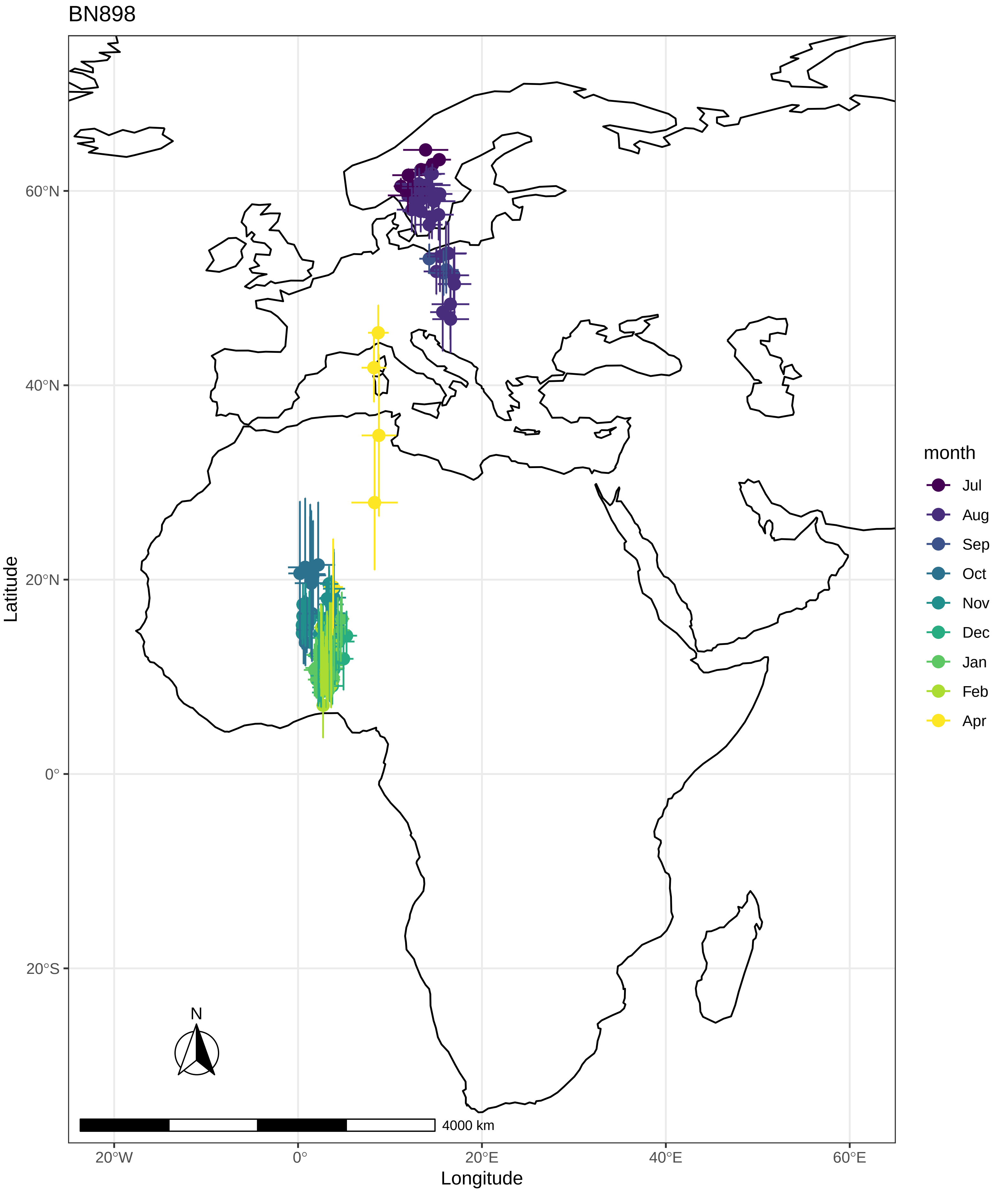
BN898
60°N
40°N
20°N
0°
20°S
Latitude
20°W
0°
20°E
40°E
60°E
Longitude
N
4000 km
month
Jul
Aug
Sep
Oct
Nov
Dec
Jan
Feb
Apr

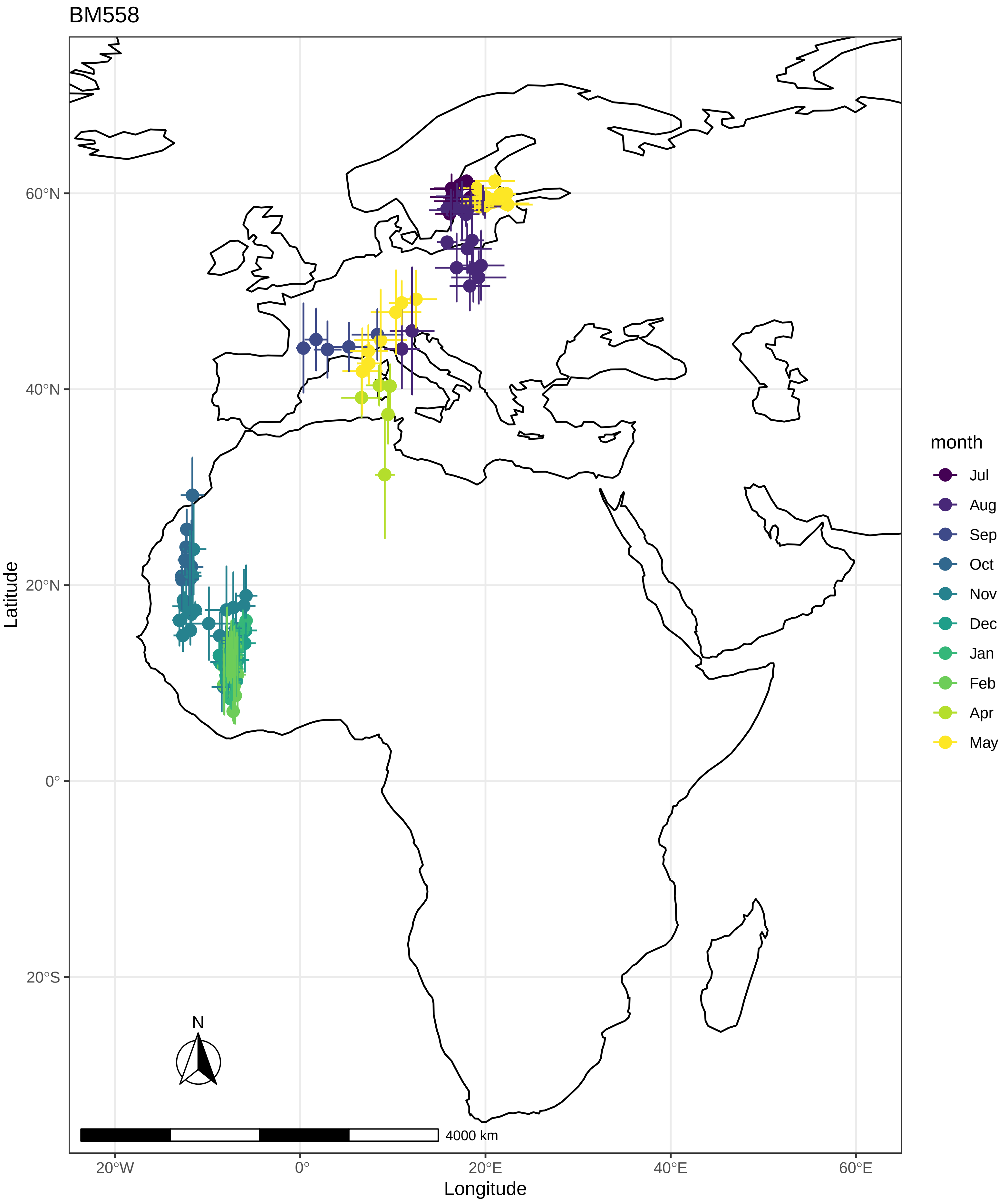

BM558
60°N
40°N
20°N
0°
20°S
20°W
0°
20°E
40°E
60°E
Latitude
Longitude
month
Jul
Aug
Sep
Oct
Nov
Dec
Jan
Feb
Apr
May
N
4000 km

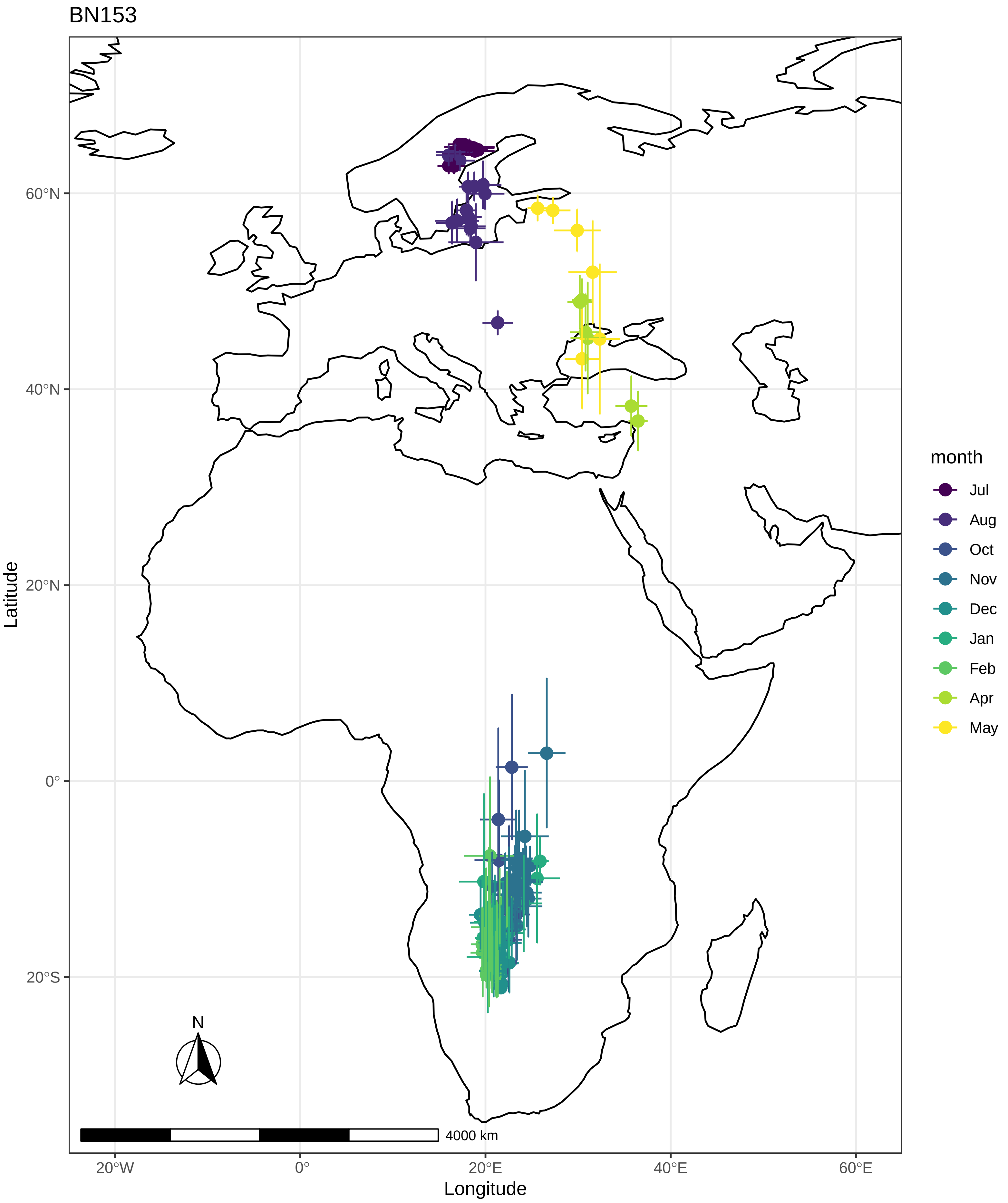
BN153
60°N
40°N
20°N
0°
20°S
Latitude
20°W
0°
20°E
40°E
60°E
Longitude
N
4000 km
month
Jul
Aug
Oct
Nov
Dec
Jan
Feb
Apr
May

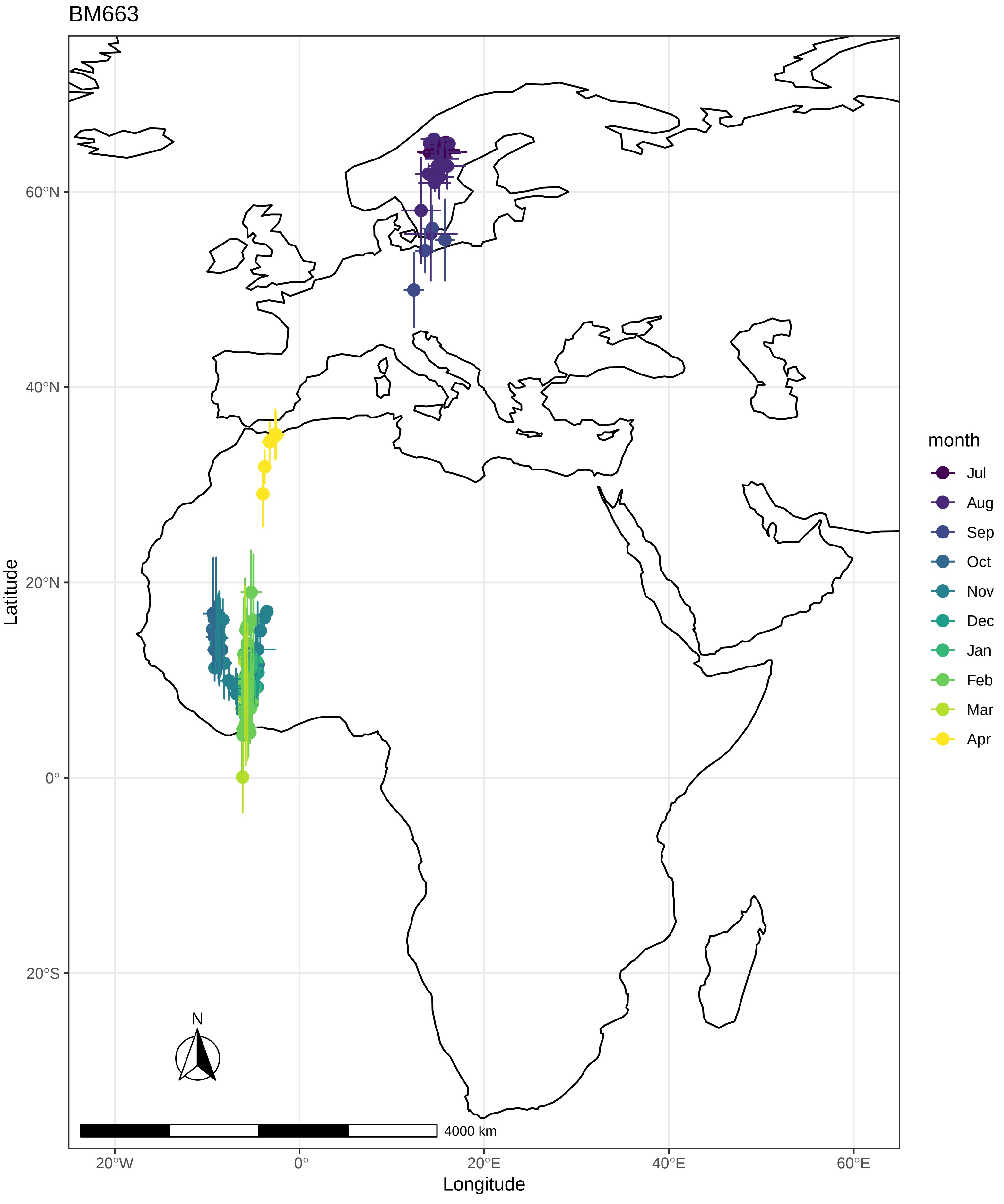

BM663
60°N
40°N
20°N
0°
20°S
20°W
0°
20°E
40°E
60°E
Latitude
Longitude
N
4000 km
month
Jul
Aug
Sep
Oct
Nov
Dec
Jan
Feb
Mar
Apr

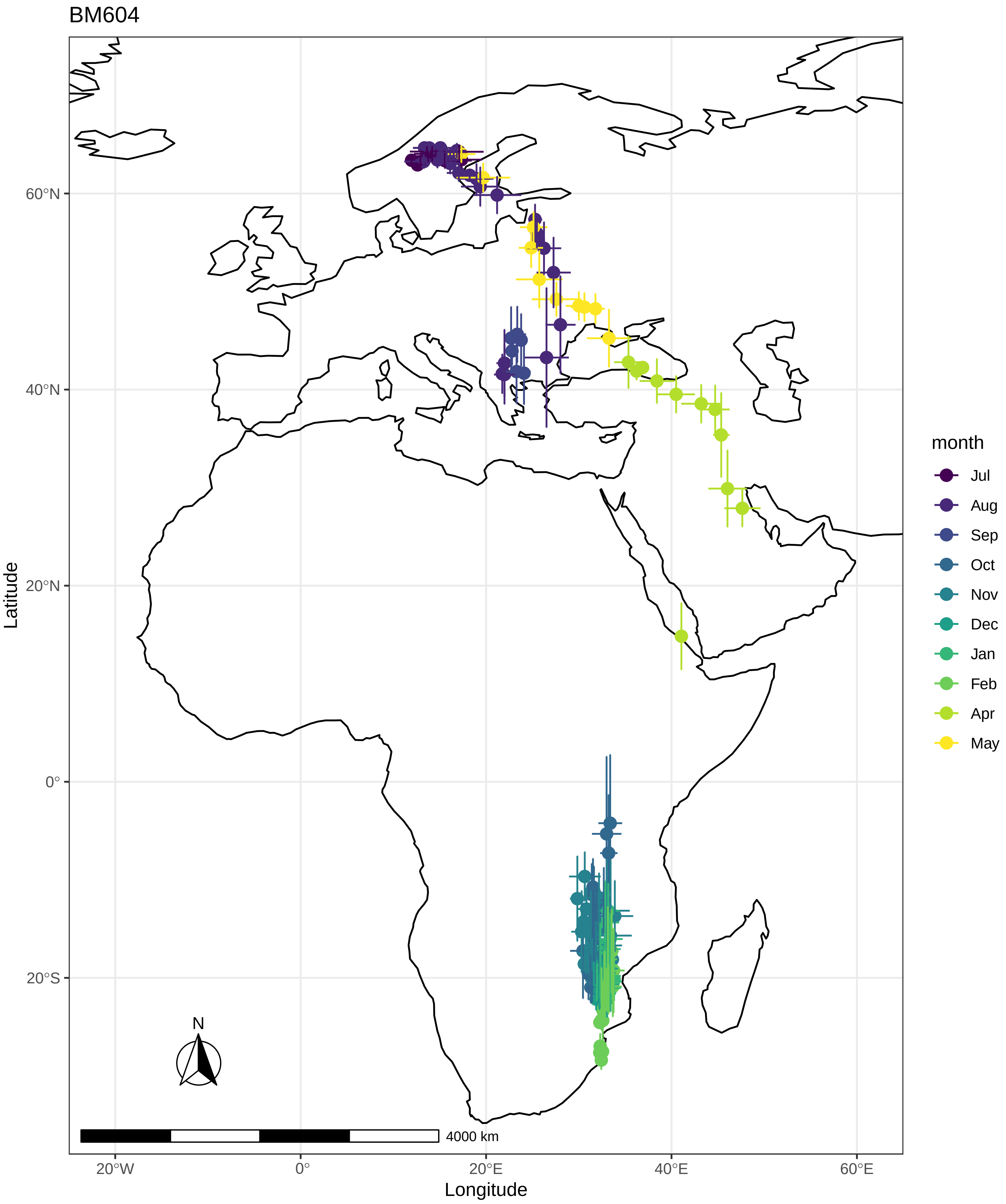
BM604
60°N
40°N
20°N
0°
20°S
Latitude
20°W
0°
20°E
40°E
60°E
Longitude
N
4000 km
month
Jul
Aug
Sep
Oct
Nov
Dec
Jan
Feb
Apr
May

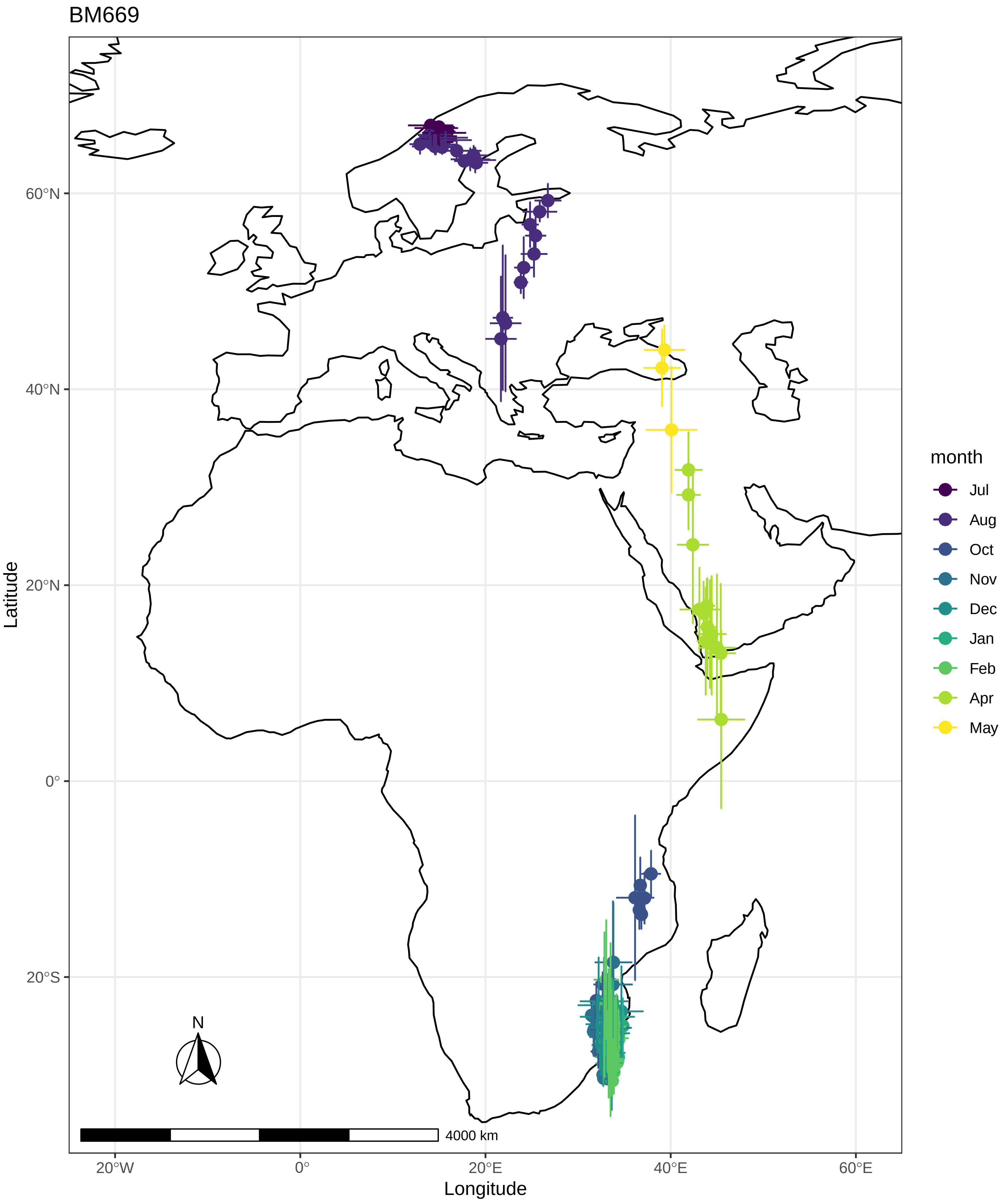
BM669
60°N
40°N
20°N
0°
20°S
Latitude
20°W
0°
20°E
40°E
60°E
Longitude
month
Jul
Aug
Oct
Nov
Dec
Jan
Feb
Apr
May
N
4000 km

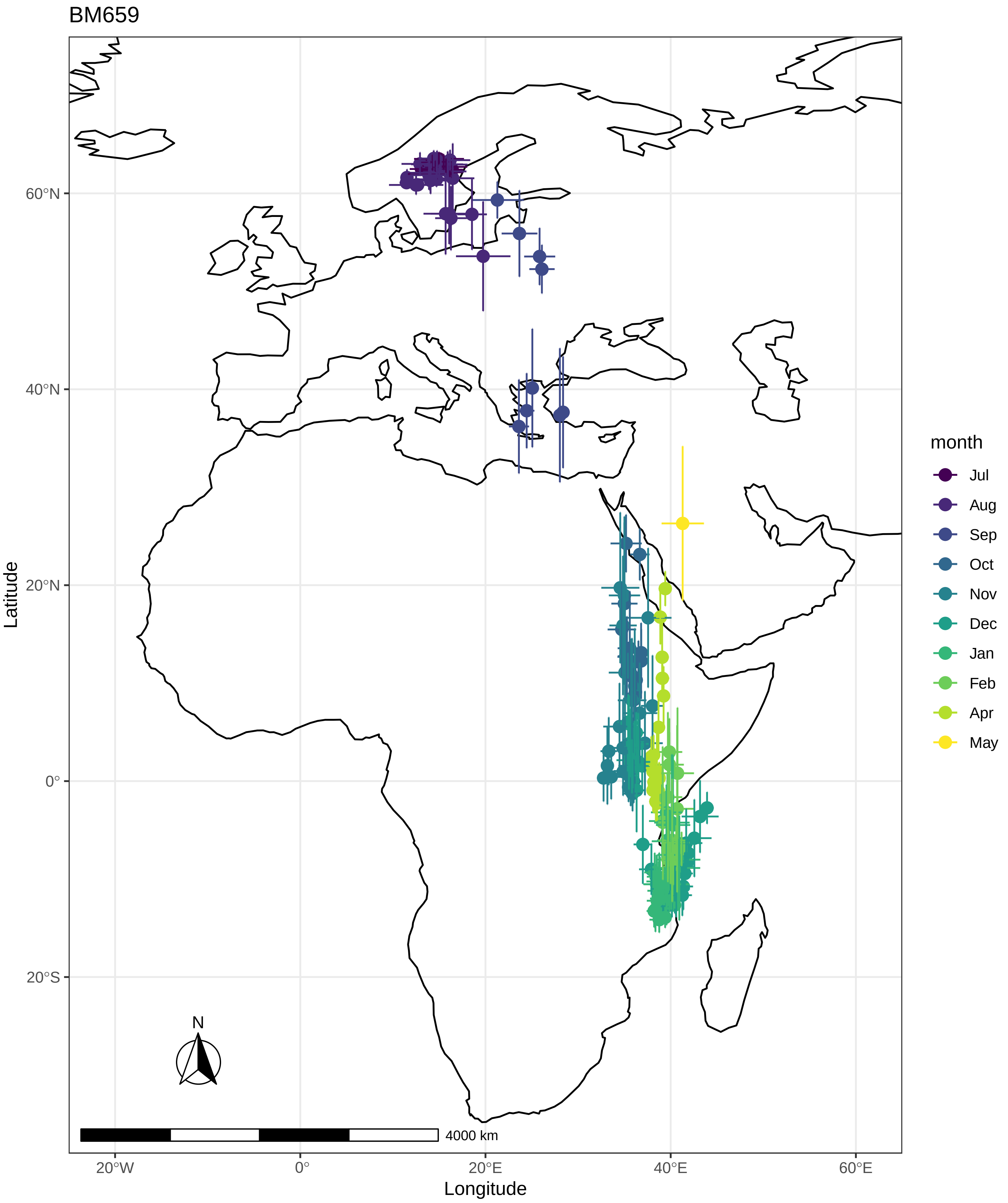

BM659
60°N
40°N
20°N
0°
20°S
20°W
0°
20°E
40°E
60°E
Latitude
Longitude
N
4000 km
month
Jul
Aug
Sep
Oct
Nov
Dec
Jan
Feb
Apr
May

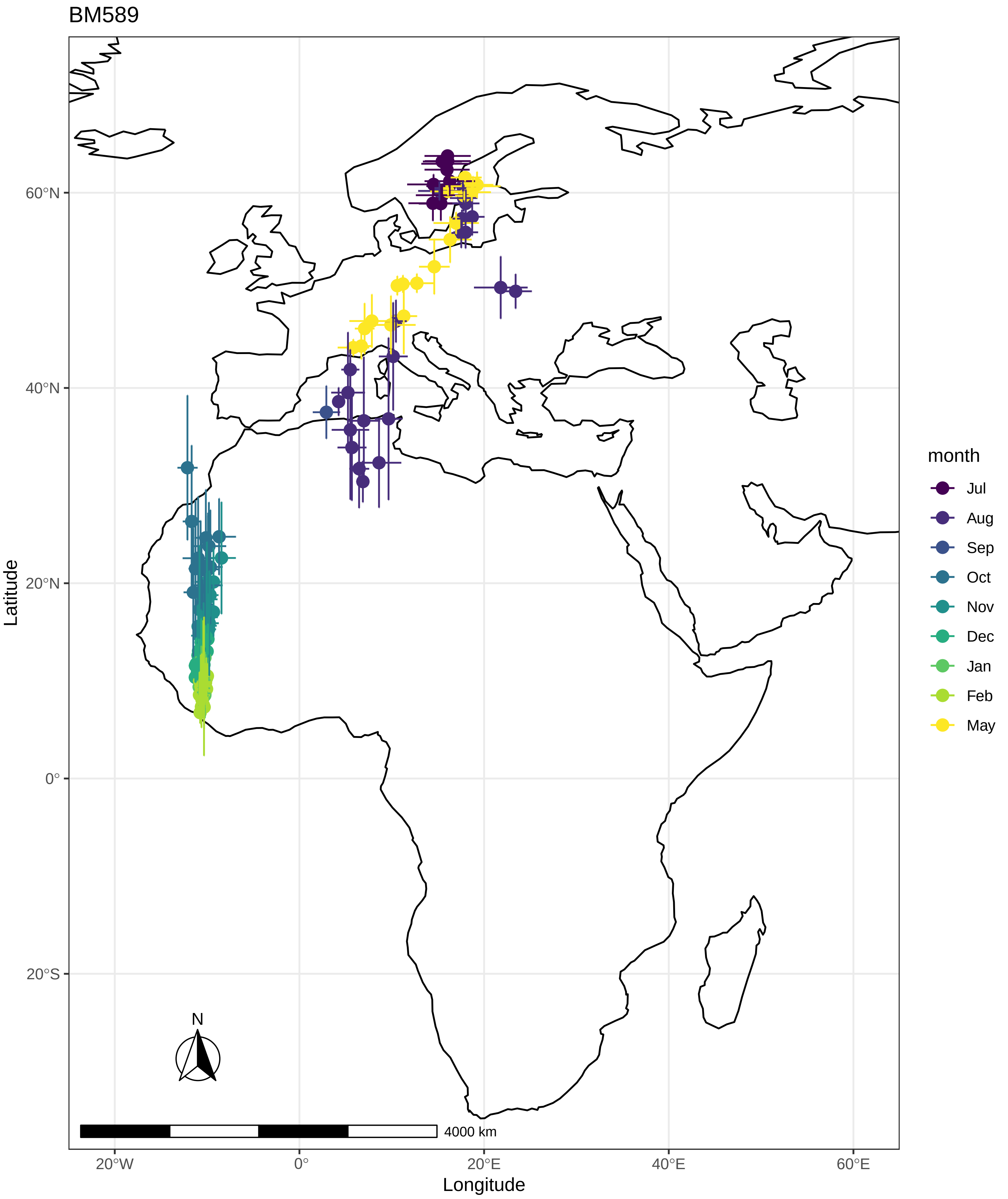

BM589
60°N
40°N
20°N
0°
20°S
20°W
0°
20°E
40°E
60°E
Latitude
Longitude
N
4000 km
month
Jul
Aug
Sep
Oct
Nov
Dec
Jan
Feb
May

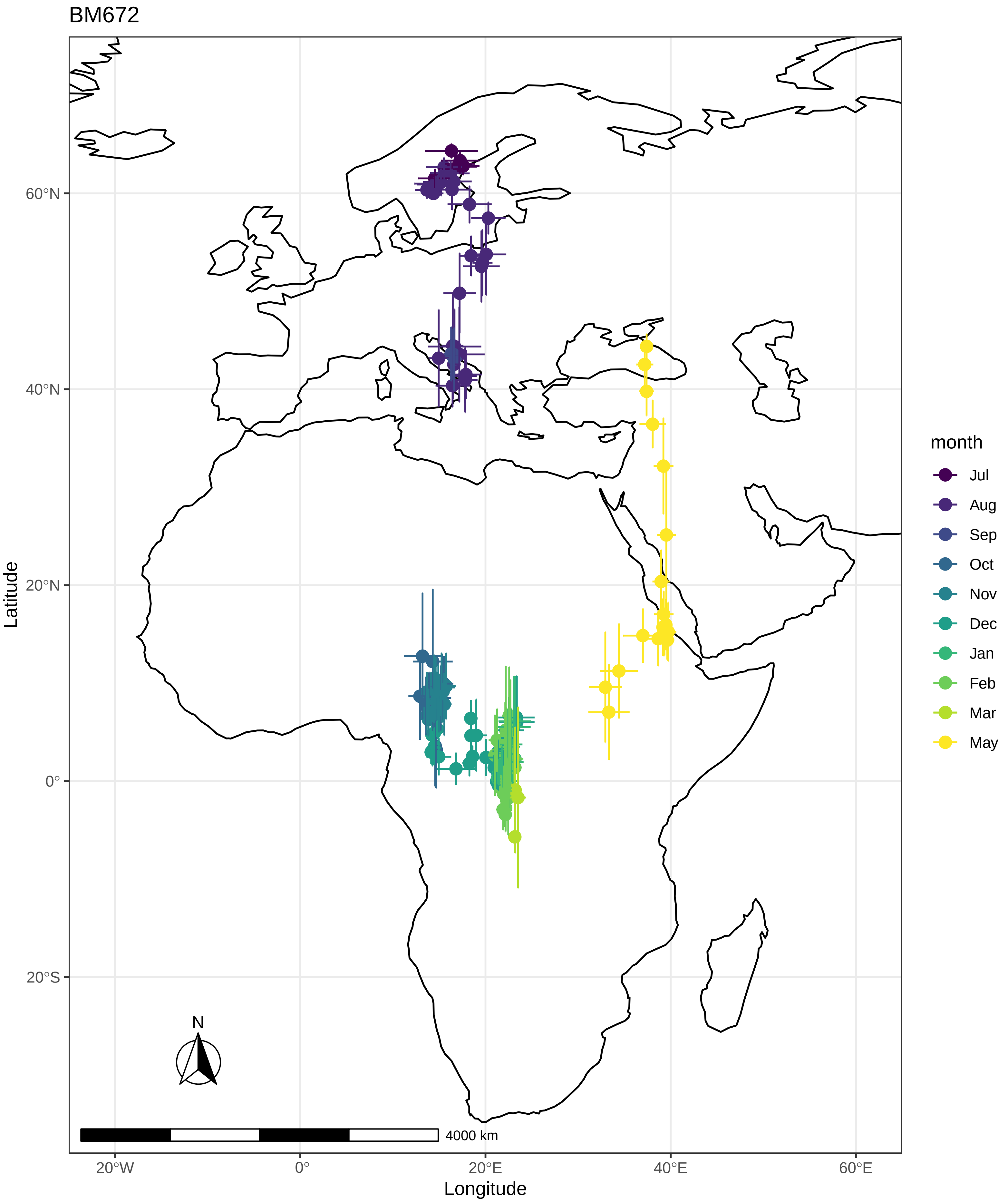

BM672
60°N
40°N
20°N
0°
20°S
20°W
0°
20°E
40°E
60°E
Longitude
Latitude
month
Jul
Aug
Sep
Oct
Nov
Dec
Jan
Feb
Mar
May
N
4000 km

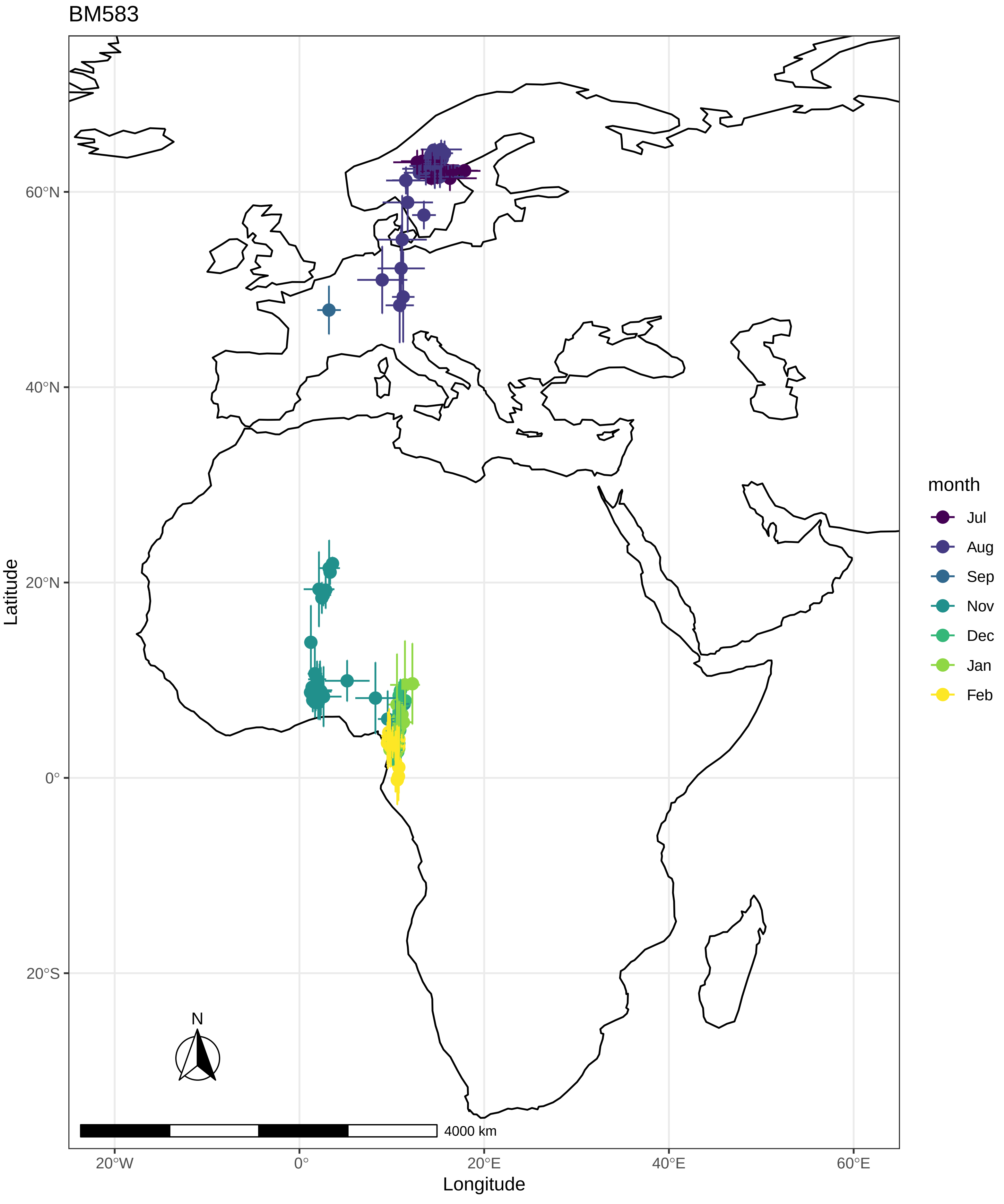
BM583
60°N
40°N
20°N
0°
20°S
20°W
0°
20°E
40°E
60°E
Latitude
Longitude
N
4000 km
month
Jul
Aug
Sep
Nov
Dec
Jan
Feb

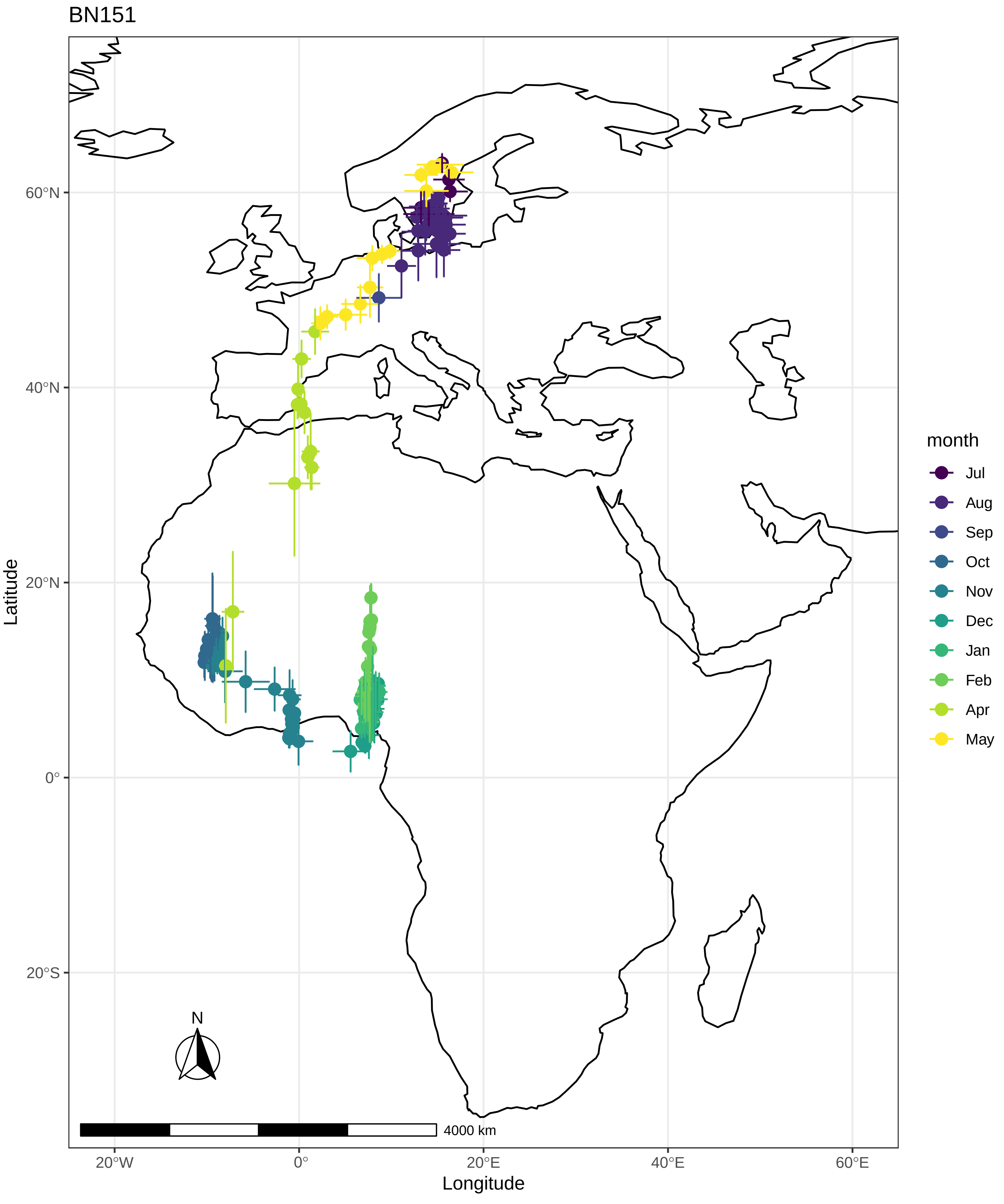

BN151
60°N
40°N
20°N
0°
20°S
Latitude
20°W
0°
20°E
40°E
60°E
Longitude
N
4000 km
month
Jul
Aug
Sep
Oct
Nov
Dec
Jan
Feb
Apr
May

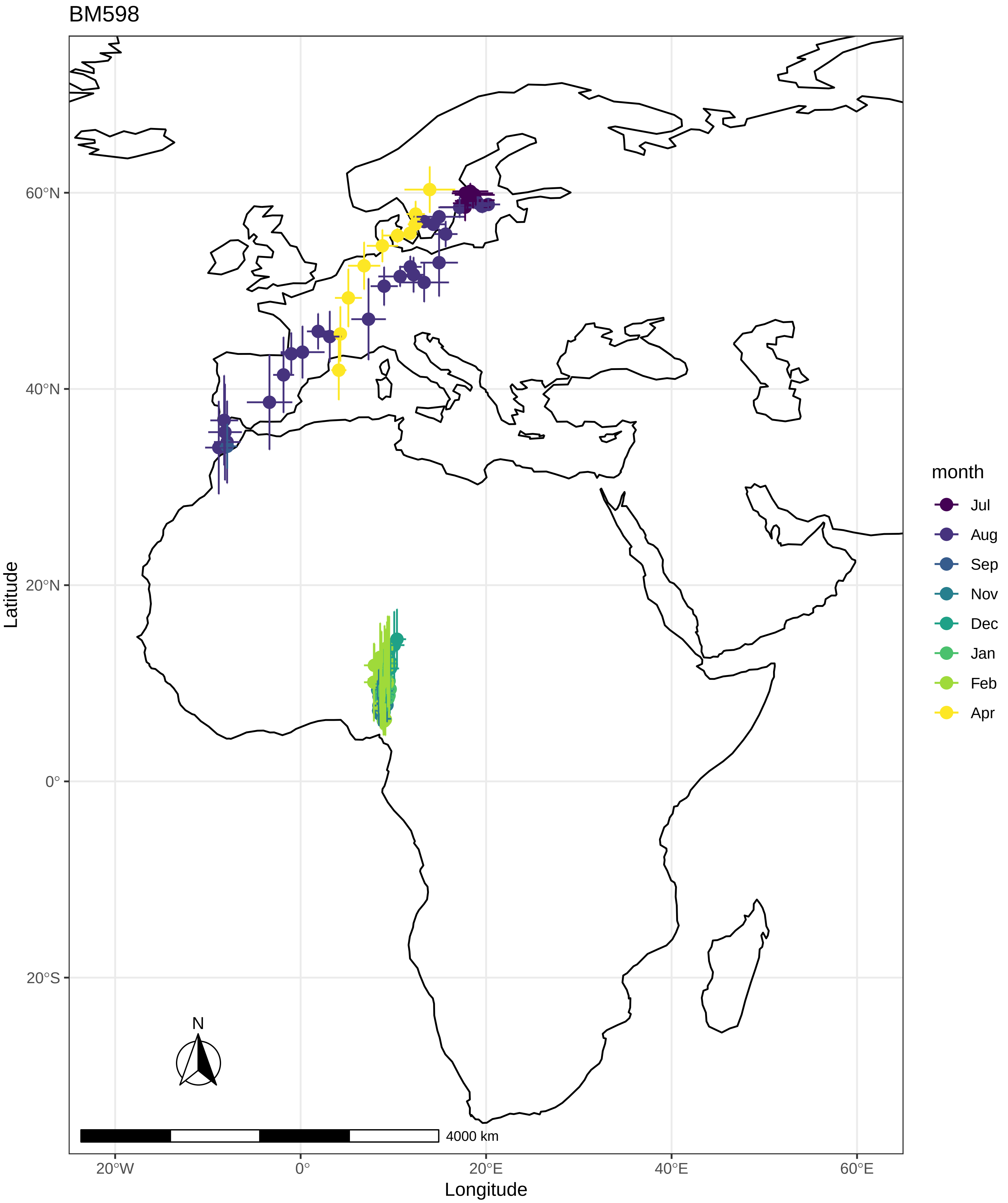
BM598
60°N
40°N
20°N
0°
20°S
Latitude
20°W
0°
20°E
40°E
60°E
Longitude
N
4000 km
month
Jul
Aug
Sep
Nov
Dec
Jan
Feb
Apr

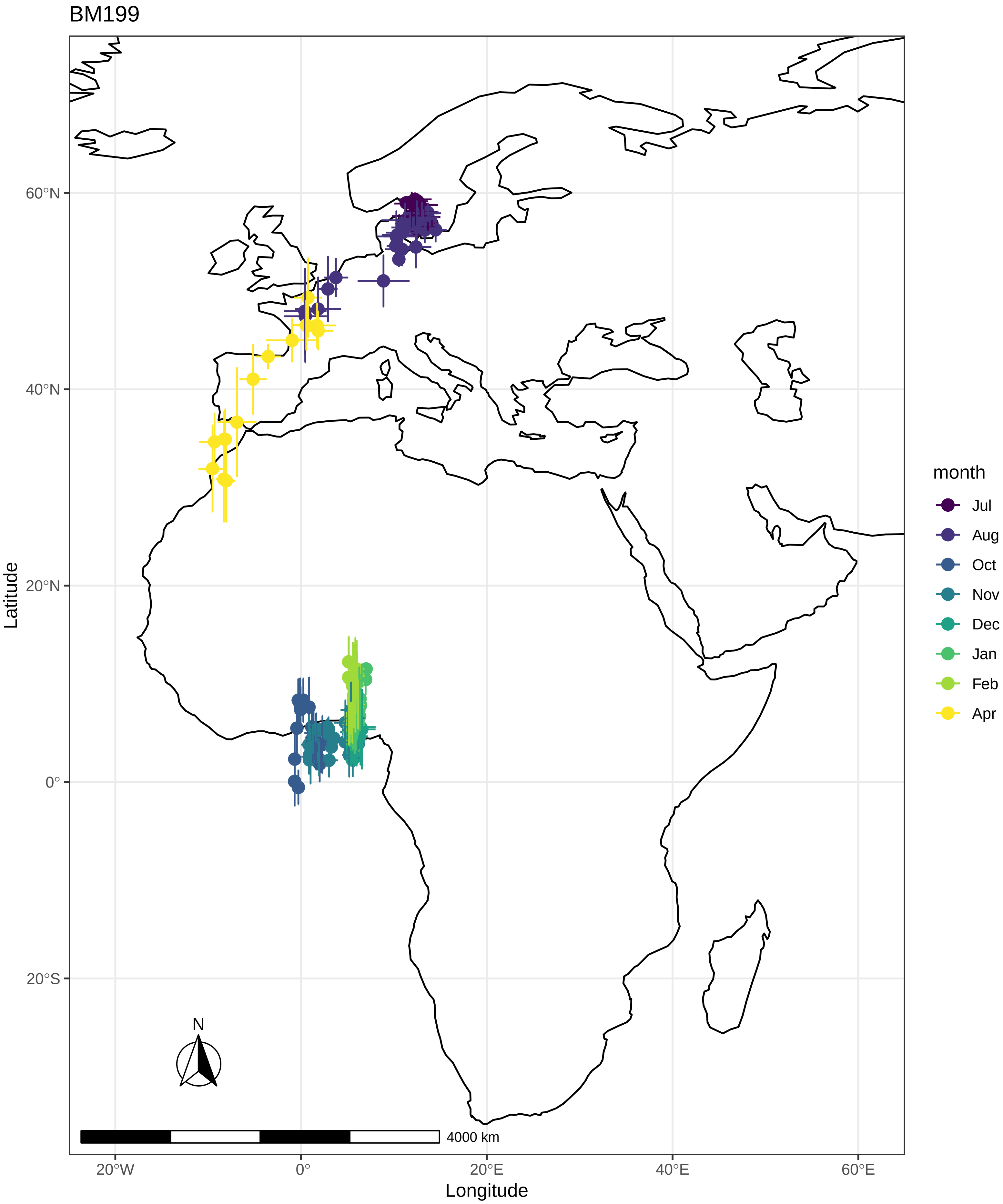

BM199
60°N
40°N
20°N
0°
20°S
Latitude
20°W
0°
20°E
40°E
60°E
Longitude
N
4000 km
month
Jul
Aug
Oct
Nov
Dec
Jan
Feb
Apr

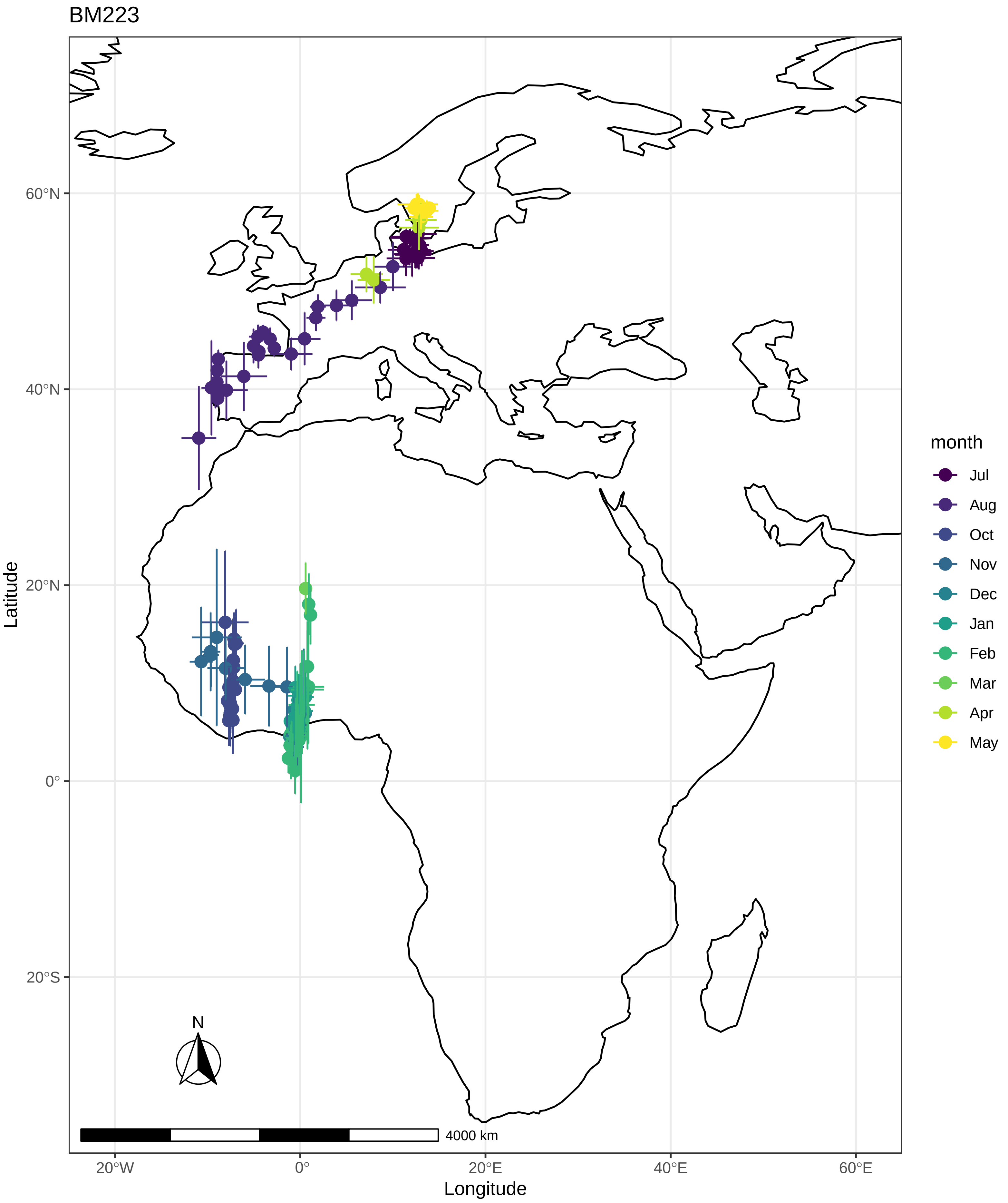

BM223
60°N
40°N
20°N
0°
20°S
Latitude
20°W
0°
20°E
40°E
60°E
Longitude
N
4000 km
month
Jul
Aug
Oct
Nov
Dec
Jan
Feb
Mar
Apr
May

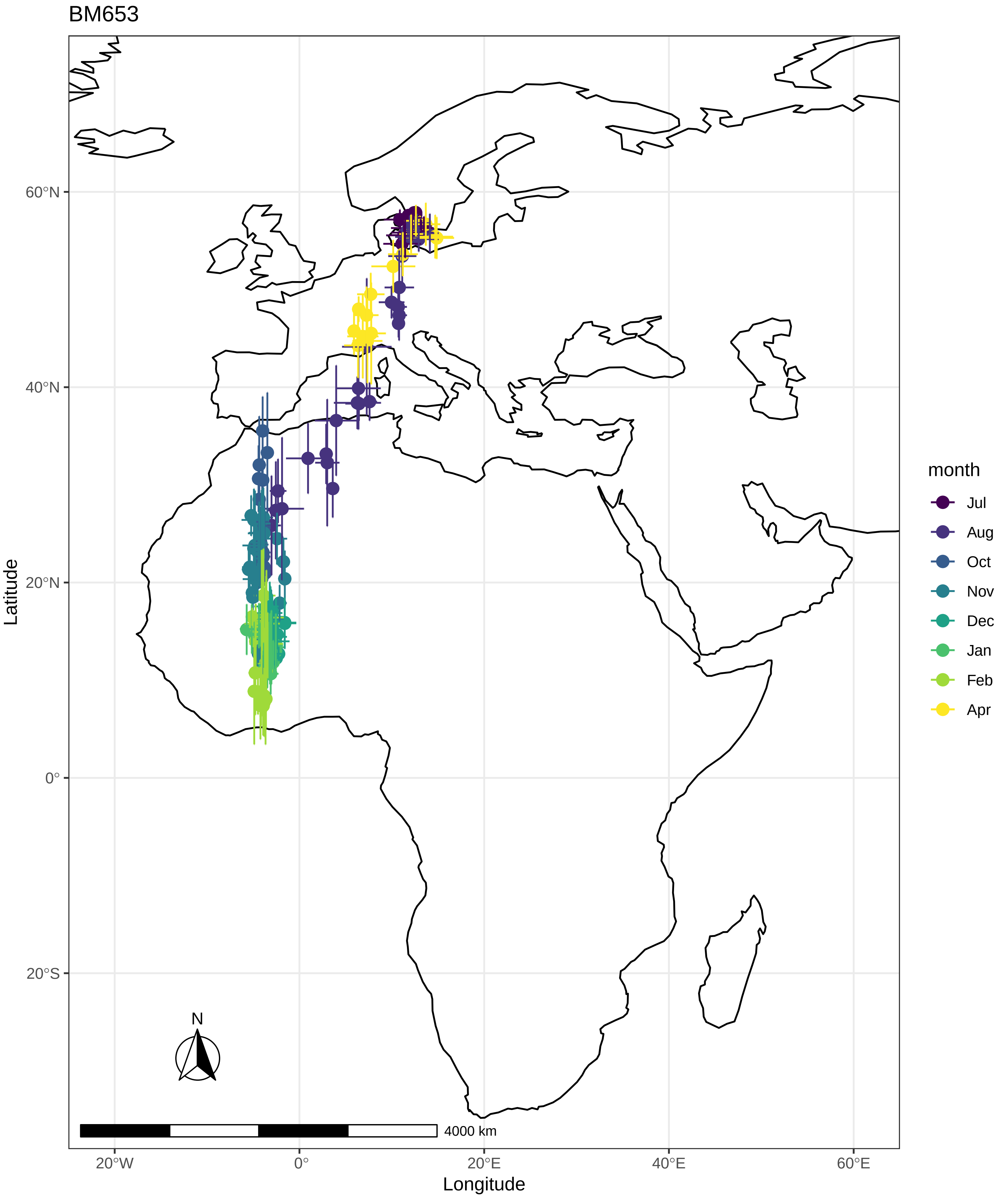

BM653
60°N
40°N
20°N
0°
20°S
Latitude
20°W
0°
20°E
40°E
60°E
Longitude
N
4000 km
month
Jul
Aug
Oct
Nov
Dec
Jan
Feb
Apr

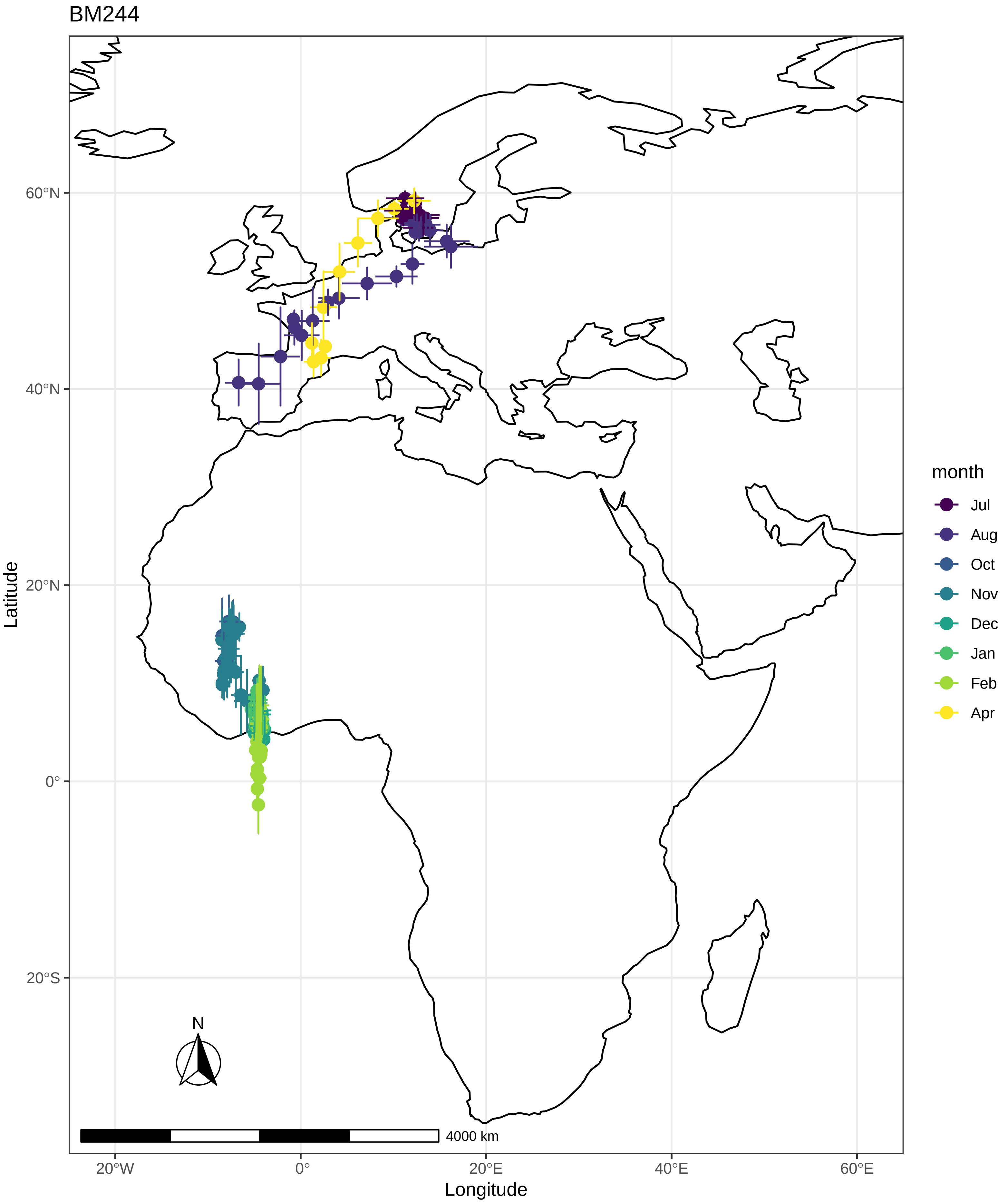

BM244
60°N
40°N
20°N
0°
20°S
20°W
0°
20°E
40°E
60°E
Longitude
Latitude
N
4000 km
month
Jul
Aug
Oct
Nov
Dec
Jan
Feb
Apr

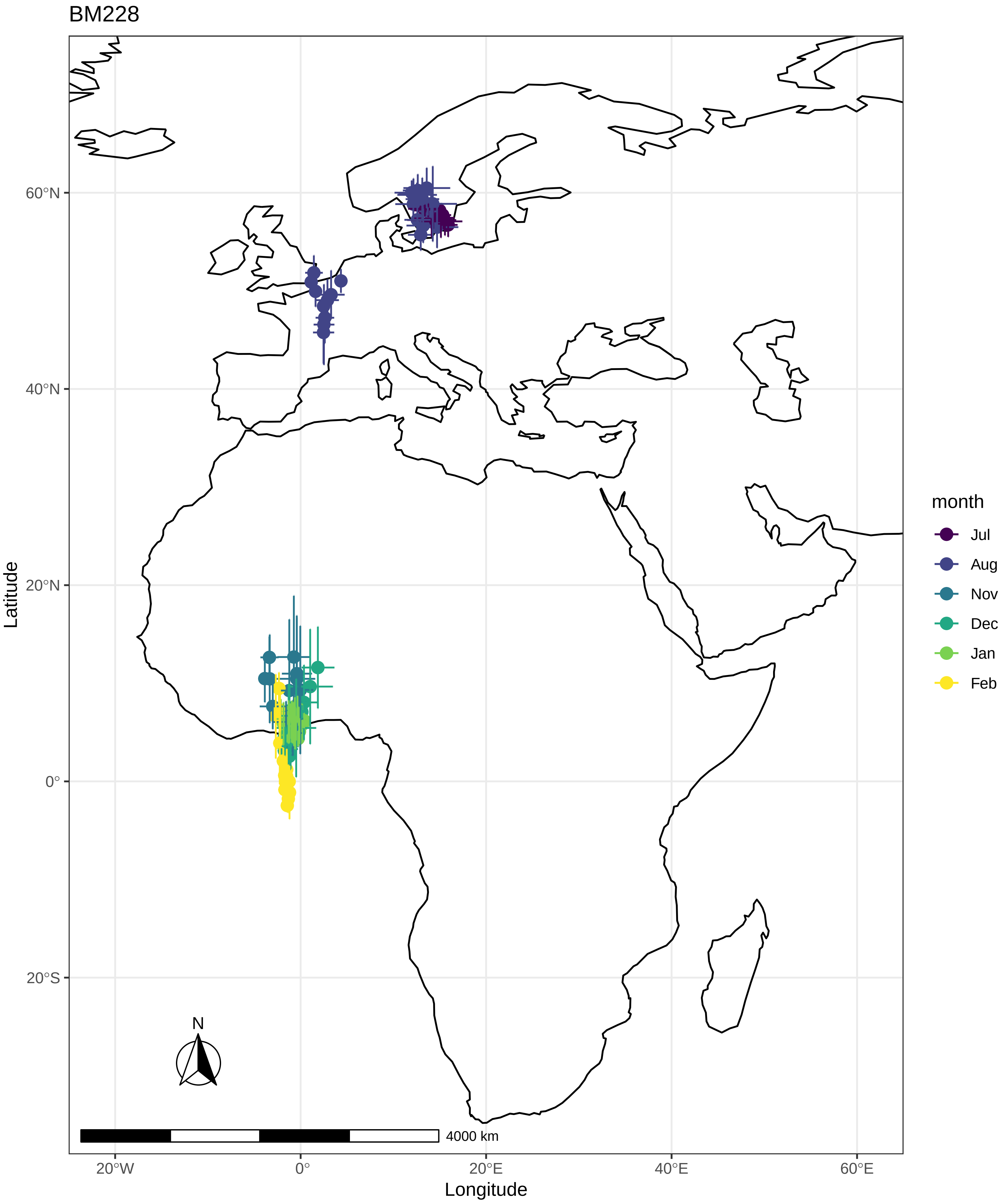
BM228
60°N
40°N
20°N
0°
20°S
Latitude
20°W
0°
20°E
40°E
60°E
Longitude
N
4000 km
month
Jul
Aug
Nov
Dec
Jan
Feb

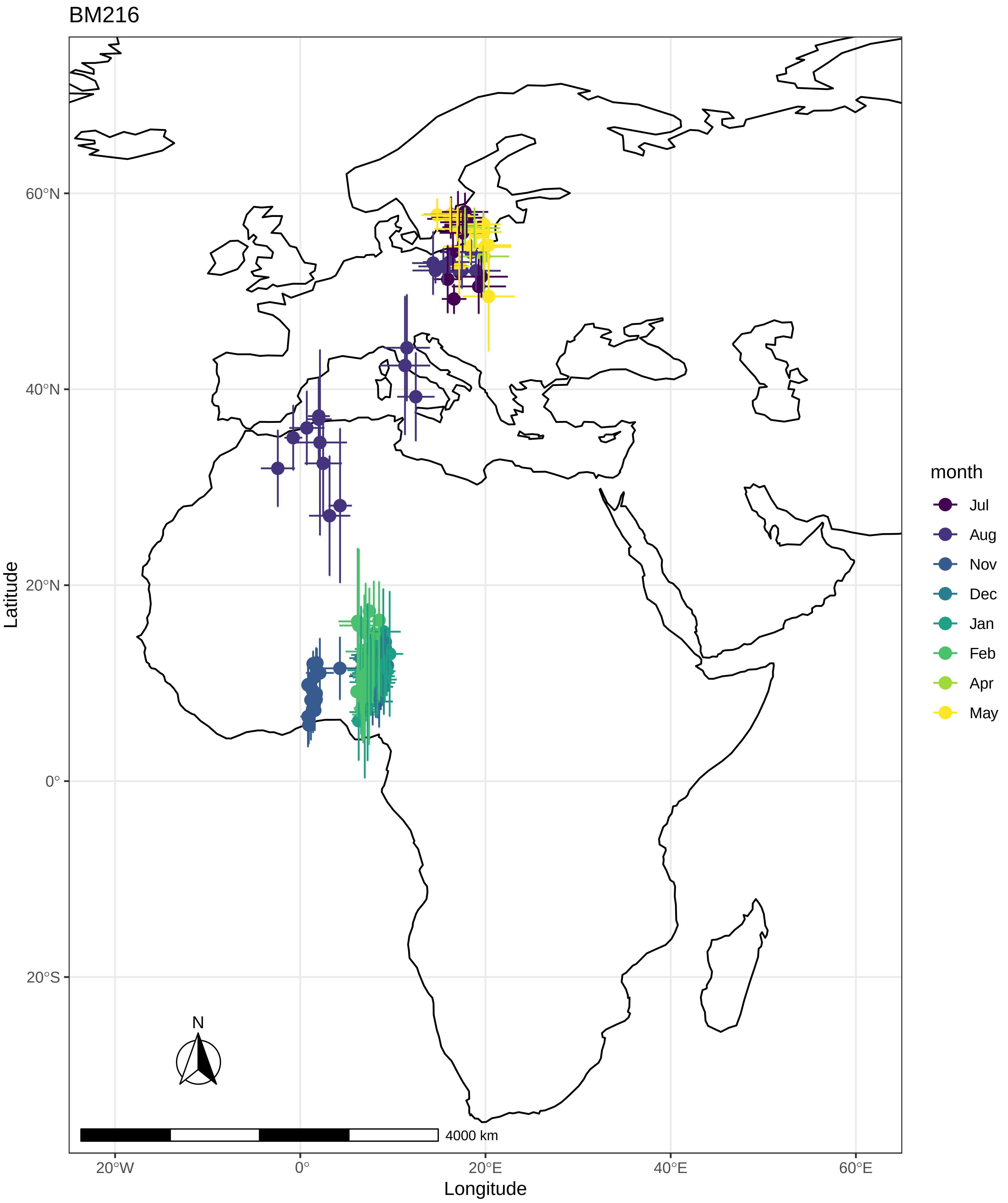

BM216
60°N
40°N
20°N
0°
20°S
Latitude
20°W
0°
20°E
40°E
60°E
Longitude
month
Jul
Aug
Nov
Dec
Jan
Feb
Apr
May
N
4000 km

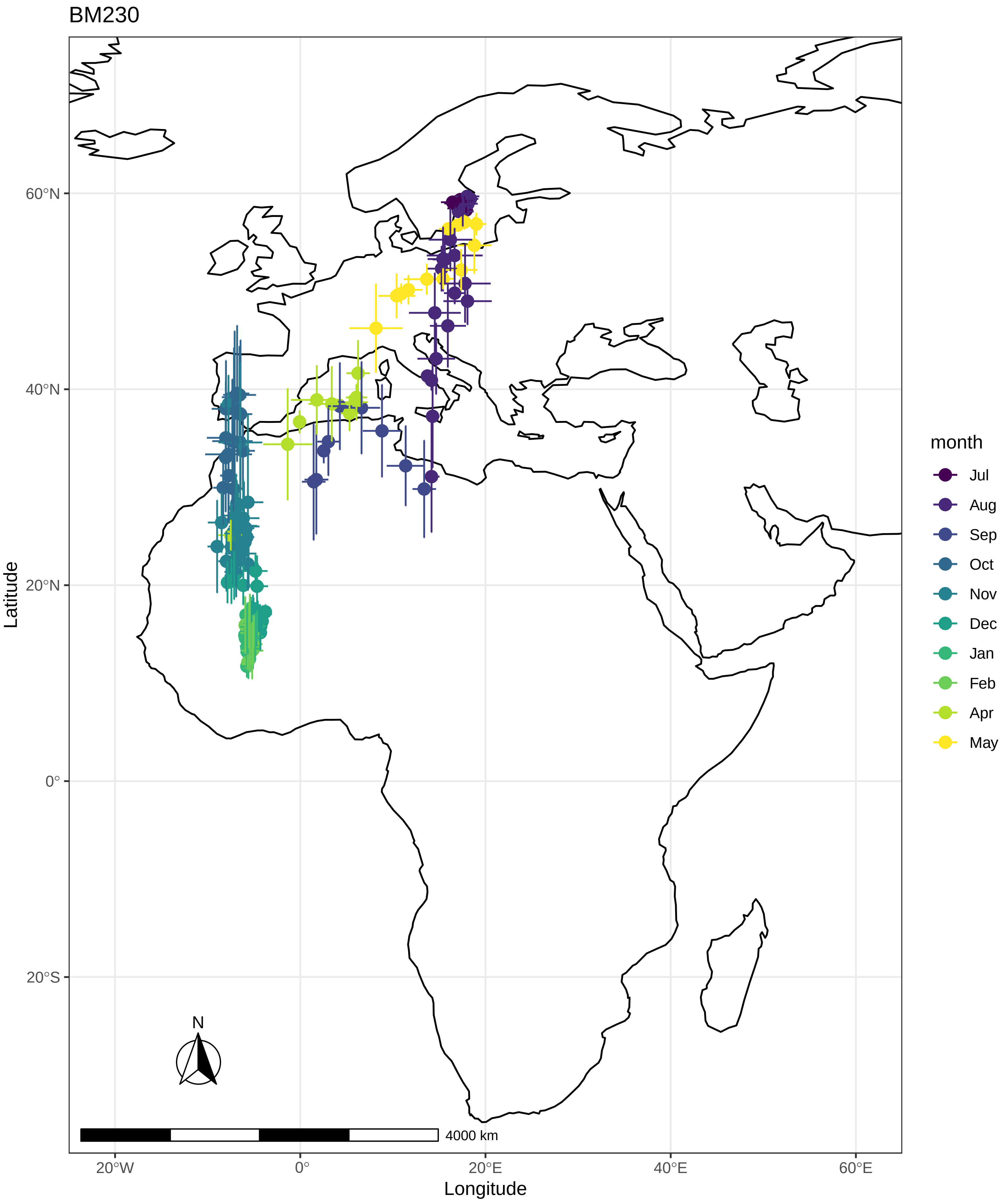
BM230
60°N
40°N
20°N
0°
20°S
Latitude
20°W
0°
20°E
40°E
60°E
Longitude
month
Jul
Aug
Sep
Oct
Nov
Dec
Jan
Feb
Apr
May
N
4000 km

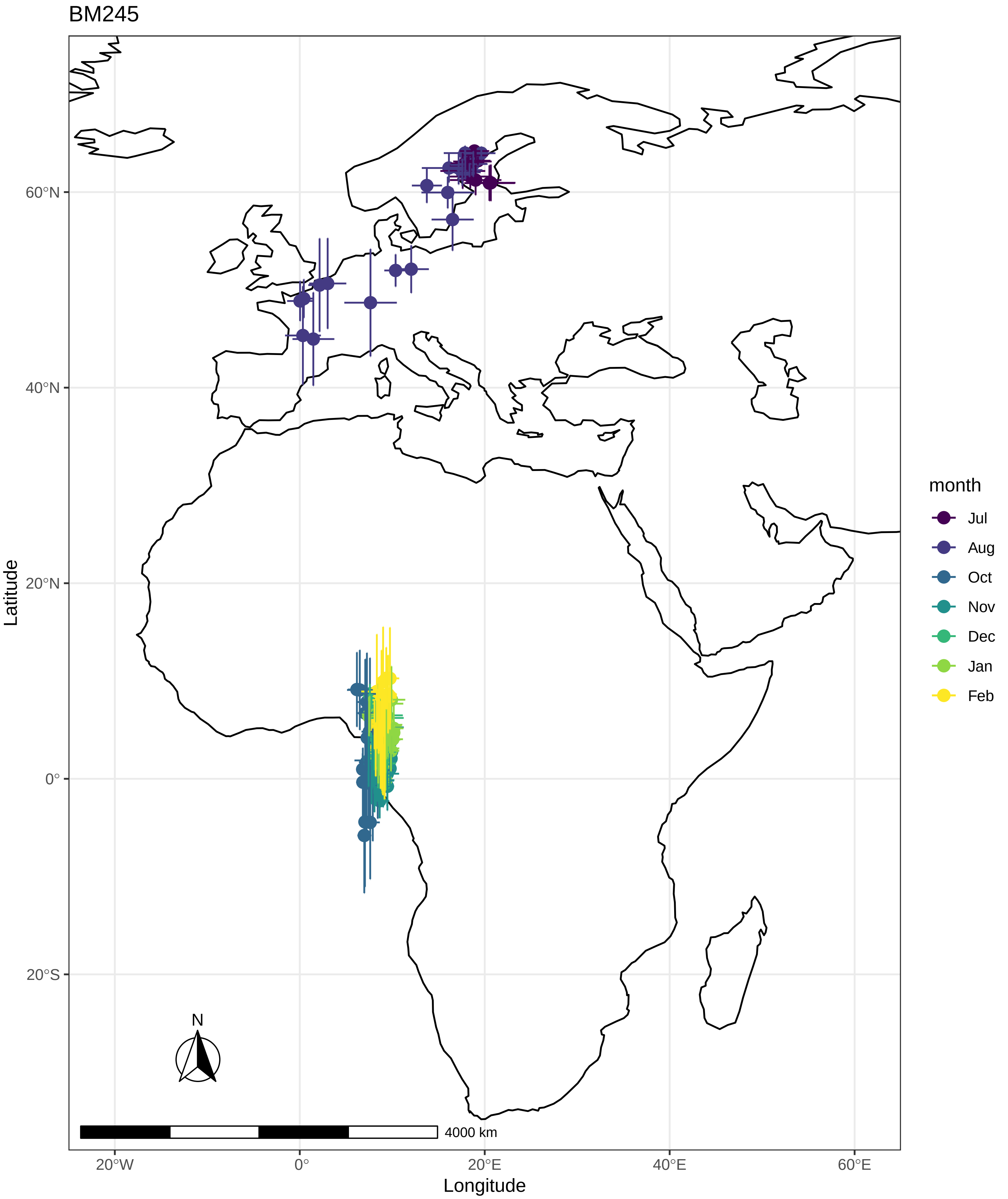
BM245
60°N
40°N
20°N
0°
20°S
Latitude
20°W
0°
20°E
40°E
60°E
Longitude
month
Jul
Aug
Oct
Nov
Dec
Jan
Feb
N
4000 km

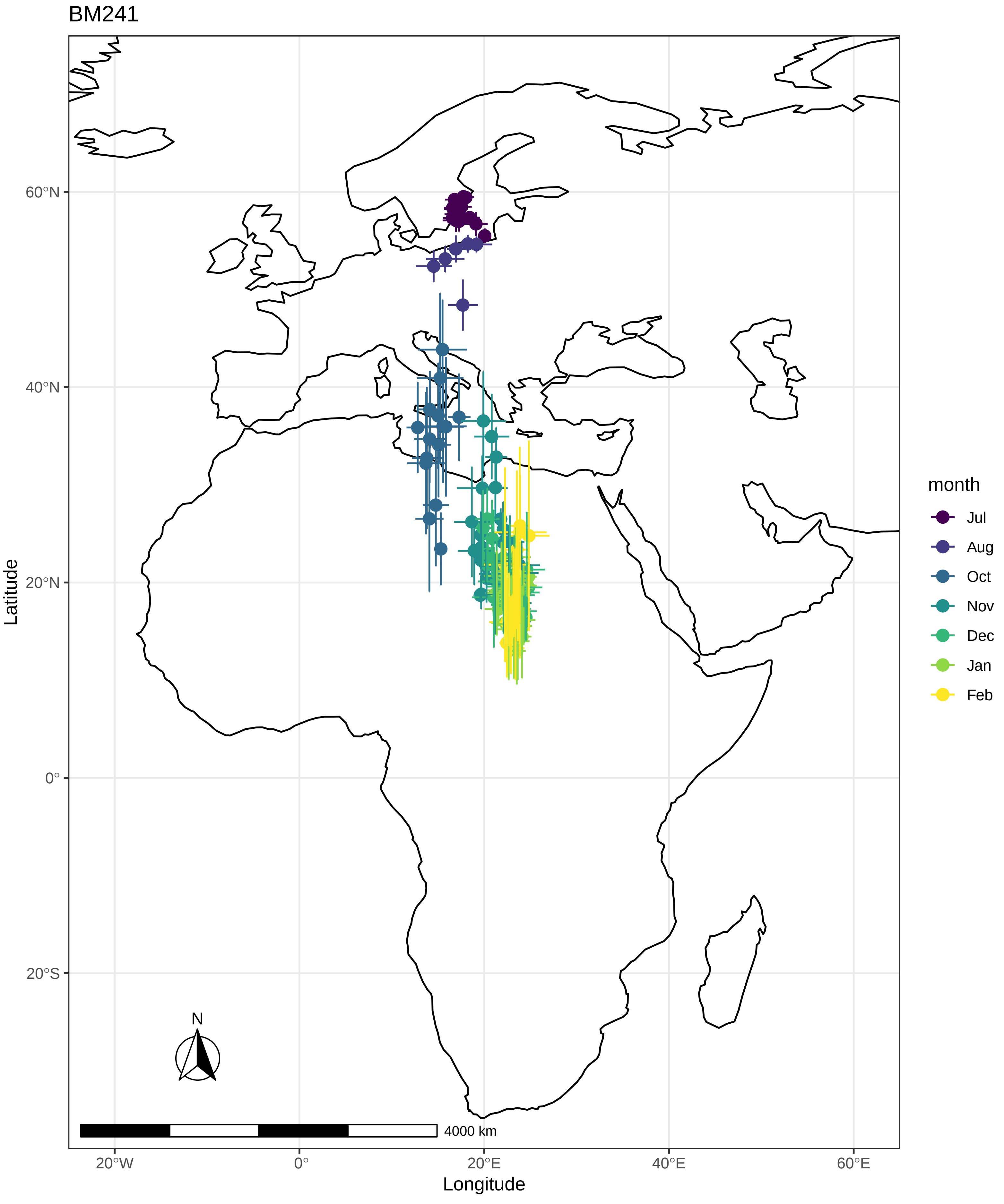
BM241
60°N
40°N
20°N
0°
20°S
Latitude
20°W
0°
20°E
40°E
60°E
Longitude
month
Jul
Aug
Oct
Nov
Dec
Jan
Feb
N
4000 km
